# Utilizing the ^1^H-^15^N NMR Methods for the Characterization of Isomeric Human Milk Oligosaccharides

**DOI:** 10.3390/ijms24032180

**Published:** 2023-01-22

**Authors:** Zsófia Garádi, András Tóth, Tamás Gáti, András Dancsó, Szabolcs Béni

**Affiliations:** 1 Department of Pharmacognosy, Semmelweis University, Üllői út. 26, H-1085 Budapest, Hungary; 2 Directorate of Drug Substance Development, Egis Pharmaceuticals Plc., P.O. Box 100, H-1475 Budapest, Hungary; 3 Servier Research Institute of Medicinal Chemistry (SRIMC), Záhony utca 7, H-1031 Budapest, Hungary

**Keywords:** HMO, carbohydrate analysis, ^1^H-^15^N HSQC-TOCSY, GlcNAc, amino-sugar, *N*-acetyl, NMR resonance assignment

## Abstract

Human milk oligosaccharides (HMOs) are structurally complex unconjugated glycans that are the third largest solid fraction in human milk after lactose and lipids. HMOs are in the forefront of research since they have been proven to possess beneficial health effects, especially on breast-fed neonates. Although HMO research is a trending topic nowadays, readily available analytical methods suitable for the routine investigation of HMOs are still incomplete. NMR spectroscopy provides detailed structural information that can be used to indicate subtle structural differences, particularly for isomeric carbohydrates. Herein, we propose an NMR-based method to identify the major isomeric HMOs containing GlcNAc and/or Neu5Ac building blocks utilizing their amide functionality. Experimental conditions were optimized (H_2_O:D_2_O 9:1 *v*/*v* solvent at pH 3.0) to obtain ^1^H-^15^N HSQC and ^1^H-^15^N HSQC-TOCSY NMR spectra of the aforementioned building blocks in HMOs. Four isomeric HMO pairs, LNT/LNnT, 3’SL/6’SL, LNFP II/LNFP III, and LSTa/LSTb, were investigated, and complete NMR resonance assignments were provided. In addition, ^1^H and ^15^N NMR resonances were found to be indicative of various linkages, thereby facilitating the distinction of isomeric tri-, tetra-, and pentasaccharide HMOs. The rapid growth of HMO products (from infant formulas and dietary supplements to cosmetics) undoubtedly requires expanding the range of applicable analytical methods. Thus, our work provides a ^15^N NMR-based method to advance this challenging field of carbohydrate analysis.

## 1. Introduction

Human milk oligosaccharides (HMOs) are the third most abundant solid components in breastmilk [1]. HMOs possess prebiotic, antiadhesive, immune modulator, and intestinal epithelial cell modulator functions. In addition, they play a significant role in brain development [1]. In the USA, the FDA considers 2′-fucosyllactose (2′-FL), 3-fucosyllactose (3-FL), difucosyllactose (DiFL), lacto-*N*-neotetraose (LNnT), lacto-*N*-tetraose (LNT), 3′-sialyllactose (3′-SL), and 6′-sialyllactose (6′-SL) to be generally recognized as safe (GRAS), while in the EU, EFSA approves these HMOs as novel foods [2]. Recently, various infant formulas and dietary supplements with added HMOs have also become available [3].

**Figure 1:**
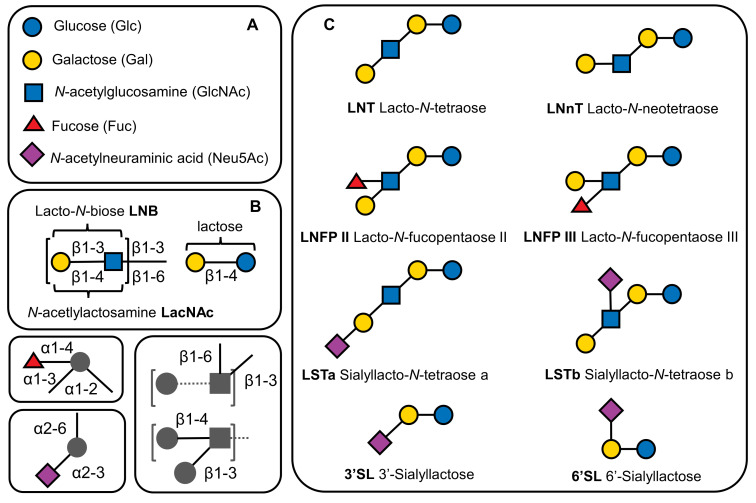
Schematic representation of HMOs. (**A**) The monosaccharide building blocks of HMOs using the symbol nomenclature for glycans (SNFG) [6]. (**B**) General HMO structure, chain elongation possibilities, and the site and modification types of the HMO backbone with Fuc and/or Neu5Ac. (**C**) The structures of the investigated isomeric HMO pairs.

HMOs consist of five monosaccharides: glucose (Glc), galactose (Gal), *N*-acetylglucosamine (GlcNAc), fucose (Fuc), and sialic acid (Sia) derivative *N*-acetylneuraminic acid (Neu5Ac). Each HMO contains lactose (Galβ1-4Glc) at the reducing end, which can be elongated in β1-3- or β1-6 linkage by lacto-*N*-biose (Galβ1-3GlcNAc-, type 1 chain) or *N*-acetyllactosamine (Galβ1-4GlcNAc-, type 2 chain). Lactose or the elongated HMO chain can be fucosylated in α1–2, α1–3, or α1–4 linkage and/or sialylated in α2–3 or α2–6 linkage [4], resulting in more than 200 reported HMO structures [5]. The structural diversity of HMOs, along with the representative compounds investigated herein, can be seen in Figure 1. 

Due to the evidenced structure–function relationship of HMOs, their analytical investigation and structural elucidation have now become essential [7]. However, due to their complex structure, their analytical characterization is challenging. One of the most widely used technique for the structural investigation of HMOs is mass spectrometry (MS). While MS is most often used as a detector in liquid chromatographic separation of HMOs [8], recent developments of novel MS techniques make it also suitable as a sole instrument to distinguish isomeric HMO structures [9]. Dodds and co-workers utilized ion-mobility mass spectrometry (IM-MS) to identify the isomeric pentasaccharides Lacto-N-fucopentaose I and V by using their collision cross-sections (CCSs) [10,11]. The development of various electron-based dissociation methods (ExD) is also at the forefront of MS-based glycoanalytical methods, and they can also be applied to differentiate HMO isomers [9,12,13]. The aforementioned MS-based techniques provide a reliable analytical platform to identify (isomeric) HMOs. These sensitive yet destructive techniques cannot be used routinely in daily analytical practice at present.

Although nuclear magnetic resonance (NMR) spectroscopy is classified as a rather insensitive technique, it provides the most detailed structural information of molecules with subtle structural differences. The similarity of the HMO building block monomers and the various isomeric linkages make the NMR spectroscopic investigation of HMOs challenging. Furthermore, the co-presence of the α and β anomeric form at the reducing end results in resonance duplication in the NMR spectra. A rapid ^1^H NMR method was developed to provide structural information on fucosylated HMOs; thereby, HMO samples were easily classified into one of the four specific milk groups [14]. Beside the structural elucidation of HMOs, ^1^H NMR has also been used in the metabolomic profiling of human milk. Fifteen HMOs were identified, and their concentrations were determined by NMR in colostrum, transition, and mature milk samples [15].

Over a decade ago, the heparin adulteration case drew attention to the wide applicability and performance of the 2D NMR techniques in contaminant detection and identification of carbohydrate drugs [16,17]. This problem highlighted the urgent need for new analytical techniques to discover minor structural differences in polysaccharides. The issue also revealed that the combination of NMR spectroscopy with orthogonal techniques is essential for the identification of structurally related carbohydrates [18].

Observation of the ^15^N isotope brought a new perspective to the NMR spectroscopic characterization of glycans. The presence of *N*-containing monosaccharide building blocks (GlcN, GlcNS, GlcNAc, etc.) enables the use of ^15^N NMR to advance their structural profiling. Typically, ^1^H-^15^N heteronuclear correlations are investigated to provide information about the target molecule or a mixture of compounds [19,20]. In a previous work, Langeslay and co-workers published the optimal conditions for the detection of NH resonances for *N*-sulfoglucosamine residues, thereby opening the door for ^1^H-detected ^15^N NMR experiments on heparin characterization [21]. This approach was used to characterize the microstructure of a particular glycan sequence containing various *N*-sulfoglucosamine units through their ^15^N and ^1^H chemical shifts [22]. In the case of hyaluronan oligosaccharides, high-field NMR was used to characterize their structure, up to hexasaccharides, where the perturbation effect of the adjacent monosaccharides on the ^15^N NMR chemical shift of the GlcNAc units were also investigated [23].

As the vast majority of HMOs contain the NMR active nucleus ^15^N incorporated into *N*-acetylglucosamine and/or *N*-acetylneuraminic acid moieties, we aimed to introduce the ^15^N and ^1^H NMR chemical shifts of GlcNAc or Neu5Ac building blocks into the characterization of HMOs in order to facilitate the identification of isomeric structures.

## 2. Results and Discussion

### 2.1. Optimization of Experimental Conditions

In order to optimize the NMR parameters, as well as the sample preparation, *N*-acetylglucosamine (GlcNAc) standard was used as a model compound. As the amide protons (NH) of the GlcNAc units exchange with that of the solvent, an H_2_O:D_2_O 9:1 (*v/v*) mixture was chosen to ensure the presence of the NH resonance in the ^1^H NMR spectra. The exchange rates of the NH protons are pH-dependent; thus, the ideal pH value was determined first to ensure maximum signal intensity to obtain satisfactory ^15^N-^1^H correlations. The integral value of the ^1^H NMR resonance of the GlcNAc NH proton was therefore monitored in the range of pH 3.0–9.0 using unbuffered samples to exclude any possible exchange-rate-enhancing effect of the buffer components (see Figure S1). As sharper resonances with higher integral values were observed in the range of pH 3.0–5.0, further measurements were performed using phosphate-buffered samples. The increase in buffer concentration (from 5 mM up to 50 mM) resulted in slightly lower NH intensities, especially at less acidic pHs; therefore, pH 3.0 and 5 mM buffer concentration was chosen as a compromise in order to keep the buffer capacity high enough as well. Thereafter, the appropriate pulse sequence was selected to perform water suppression and to minimize the saturation transfer between the solvent and the exchangeable NH resonances. There are several solvent suppression techniques available suitable for the sensitive detection of exchangeable protons [24]. In our case, the presaturation (zgpr) was found to be satisfactory to investigate the amide NHs in the GlcNAc and Neu5Ac residues of the oligosaccharides. As it is one of the simplest and an easily implementable solvent suppression technique, the ^1^H-^15^N-HSQC (hsqcetgpsi2) and ^1^H-^15^N HSQC-TOCSY (hsqcdietgpsi) pulse sequences were combined with presaturation. The software library pulse programs were changed by the separation of the relaxation delay and the presaturation through inserting the d2 delay parameter. While modifying and running the 2D experiments, it was crucial to keep the power level values in the proper range. This d2 parameter was also added to the ^1^H (zgpr) pulse sequence.

### 2.2. Complete ^1^H, ^13^C, and ^15^N Resonance Assignments of the HMO Samples

The ^1^H, ^13^C, and ^15^N NMR resonance assignment of isomeric HMO pairs, LNT/LNnT, LNFP II/LNFP III, LSTa/LSTb, and 3’SL/6’SL, were deduced from the conventional 1D and 2D NMR spectra using 10 mM HMO in H_2_O:D_2_O (9:1) mixture in a 5 mM phosphate-buffered pH 3.0 solution using 0.5 mM DSS as chemical shift reference. 

**Figure 2:**
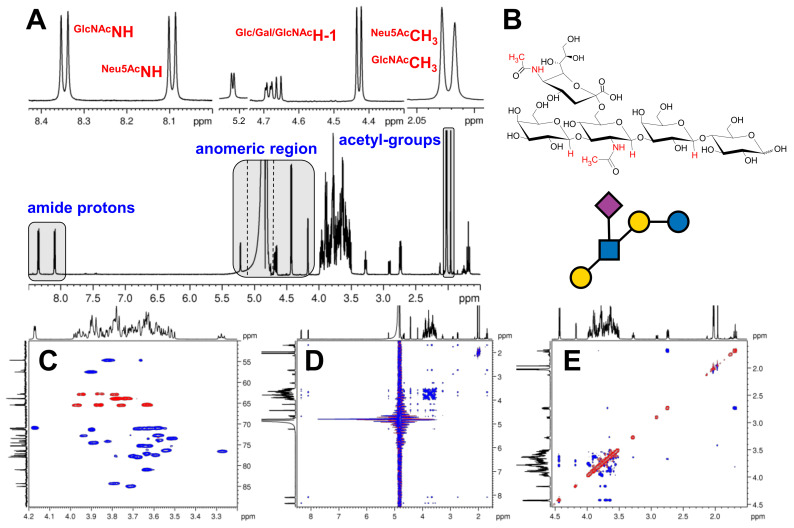
Representative NMR spectra of LSTb (5 mg in 600 µL H_2_O:D_2_O (9:1) solvent using 5 mM phosphate buffer and 0.5 mM DSS reference at pH 3.0). (**A**) The 600 MHz ^1^H NMR spectrum of LSTb using solvent suppression highlighted the characteristic ^1^H spectral regions. (**B**) The molecular structure of LSTb. (**C**) The “carbohydrate” region of ^1^H-^13^C HSQC spectrum of LSTb, (**D**) 2D ^1^H-^1^H TOCSY spectrum of LSTb. (**E**) Selected ^1^H-^1^H 2D ROESY spectral region of LSTb.

Representative spectra showing the resonance assignment workflow is provided for LSTb in Figure 2. The ^1^H NMR spectrum served as a good entry point in all cases (Figure 2A). Besides the most downfield-shifted NH resonance, the anomeric region provided well-resolved resonances; therefore, 1D TOCSY series were recorded to map each building block’s spin system. These 1D series also revealed the multiplicity pattern of each resonance. COSY spectrum provided the ^1^H-^1^H connectivities in each monosaccharide unit. In order the resolve the severe overlap between the resonances in the “CH region”, band-selective ^1^H-^13^C HSQC (Figure 2C) and DEPTQ spectra were also acquired. Finally, the HMBC spectrum confirmed the assignment of the ^1^H and ^13^C resonances (especially in the case of HMOs containing two acetyl moieties). The connectivities of the building blocks were confirmed by 2D ROESY experiment (Figure 2E). The assignment of the amide NH ^1^H resonance was rather obvious, while in the case of LSTa and b (where both GlcNAc and Neu5Ac contain an acetamide moiety), the 2D TOCSY aided the unequivocal assignment of the different NHs (Figure 2D). The ^15^N NMR chemical shifts were deduced from the ^1^H-^15^N HSQC spectrum. The complete resonance assignment of the isomeric HMO pairs can be found in Table 1 and Table 2. ^1^H, ^13^C, ^1^H-^1^H COSY, ^1^H-^13^C HSQC, ^1^H-^13^C HMBC, ^1^H-^1^H TOCSY, ^1^H-^1^H ROESY, ^1^H-^15^N HSQC ^1^H-^15^N HSQC-TOCSY spectra of LNT, LNnT, LNFP II, LNFP III, LSTa, LSTb, 3’SL and 6’SL can be seen in Figures S2–S73.

The Glc unit at the reducing end of each HMO exhibited α/β anomers; therefore, two sets of resonances were assigned to this residue. In a few cases, the H-5 resonance of the β-Glc could not be assigned due to strong overlap. This anomeric splitting can also be observed on the adjacent Gal residue at H-1, H-2, and H-3 and all the ^13^C resonances; furthermore, the anomeric H-1 of the third monosaccharide residue (GlcNAc) still reflects a minor anomeric splitting (although 10 covalent bonds apart from the Glc C-1). The only resonance where no chemical shifts are provided was the H-5 of all Gal residues, as it showed complete overlap under the investigated conditions. Among the studied HMOs, the complete assignment of the GlcNAc unit was feasible, and the fucose unit for LNFP II and LNFP III could completely be assigned. HMOs containing both GlcNAc and Neu5Ac moieties (LSTa and LSTb) show overlapping *N*-acetyl CH_3_ resonances for LSTa, while those are resolved for its isomer LSTb. The non-overlapping acetyl-CH_3_ groups of LSTb could be distinguished and unequivocally assigned by selective HMBC spectrum.

### 2.3. Comparison of the NMR Characteristics of the Isomeric HMOs

#### 2.3.1. LNT–LNnT

**Figure 3:**
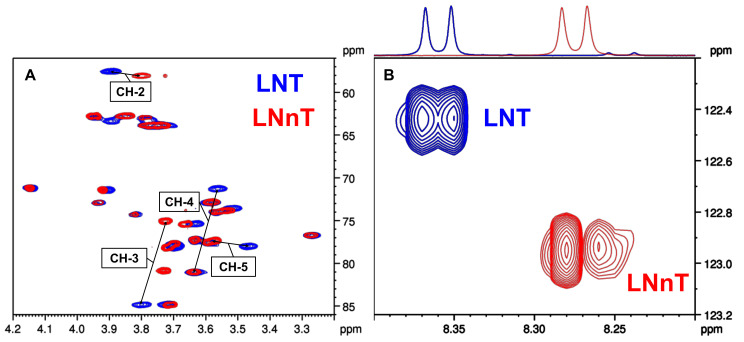
Overlaid 2D NMR spectra of the isomeric LNT and LNnT. (**A**) Overlaid ^1^H-^13^C HSQC spectra of LNT and LNnT, indicating ^1^H and ^13^C NMR chemical shift perturbations of the GlcNAc moiety. (**B**) Overlaid ^1^H-^15^N HSQC spectra of LNT and LNnT, indicating the remarkably different ^1^H-^15^N correlations of the GlcNAc unit.

This pair of isomeric tetrasaccharides possesses the same monosaccharide composition; however, GlcNAc is elongated with Gal at position 3 (type 1 chain) for LNT, and at position 4 (type 2 chain) for LNnT. Comparing the ^1^H chemical shifts of the GlcNAc moiety at positions 2 (Δ*δ*_H-2_ = 0.10 ppm), 3 (Δ*δ*_H-3_ = 0.07 ppm), 4 (Δ*δ*_H-4_ = 0.16 ppm), and 6 (Δ*δ*_H-6b_ = 0.07 ppm), remarkable differences could be observed. Furthermore, the ^13^C resonances at positions 3 (Δ*δ*_C-3_ = 9.8 ppm) and 4 (Δ*δ*_C-4_ = 9.6 ppm) of the GlcNAc unit also reflect the different linkages (see Figure 3A). In addition, a Δ*δ*_NH_= 0.09 ppm difference was also registered for the amide ^1^H resonances of the two isomers, while the ^15^N chemical shifts were also sensitive to the βGal1-3βGlcNAc vs. βGal1-4βGlcNAc linkages, exhibiting a Δ*δ*_N_ = 0.50 ppm difference (Figure 3B).

In the case of the two core disaccharides containing Gal-GlcNAc moiety but differing in the glycosidic linkages, Gal1-3GlcNAc (lacto-*N*-biose, LNB) or Gal1-4GlcNAc (*N*-acetyl-lactosamine, LacNAc), the same chemical shift pattern could be observed for the amide moieties (^1^H and ^15^N) as in their corresponding tetrasaccharides (see Figures S76 and S77 and Table S1).

#### 2.3.2. LNFP II–LNFP III

These isomeric pentasaccharides differ in the attachment of the Fuc and Gal moieties to the GlcNAc residue: while in LNFP II, the Fuc is attached to the GlcNAc at position 4 and Gal at position 3, respectively, in LPFP III, these connectivities show the opposite pattern (see Figure 1). The ^1^H and the ^13^C NMR chemical shifts of the GlcNAc unit at the site of elongation differed considerably: at position 3 (Δ*δ*_H-3_ = 0.20 ppm and Δ*δ*_C-3_ = 1.1 ppm) and at position 4 (Δ*δ*_H-4_ = 0.19 ppm and Δ*δ*_C-4_ = 0.9 ppm) large differences were observed. The structural isomerism was also noticeably reflected by the chemical shift differences of the Fuc moiety (Δ*δ*_H-1_ = 0.10 ppm and Δ*δ*_C-1_ = 0.6 ppm, Δ*δ*_H-2_ = 0.11 ppm). In addition, the Gal moiety exhibited the same phenomena at the site of connection (Δ*δ*_C-1_ = 1.09 ppm). The amide ^1^H resonances showed a difference of Δ*δ*_NH_= 0.03 ppm, while the ^15^N chemical shifts exhibited a Δ*δ*_N_ = 0.12 ppm change.

#### 2.3.3. LSTa–LSTb

This pentasaccharide pair contains two different *N*-acetyl groups, owing to the presence of GlcNAc and Neu5Ac moieties. While LSTa shows a linear structure in which the Neu5Ac unit is connected to the Gal moiety, its isomer LSTb possesses a branched structure bearing a GlcNAc moiety linked to both Gal and Neu5Ac building blocks (see Figure 1). Significant difference was noticed between the ^1^H NMR assignments at position 3 of the Gal unit (Δ*δ*_H-3_ = 0.45 ppm), reflecting the presence/absence of the Neu5Ac unit. Large differences were observed between the ^13^C resonances at positions 2 and 3 of the Gal unit (Δ*δ*_C-2_ = 1.6 ppm and Δ*δ*_C-3_ = 3.0 ppm) and at positions 5 and 6 of the GlcNAc moiety (Δ*δ*_C-5_ = 1.5 ppm and Δ*δ*_C-6_ = 2.2 ppm, respectively). The axial H-3 resonance of Neu5Ac was also found to be a good reporter of the isomerism (Δ*δ*_H-3ax_ = 0.10 ppm). The distinction of the *N*-acetyl groups of GlcNAc and Neu5Ac moieties were deduced from the 2D TOCSY experiment. The differences in the ^1^H chemical shift of the two amides were rather small (Δ*δ*_NH_ = 0.01 for GlcNAc and Δ*δ*_NH_ = 0.02 ppm for Neu5Ac), while a more pronounced shift change was observed for ^15^N (Δ*δ*_N_ = 0.20 and Δ*δ*_N_ = 0.23 ppm for GlcNAc and Neu5Ac, respectively).

#### 2.3.4. 3’SL–6’SL

In these trisaccharide HMOs, the Neu5Ac moiety is connected to the lactose core at different positions. Remarkable chemical shift differences were registered at the Gal unit at positions 3 and 6 in both ^1^H and ^13^C spectra (Δ*δ*_H-3_ = 0.44 ppm and Δ*δ*_C-3_ = 3.0 ppm, Δ*δ*_H-6_ = 0.22 ppm and Δ*δ*_C-6_ = 2.5 ppm). Similar to the LST isomers, the axial H-3 of Neu5Ac was sensitive to the minor structural difference (Δ*δ*_H-3ax_ = 0.06 ppm). The amide ^1^H (Δ*δ*_NH_ = 0.02 ppm) and ^15^N (Δ*δ*_N_ = 0.03 ppm) resonances were found to be less responsive to the different isomeric linkages.

### 2.4. The Use of ^15^N HSQC-TOCSY to Distinguish Isomers

The complete ^1^H, ^13^C, and ^15^N NMR assignment of each individual HMO allows a thorough comparison of the common GlcNAc moiety found in all oligosaccharides (see Table 1 and Table 2). The recorded NMR data demonstrate that significant chemical shift perturbations in both the ^1^H and ^15^N chemical shifts of the *N*-acetyl groups, as well as in the ^1^H resonances of the GlcNAc unit, were observed. In order to combine the perturbation effect on both nuclei in GlcNAc moiety, ^1^H-^15^N HSQC-TOCSY experiments were applied at natural abundance. This experiment detects the protons within the GlcNAc spin system and correlates them to the ^15^N chemical shifts of the acetamide group, thereby reporting on subtle structural changes in the GlcNAc residues in isomeric HMOs. While this experiment suffers from somewhat lower sensitivity than the much simpler ^1^H-^15^N HSQC experiment, this is not a limitation for HMOs, as they are readily available, highly water soluble, and present in high concentrations in various products.

**Figure 4:**
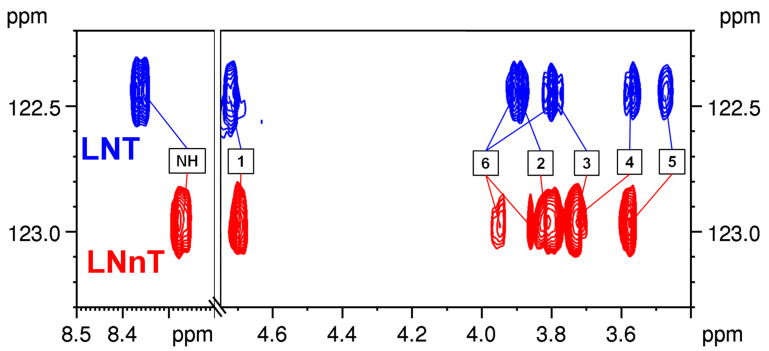
Overlaid ^1^H-^15^N HSQC-TOCSY spectra of LNT and LNnT with the ^1^H NMR assignment of their GlcNAc moiety. (The ^1^H-^15^N HSQC-TOCSY spectrum of LNT and LNnT mixture can be seen in Figure S79).

Figure 4 shows the overlaid ^1^H-^15^N HSQC-TOCSY experiments on the two most common HMO tetrasaccharides, LNT and LNnT. As the ^15^N chemical shifts of the two isomers differ by 0.50 ppm, their GlcNAc spin systems show remarkably different patterns. 

The ^1^H-^15^N HSQC-TOCSY experiment highlights the ^1^H chemical shifts of the complete GlcNAc moiety, and therefore simplifies the crowded “CH region”, and the ^1^H chemical shift patterns indicate the different glycosidic linkages between the terminal Gal and the GlcNAc residues. Combining the ^1^H and ^15^N chemical shifts therefore aids in providing a simple distinction between the two isomers. Compared to LNT and LNnT, the isomeric pentasaccharide pair of LNFP II and LNFP III contains an extra Fuc unit linked to the GlcNAc moiety at different positions. Their ^1^H-^15^N HSQC-TOCSY spectra (shown in Figure S75) can also facilitate their distinction. This approach is also applicable for the sialylated HMOs due to the presence of the acetamide group of Neu5Ac. The sialyllactose isomers 3’SL and 6’SL exhibit different ^1^H NMR patterns when edited according to their ^15^N chemical shifts (shown in Figure S74).

**Figure 5:**
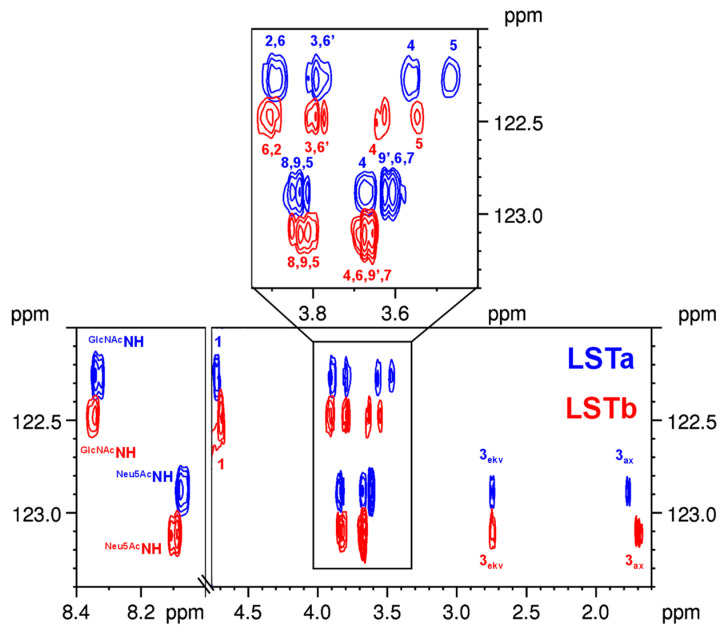
Overlaid ^1^H-^15^N HSQC-TOCSY spectra of isomeric LSTa and LSTb with the ^1^H NMR assignment of their GlcNAc and Neu5Ac moieties (The full spectra can be seen in Figure S80).

The ^1^H-^15^N HSQC-TOCSY experiment can enhance the distinction of isomeric structures even more when bearing two reporter moieties (GlcNAc and Neu5Ac). For LSTa and LSTb, the overlaid HSQC-TOCSY spectra show two separate regions according to the GlcNAc and Neu5Ac units (see Figure 5). Although the Neu5Ac is connected through a glycosidic bond to two distinct building blocks (either Gal or GlcNAc), this minor structural difference can still be observed in the HSQC-TOCSY row of each of the pentasaccharides (see remarkable differences in chemical shifts of H-3_ax_). While both LSTa and LSTb carry the same LNT-type skeleton, their other reporter moiety, GlcNAc, is still sensitive to the sialylation. The chemical shift perturbations observed for GlcNAc can be seen in Figure 5, and thereby contribute to a successful identification of the isomeric HMOs.

In order to check the limitation of the proposed method, the ^15^N HSQC spectrum of para-LNnH hexasaccharide was also registered under unbuffered condition. This HMO also contains two reporter moieties, but in this case, both are GlcNAc residues. Unfortunately, no significant chemical shift differences were observed for the ^1^H and ^15^N nuclei of the two acetamide groups. The ^1^H-^15^N HSQC cross-peaks completely overlapped and appeared as a single ^1^H-^15^N correlation at the same chemical shift values as that of LNnT (see Figure S78); therefore, ^1^H-^15^N HSQC-TOCSY experiment failed to distinguish GlcNAc residues in para-LNnH at 600 MHz. 

A further model compound (a non-HMO tetrasaccharide) possessing repeating GlcNAc units (*N*,*N*′,*N*″,*N*‴-tetraacetylchitotetraose) was also investigated. Although the ^15^N and ^1^H resonances of alpha and beta anomers of GlcNAc at the reducing end differed significantly, further GlcNAc residues showed no chemical shift perturbation, and therefore their ^15^N and ^1^H resonances could not be resolved (see Figures S81 and S82).

## 3. Materials and Methods

### 3.1. Chemicals and Reagents

Human milk oligosaccharide standards, lacto-*N*-tetraose (LNT), lacto-*N*-neotetraose (LNnT), sialyllacto-*N*-tetraose a (LSTa), sialyllacto-*N*-tetraose b (LSTb), lacto-*N*-fucopentaose II (LNFP II), lacto-*N*-fucopentaose III (LNFP III), 3′-sialyllactose (3′-SL), and 6′-sialyllactose (6′-SL), were kindly provided by DSM (Hørsholm, Denmark). Deuterium oxide D_2_O (99.9 atom% D), 3-(trimethylsilyl)-1-propanesulfonic acid sodium salt (DSS), and *N*-acetyl-D-glucosamine were purchased from Merck (Darmstadt, Germany). *N*,*N*′,*N*″,*N*‴-Tetraacetylchitotetraose was purchased from Carbosynth (Compton, United Kingdom). Sodium phosphate was purchased from Reanal (Budapest, Hungary). Water was prepared freshly, using Select Fusion water purification system (SUEZ Water Technologies & Solutions, Feasterville-Trevose, PA, USA). The SevenCompact S210 pH-meter and standard reference buffers (pH 2.00, 4.01, and 7.00) were purchased from Mettler-Toledo (Greifensee, Switzerland).

### 3.2. NMR Spectoscopic Measurements

All NMR spectra were recorded on a Bruker Avance III HD 600 (^1^H: 600.05 MHz, ^13^C: 150.89 MHz, ^15^N: 60.81 MHz) instrument (Bruker Biospin GmbH, Rheinstetten, Germany) equipped with a Prodigy cryo-probehead. The pulse programs were taken from the Bruker software library (TopSpin 3.5 pl 7). The NMR spectra were acquired in standard 5 mm NMR tubes at 295 K. The ^13^C and ^1^H chemical shifts (*δ*) are given in ppm relative to the internal standard (DSS), while the coupling constants (*J*) are given in Hz. The heteronuclear ^15^N NMR experiments were performed at natural abundance. The ^15^N resonances were deduced from the heteronuclear 2D spectra (shifts relative to liquid ammonia). The resonance assignment of each HMO was achieved by using the following pulse sequences available in the Bruker software library and were used without any modification: ^13^C (zgpg30), DEPTQ (deptqgpsp), ^1^H-^1^H COSY (cosygpmfqf), ^1^H-^13^C HSQC (hsqcedetgpsisp2.2), ^1^H-^13^C selective HSQC (shsqcetgpsisp2.2), ^1^H-^13^C HMBC (hmbcgplpndqf), ^1^H-^1^H ROESY (roesyphpr.2), ^1^H-^1^H TOCSY (mlevphpr.2), selective gradient TOCSY (seldigpzs), and selective gradient ^1^H-^1^H ROESY (selrogp) experiments. The appropriate amounts of HMOs (10 mM) were dissolved in an H_2_O:D_2_O (9:1) mixture containing 5 mM NaH_2_PO_4_, and the pH was adjusted to 3.0 using 0.1 M HCl. As NMR chemical shift reference, 0.5 mM DSS was used for both ^1^H and ^13^C nuclei.

#### NMR Experiments with Solvent Suppression

The ^1^H spectra (using the zgprd pulse program) of the HMO samples were recorded in a 7211.539 Hz (12.0 ppm) spectral window (SWH), with 32 scans (NS) and 16 dummy scans (DS). The size of the fid was 32768 (TD), and receiver gain was set automatically. Transmitter frequency offset (O1) was set based on the water ^1^H resonance. The 90° pulse (P1) was set by calibration for each sample individually. The delay parameters (D1, D2) were set to 2 s. 

The ^1^H-^15^N-HSQC spectra of the HMO samples were acquired with a spectral width (SWH) 6009.615 Hz (10.0 ppm) in F2 and 608.147 Hz (10.0 ppm) in F1. A total of 64 scans (NS) and 16 dummy scans (DS) were recorded, along with 2048 complex points (TD) in F2 and 64 in F1. Transmitter frequency offset (O1) parameters in F2 and F1 were set based on preliminary trial experiments. In addition, 2 s relaxation delays were used (D1, D2).

The ^1^H-^15^N HSQC-TOCSY spectra of the HMO samples were acquired with a spectral width (SWH) 6009.615 Hz (10.0 ppm) in F2 and 608.025 Hz (10.0 ppm) in F1. From 650 to 715 scans (NS) and 16 dummy scans (DS) were recorded, along with 2048 complex points (TD) in F2 and 64 in F1. Transmitter frequency offset (O1) parameters in F2 and F1 were set based on preliminary trial experiments. In addition, 2 s relaxation delays were used as D1 and D2, while D9 was set to 120 ms.

## 4. Conclusions

In this study, 2D heteronuclear ^15^N NMR experiments were used for the characterization of isomeric HMOs for the first time. To compare their NMR characteristics in aqueous solution, ^1^H-^15^N HSQC and ^1^H-^15^N HSQC-TOCSY experiments were utilized using water suppression. Their *N*-acetyl group containing the building blocks GlcNAc and/or Neu5Ac were thoroughly investigated to gain unique structural information for the major HMOs. Complete ^1^H, ^13^C, and ^15^N NMR resonance assignments were achieved for the HMOs: LNT, LNnT, 3’SL, 6’SL, LNFP II, LNFP III, LSTa, and LSTb in H_2_O:D_2_O 9:1 at pH 3.0. Our approach to distinguish isomeric HMOs relied on the combination of complete ^1^H and ^15^N NMR chemical shift pattern of GlcNAc and/or Neu5Ac moieties. As shown herein, the investigated isomeric HMO pairs showed remarkably different ^15^N NMR resonances (obtained from the ^1^H-^15^N HSQC experiment). The highest chemical shift perturbation was observed between the tetrasaccharides LNT and LNnT bearing a GlcNAc unit, while the ^15^N NMR chemical shifts of the Neu5Ac moiety in 3’SL and 6’SL trisaccharides were less responsive to the minor structural difference. When considering both ^1^H and ^15^N NMR chemical shifts of the GlcNAc and/or Neu5Ac moieties (obtained from the ^1^H-^15^N HSQC-TOCSY experiment), the distinction of the structural isomers was unambiguous. This NMR-based method offers a straightforward approach for the identification of the most common human milk oligosaccharides and may thereby contribute to the analytical investigation of HMO standards, HMO products, or even human milk.

## Figures and Tables

**Table 1:** The complete ^1^H, ^13^C, and ^15^N resonance assignment of LNT, LNnT, LNFP III, and LNFP III in H_2_O: D_2_O 9:1 *v*/*v* solvent at pH 3.0.

	LNT			LNnT			LNFP II			LNFP III		
	^1^H	m, *J* [Hz]	^13^C/^15^N	^1^H	m, *J* [Hz]	^13^C/^15^N	^1^H	m, *J* [Hz]	^13^C/^15^N	^1^H	m, *J* [Hz]	^13^C/^15^N
	**α-Glc**											
**1**	5.22	d, 3.7	94.6	5.22	d, 3.7	94.6	5.22	d, 3.7	94.6	5.22	d, 3.7	94.6
**2**	3.57	dd, 9.9, 3.8	74.0	3.57	dd, 9.9, 3.8	74.0	3.57	dd, 9.9, 3.8	74.0	3.57	dd, 9.9, 3.8	74.0
**3**	3.82	t, 9.5	74.2	3.82	t, 9.3	74.2	3.82	t, 9.4	74.2	3.82	t, 9.4	74.2
**4**	3.63	t, 9.5	81.0	3.63	t, 9.3	81.0	3.63	t, 9.4	80.9	3.63	t, 9.4	81.0
**5**	3.94	m	72.8	3.94	m	72.8	3.94	m	72.8	3.94	m	72.8
**6**	3.86	m	62.7	3.86	m	62.7	3.85	m	62.7	3.85	m	62.7
	**β-Glc**											
**1**	4.66	d, 7.9	98.5	4.66	d, 7.9	98.5	4.66	d, 7.9	98.5	4.66	d, 7.9	98.5
**2**	3.27	m	76.6	3.27	m	76.6	3.27	m	76.6	3.27	m	76.6
**3**	3.63	m	77.1	3.63	m	77.1	3.63	m	77.1	3.63	m	77.1
**4**	3.64	m	80.9	3.64	m	80.9	3.63	m	81.0	3.64	m	80.9
**5**	n.a.		n.a.	n.a.		n.a.	n.a.		n.a.	n.a.		n.a.
**6a**	3.95	dd, 12.0, 2.0	62.9	3.95	dd, 12.0, 2.0	62.9	3.95	dd, 12.1, 2.0	62.8	3.95	dd, 12.0, 2.0	62.8
**6b**	3.79	dd, 12.2, 5.0		3.79	dd, 12.2, 5.0		3.79	dd, 12.1, 5.0		3.79	dd, 12.0, 5.0	
	**β-Gal**											
**1**	4.43/4.43	d, 7.7	105.60/57	4.43/4.43	d, 7.9	105.61/58	4.42/4.42	d, 7.9	105.62/58	4.42/4.42	d, 7.9	105.61/58
**2**	3.60/3.58	dd, 9.8, 7.8	72.80/77	3.60/3.58	dd, 9.8, 7.9	72.76/73	3.59/3.57	dd, 9.8, 7.9	72.75/73	3.59/3.57	dd, 9.8, 7.9	72.76/73
**3**	3.74/3.72	t, 3.0	84.72/69	3.72/3.71	t, 3.0	84.78/76	3.72/3.70	t, 3.0	84.81/78	3.71/3.69	t, 3.0	84.81/78
**4**	4.15	d, 3.2	71.13/10	4.15	d, 3.2	71.14/12	4.15	d, 3.4	71.12/10	4.15	d, 3.4	71.19/09
**5**	n.a.		n.a.	n.a.		n.a.	n.a.		n.a.	n.a.		n.a.
**6**	3.76	m	63.78/77	3.76	m	63.78/77	3.76	m	63.79/78	3.76	m	63.79/77
	**β-GlcNAc**											
**1**	4.72/4.72	d, 8.4	105.3	4.69/4.69	d, 8.3	105.46	4.69/4.69	d, 8.5	105.4	4.70/4.70	d, 8.5	105.3
**2**	3.90	m	57.5	3.80	m	58.0	3.95	m	58.6	3.96	m	58.7
**NH**	8.36	d, 9.6	122.48	8.27	d, 9.5	122.98	8.44	d, 9.8	122.39	8.41	d, 9.8	122.27
**CO**			117.7			177.7			177.5			177.5
**CH_3_**	2.02	s	25.0	2.03	s	25.0	2.03	s	25.1	2.02	s	25.0
**3**	3.80	t, 9.6	84.8	3.73	m	75.0	4.07	t, 9.6	78.6	3.87	m	77.5
**4**	3.57	t, 9.6	71.2	3.73	m	80.8	3.75	t, 9.6	74.8	3.94	m	75.7
**5**	3.47	ddd, 10.0, 5.0, 2.3	77.9	3.58	m	77.3	3.53	dt, 9.5, 3.0	77.9	3.57	m	77.8
**6a**	3.90	m	63.3	3.95	dd, 12.2, 2.0	62.6	3.94	m	62.4	3.97	m	62.4
**6b**	3.78	dd, 12.5, 5.3		3.85	dd, 12.2, 4.7		3.86	dd, 12.5, 3.2		3.87	m	
	**β-Gal**											
**1**	4.43	d, 8.4	106.2	4.47	d, 7.8	105.53	4.50	d, 7.7	105.55	4.45	d, 7.8	104.46
**2**	3.52	dd, 9.8, 7.8	73.5	3.54	dd, 9.8, 7.8	73.8	3.48	dd, 9.8, 7.7	73.3	3.49	dd, 9.8, 7.9	73.8
**3**	3.64	dd, 10.0, 3.0	75.3	3.67	dd, 10.0, 3.3	75.3	3.62	dd, 9.8, 3.4	75.1	3.65	dd, 9.8, 3.3	75.3
**4**	3.90	d, 3.4	71.3	3.92	d, 3.5	71.3	3.88	d, 3.5	71.2	3.90	d, 3.4	71.2
**5**	n.a.		n.a.	n.a.		n.a.	n.a.		n.a.	n.a.		n.a.
**6**	3.76	m	63.8	3.75	m	63.84	3.72	m	64.5	3.72	m	64.3
							**α-Fuc**					
**1**							5.02	d, 4.0	100.7	5.12	d, 4.0	101.3
**2**							3.80	dd, 10.5, 4.0	70.6	3.69	dd, 10.4, 4.0	70.5
**3**							3.88	dd, 10.3, 3.3	72.0	3.90	dd, 10.4, 3.3	72.0
**4**							3.79	d, 3.3	74.8	3.79	d, 3.3	74.2
**5**							4.87	o.l.	69.6	4.83	o.l.	69.4
**CH_3_**							1.17	d, 6.7	18.1	1.17	d, 6.6	18.0

n.a.: not assigned; o.l.: overlapped.

**Table 2:** The complete ^1^H, ^13^C, and ^15^N resonance assignment of LSTa, LSTb, 3’SL, and 6’SL in H_2_O: D_2_O 9:1 *v*/*v* solvent at pH 3.0.

	LSTa			LSTb			3’SL			6’SL		
	^1^H	m, *J* [Hz]	^13^C/^15^N	^1^H	m, *J* [Hz]	^13^C/^15^N	^1^H	m, *J* [Hz]	^13^C/^15^N	^1^H	m, *J* [Hz]	^13^C/^15^N
	**α-Glc**											
**1**	5.22	d, 3.7	94.6	5.22	d, 3.8	94.6	5.22	d, 3.8	94.6	5.22	d, 3.7	94.6
**2**	3.57	dd, 9.9, 3.8	74.0	3.57	dd, 9.9, 3.8	74.0	3.57	dd, 9.9, 3.8	74.0	3.60	dd, 9.8, 3.8	73.9
**3**	3.82	t, 9.4	74.2	3.82	t, 9.4	74.2	3.83	t, 9.4	74.2	3.84	t, 9.3	74.4
**4**	3.63	t, 9.4	81.1	3.63	t, 9.4	81.1	3.66	t, 9.4	80.9/80.8	3.61	t, 9.3	82.4/82.3
**5**	3.94	m	72.8	3.94	m	72.8	3.94	m	72.8	3.95	m	72.7
**6**	3.86	m	62.7	3.86	m	62.7	3.88	m	62.7	3.88	m	62.9
**β-Glc**												
**1**	4.66	d, 8.0	98.5	4.66	d, 7.9	98.5	4.66	d, 8.0	98.6	4.66	d, 8.0	98.4
**2**	3.27	m	76.6	3.28	m	76.6	3.28	dd, 9.0, 8.0	76.7	3.30	t, 8.6	76.6
**3**	3.63	m	77.2	3.63	m	77.2	3.63	t, 9.2	77.1	3.63	m	77.4
**4**	3.64	m	81.0	3.64	m	81.0	3.67	dd, 9.5, 8.3	80.9/80.8	3.61	m	82.4/82.3
**5**	n.a.		n.a.	n.a.		n.a.	3.59	ddd, 9.5, 5.0, 2.0	77.5	3.63	m	77.4
**6a**	3.95	dd, 12.0, 2.0	62.8	3.95	dd, 12.0, 2.0	62.9	3.96	dd, 12.3, 2.0	62.8	3.95	dd, 12.3, 2.0	63.0
**6b**	3.79	dd, 12.2, 5.0		3.79	dd, 12.2, 5.0		3.82	dd, 12.3, 5.0		3.79	dd, 12.3, 4.4	
**β-Gal**												
**1**	4.43/4.43	d, 7.9	105.60/58	4.43/4.43	d, 7.7	105.60/57	4.52	d, 7.7	105.3	4.42	d, 7.9	105.93/90
**2**	3.60/3.58	dd, 9.8, 7.9	72.80/78	3.59/3.57	dd, 9.8, 7.9	72.78/74	3.57	dd, 9.9, 7.9	72.2	3.53	dd, 9.8, 7.9	73.6
**3**	3.74/3.72	t, 2.9	84.65/63	3.72/3.70	t, 3.3	85.04/84.99	4.11/4.10	t, 3.0	78.21/20	3.67/3.65	t, 3.0	75.2
**4**	4.15	d, 3.2	71.12/10	4.17	d, 3.3	70.97/94	3.95	d, 3.0	70.2	3.93	d, 3.3	71.33/31
**5**	n.a.		n.a.	n.a.		n.a.	n.a.		n.a.	n.a.		n.a.
**6**	3.78	m	63.79/78	3.78	m	63.92/91	3.74	m	63.85/84	3.96	m	66.3
										3.59		
	**β-GlcNAc**											
**1**	4.73/4.72	d, 8.4	105.2	4.69/4.68	d, 8.54	105.4						
**2**	3.90	m	57.4	3.98	m	57.5						
**NH**	8.34	d, 9.6	122.30	8.35	d, 9.6	122.50						
**CO**			177.68			177.67						
**CH_3_**	2.02	s	25.1/24.8	2.02	s	25.0						
**3**	3.80	t, 9.5	84.9	3.79	t, 9.5	84.4						
**4**	3.57	t, 9.5	71.2	3.63	t, 9.5	71.1						
**5**	3.47	ddd, 10.0, 5.3, 2.2	77.9	3.54	ddd, 9.8, 5.2, 2.2	76.4						
**6a**	3.90	m	63.3	3.97	dd, 10.8, 5.7	65.5						
**6b**	3.78	dd, 12.0, 5.3		3.76	d, 10.8							
	**β-Gal**											
**1**	4.50	d, 7.8	106.1	4.43	d, 7.7	106.1						
**2**	3.54	dd, 9.7, 8.0	71.9	3.52	dd 10.0, 7.8	73.5						
**3**	4.08	dd, 9.7, 3.0	78.3	3.63	dd, 10.0, 3.3	75.3						
**4**	3.93	d, 3.0	70.0	3.90	d, 3.4	71.4						
**5**	n.a.		n.a.	n.a.		n.a.						
**6**	3.72	m	63.8	3.78/3.74	m	63.9						
	**α-Neu5Ac**											
**1**			176.5			176.1			176.5			176.10/09
**2**			102.3			102.9			102.4			102.9
**3α**	1.78	t, 12.1	42.5	1.68	t, 12.1	42.8	1.80	t, 12.2 dd, 12.4, 4.7	42.3	1.74	t, 12.2	42.8
**3β**	2.75	dd, 12.4, 4.7		2.74	dd, 12.3, 4.7		2.75			2.70	dd, 12.4, 4.6	
**4**	3.68	ddd, 12.4, 4.7, 2.2	71.2	3.68	m	71.1	3.68	ddd, 11.7, 9.7, 4.7	71.2	3.65	ddd, 11.7, 9.7, 4.6	71.2
**5**	3.84	m	54.4	3.82	m	54.7	3.85	m	54.5	3.85	m	54.6
**CH_3_**	2.02	s	25.1/24.8	2.03	s	24.8	2.03	s	24.8	2.02	s	24.8
**NH**	8.07	d, 9.3	122.91	8.09	d, 9.3	123.14	8.08	d, 9.3	122.98	8.06	d, 9.4	123.01
**CO**			177.72			177.79			177.8			
**6**	3.62	dd, 10.4, 1.8	75.5	3.66	dd, 10.4, 1.8	75.2	3.62	dd, 10.4, 1.8	75.6	3.72	dd, 10.4, 1.7	75.2
**7**	3.59	d, 9.0	70.8	3.58	d, 9.0	71.0	3.59		70.9	3.56	d, 9.0	71.2
**8**	3.87	m	74.7	3.88	m	74.5	3.88	m	74.6	3.89	m	74.6
**9a**	3.85	m	65.3	3.87	m	65.4	3.87	m	65.4	3.88	m	65.5
**9b**	3.64	m		3.63	m		3.64	m		3.64	m	

n.a.: not assigned.

## Data Availability

Not applicable.

## Supplementary Materials

The following supporting information can be downloaded at: https://www.mdpi.com/article/10.3390/ijms24032180/s1.
