# Supplementary material for: Utilizing the 1H-15N NMR Methods for the Characterization of Isomeric Human Milk Oligosaccharides
Source: Int J Mol Sci. 2023 Jan 22;24(3):2180. doi: 10.3390/ijms24032180 (PMC9917050; doi:10.3390/ijms24032180)
Supplement: Supplementary file 1 [file ijms-24-02180-s001.zip › ijms-2118680-supplementary.pdf]

Utilizing the ^{1}H-^{15}N NMR methods for the characterization of isomeric human milk oligosaccharides

Zsófia Garádi[1,2], András Tóth[1], Tamás Gáti[3], András Dancsó[2], Szabolcs Béni[1]

[1]Department of Pharmacognosy, Semmelweis University, Üllői út. 26, H-1085 Budapest, Hungary

[2]Directorate of Drug Substance Development, Egis Pharmaceuticals Plc, P.O. Box 100, H-1475 Budapest, Hungary

[3]Servier Research Institute of Medicinal Chemistry (SRIMC), Záhony utca 7, H-1031 Budapest, Hungary

Table of contents

Figure S1. Selected regions of the ^{1}H NMR spectra of N-acetylglucosamine (GlcNAc) standard in H_2O:D_2O 9:1 v/v unbuffered solution, in the range of pH 3.0-9.0. Anomeric NH proton resonances of GlcNAc: β = 8.22 ppm, α = 8.12 ppm. 5

Figure S2. ^{1}H NMR spectrum of LNT (H_2O:D_2O 9:1 v/v solvent at pH 3.0) 5

Figure S3. ^{13}C NMR spectrum of LNT (H_2O:D_2O 9:1 v/v solvent at pH 3.0) 6

Figure S4. ^{1}H -^{1}H COSY spectrum of LNT (H_2O:D_2O 9:1 v/v solvent at pH 3.0) 6

Figure S5. ^{1}H -^{13}C HSQC spectrum of LNT (H_2O:D_2O 9:1 v/v solvent at pH 3.0) 7

Figure S6. ^{1}H -^{13}C HMBC spectrum of LNT (H_2O:D_2O 9:1 v/v solvent at pH 3.0) 7

Figure S7. ^{1}H -^{1}H TOCSY spectrum of LNT (H_2O:D_2O 9:1 v/v solvent at pH 3.0) 8

Figure S8. ^{1}H -^{1}H ROESY spectrum of LNT (H_2O:D_2O 9:1 v/v solvent at pH 3.0) 8

Figure S9. ^{1}H -^{15}N HSQC spectrum of LNT (H_2O:D_2O 9:1 v/v solvent at pH 3.0) 9

Figure S10. ^{1}H -^{15}N HSQC-TOCSY spectrum of LNT (H_2O:D_2O 9:1 v/v solvent at pH 3.0) 9

Figure S11. ^{1}H NMR spectrum of LNnT (H_2O:D_2O 9:1 v/v solvent at pH 3.0) 10

Figure S12. ^{13}C NMR spectrum of LNnT (H_2O:D_2O 9:1 v/v solvent at pH 3.0) 10

Figure S13. ^{1}H -^{1}H COSY spectrum of LNnT (H_2O:D_2O 9:1 v/v solvent at pH 3.0) 11

Figure S14. ^{1}H -^{13}C HSQC spectrum of LNnT (H_2O:D_2O 9:1 v/v solvent at pH 3.0) 12

Figure S15. ^{1}H -^{13}C HMBC spectrum of LNnT (H_2O:D_2O 9:1 v/v solvent at pH 3.0) 12

Figure S16. ^{1}H -^{1}H TOCSY spectrum of LNnT (H_2O:D_2O 9:1 v/v solvent at pH 3.0) 13

Figure S17. ^{1}H -^{1}H ROESY spectrum of LNnT (H_2O:D_2O 9:1 v/v solvent at pH 3.0) 13

Figure S18. ^{1}H -^{15}N HSQC spectrum of LNnT (H_2O:D_2O 9:1 v/v solvent at pH 3.0) 14

Figure S19. ^{1}H -^{15}N HSQC-TOCSY spectrum of LNnT (H_2O:D_2O 9:1 v/v solvent at pH 3.0) 14

Figure S20. ^{1}H NMR spectrum of 3'SL sodium salt (H_2O:D_2O 9:1 v/v solvent at pH 3.0) 15

Figure S21. ^{13}C NMR spectrum of 3'SL sodium salt (H_2O:D_2O 9:1 v/v solvent at pH 3.0) 15

Figure S22. ^{1}H -^{1}H COSY spectrum of 3'SL sodium salt (H_2O:D_2O 9:1 v/v solvent at pH 3.0) 16

Figure S23. ^{1}H -^{13}C HSQC spectrum of 3'SL sodium salt (H_2O:D_2O 9:1 v/v solvent at pH 3.0) 16

Figure S24. ^{1}H -^{13}C HMBC spectrum of 3'SL sodium salt (H_2O:D_2O 9:1 v/v solvent at pH 3.0) 17

Figure S25. ^{1}H -^{1}H TOCSY spectrum of 3'SL sodium salt (H_2O:D_2O 9:1 v/v solvent at pH 3.0) 17

Figure S26. ^{1}H -^{1}H ROESY spectrum of 3'SL sodium salt (H_2O:D_2O 9:1 v/v solvent at pH 3.0) 18

Figure S27. ^{1}H -^{15}N HSQC spectrum of 3'SL sodium salt (H_2O:D_2O 9:1 v/v solvent at pH 3.0) 18

Figure S28. ^{1}H -^{15}N HSQC-TOCSY spectrum of 3’SL sodium salt (H_2O:D_2O 9:1 v/v solvent at pH 3.0)........ 19
Figure S29. ^{1}H NMR spectrum of 6’SL sodium salt (H_2O:D_2O 9:1 v/v solvent at pH 3.0).... 20
Figure S30. ^{13}C NMR spectrum of 6’SL sodium salt (H_2O:D_2O 9:1 v/v solvent at pH 3.0)... 20
Figure S31. ^{1}H -^{1}H COSY spectrum of 6’SL sodium salt (H_2O:D_2O 9:1 v/v solvent at pH 3.0) 21
Figure S32. ^{1}H -^{13}C HSQC spectrum of 6’SL sodium salt (H_2O:D_2O 9:1 v/v solvent at pH 3.0) 21
Figure S33. ^{1}H -^{13}C HMBC spectrum of 6’SL sodium salt (H_2O:D_2O 9:1 v/v solvent at pH 3.0) 22
Figure S34. ^{1}H -^{1}H TOCSY spectrum of 6’SL sodium salt (H_2O:D_2O 9:1 v/v solvent at pH 3.0) 22
Figure S35. ^{1}H -^{1}H ROESY spectrum of 6’SL sodium salt (H_2O:D_2O 9:1 v/v solvent at pH 3.0) 23
Figure S36. ^{1}H -^{15}N HSQC spectrum of 6’SL sodium salt (H_2O:D_2O 9:1 v/v solvent at pH 3.0) 23
Figure S37. H -^{15}N HSQC-TOCSY spectrum of 6’SL sodium salt (H_2O:D_2O 9:1 v/v solvent at pH 3.0)........ 24
Figure S38. ^{1}H NMR spectrum of LNFP II (H_2O:D_2O 9:1 v/v solvent at pH 3.0)........ 25
Figure S39. ^{13}C NMR spectrum of LNFP II (H_2O:D_2O 9:1 v/v solvent at pH 3.0)........ 25
Figure S40. ^{1}H -^{1}H COSY spectrum of LNFP II (H_2O:D_2O 9:1 v/v solvent at pH 3.0)........ 26
Figure S41. ^{1}H -^{13}C HSQC spectrum of LNFP II (H_2O:D_2O 9:1 v/v solvent at pH 3.0)........ 26
Figure S42. ^{1}H -^{13}C HMBC spectrum of LNFP II (H_2O:D_2O 9:1 v/v solvent at pH 3.0)........ 27
Figure S43. ^{1}H -^{1}H TOCSY spectrum of LNFP II (H_2O:D_2O 9:1 v/v solvent at pH 3.0) 27
Figure S44. ^{1}H -^{1}H ROESY spectrum of LNFP II (H_2O:D_2O 9:1 v/v solvent at pH 3.0) 28
Figure S45. ^{1}H -^{15}N HSQC spectrum of LNFP II (H_2O:D_2O 9:1 v/v solvent at pH 3.0) 28
Figure S46. ^{1}H -^{15}N HSQC-TOCSY spectrum of LNFP II (H_2O:D_2O 9:1 v/v solvent at pH 3.0) 29
Figure S47. ^{1}H NMR spectrum of LNFP III (H_2O:D_2O 9:1 v/v solvent at pH 3.0)........ 30
Figure S48. ^{13}C NMR spectrum of LNFP III (H_2O:D_2O 9:1 v/v solvent at pH 3.0) 30
Figure S49. ^{1}H -^{1}H COSY spectrum of LNFP III (H_2O:D_2O 9:1 v/v solvent at pH 3.0) 31
Figure S50. ^{1}H -^{13}C HSQC spectrum of LNFP III (H_2O:D_2O 9:1 v/v solvent at pH 3.0) 31
Figure S51. ^{1}H -^{13}C HMBC spectrum of LNFP III (H_2O:D_2O 9:1 v/v solvent at pH 3.0)........ 32
Figure S52. ^{1}H -^{1}H TOCSY spectrum of LNFP III (H_2O:D_2O 9:1 v/v solvent at pH 3.0)........ 32
Figure S53. ^{1}H -^{1}H ROESY spectrum of LNFP III (H_2O:D_2O 9:1 v/v solvent at pH 3.0)........ 33
Figure S54. ^{1}H -^{15}N HSQC spectrum of LNFP III (H_2O:D_2O 9:1 v/v solvent at pH 3.0)........ 33
Figure S55. ^{1}H -^{15}N HSQC-TOCSY spectrum of LNFP III (H_2O:D_2O 9:1 v/v solvent at pH 3.0) 34

Figure S56. ^{1}H NMR spectrum of LSTa (H_2O:D_2O 9:1 v/v solvent at pH 3.0)....................... 35
Figure S57. ^{13}C NMR spectrum of LSTa (H_2O:D_2O 9:1 v/v solvent at pH 3.0) 35
Figure S58. ^{1}H -^{1}H COSY spectrum of LSTa (H_2O:D_2O 9:1 v/v solvent at pH 3.0) 36
Figure S59. ^{1}H -^{13}C HSQC spectrum of LSTa (H_2O:D_2O 9:1 v/v solvent at pH 3.0) 36
Figure S60. ^{1}H -^{13}C HMBC spectrum of LSTa (H_2O:D_2O 9:1 v/v solvent at pH 3.0)............ 37
Figure S61. ^{1}H -^{1}H TOCSY spectrum of LSTa (H_2O:D_2O 9:1 v/v solvent at pH 3.0)............ 37
Figure S62. ^{1}H -^{1}H ROESY spectrum of LSTa (H_2O:D_2O 9:1 v/v solvent at pH 3.0)............ 38
Figure S63. ^{1}H -^{15}N HSQC spectrum of LSTa (H_2O:D_2O 9:1 v/v solvent at pH 3.0)............. 39
Figure S64. ^{1}H -^{15}N HSQC-TOCSY spectrum of LSTa (H_2O:D_2O 9:1 v/v solvent at pH 3.0) 39
Figure S65. ^{1}H NMR spectrum of LSTb (H_2O:D_2O 9:1 v/v solvent at pH 3.0) 40
Figure S66. ^{13}C NMR spectrum of LSTb (H_2O:D_2O 9:1 v/v solvent at pH 3.0) 40
Figure S67. 1H -1H COSY spectrum of LSTb (H_2O:D_2O 9:1 v/v solvent at pH 3.0)............. 41
Figure S68. ^{1}H -^{13}C HSQC spectrum of LSTb (H_2O:D_2O 9:1 v/v solvent at pH 3.0)............. 41
Figure S69. ^{1}H -^{13}C HMBC spectrum of LSTb (H_2O:D_2O 9:1 v/v solvent at pH 3.0)............ 42
Figure S70. ^{1}H -^{1}H TOCSY spectrum of LSTb (H_2O:D_2O 9:1 v/v solvent at pH 3.0)............ 42
Figure S71. ^{1}H -^{1}H ROESY spectrum of LSTb (H_2O:D_2O 9:1 v/v solvent at pH 3.0)............ 43
Figure S72. ^{1}H -^{15}N HSQC spectrum of LSTb (H_2O:D_2O 9:1 v/v solvent at pH 3.0)............. 43
Figure S73. ^{1}H -^{15}N HSQC-TOCSY spectrum of LSTb (H_2O:D_2O 9:1 v/v solvent at pH 3.0) 44
Figure S74. Overlaid ^{1}H-^{15}N HSQC-TOCSY spectra of 3'SL and 6'SL with the ^{1}H NMR assignment of their Neu5Ac moiety... 45
Figure S75. Overlaid ^{1}H-^{15}N HSQC-TOCSY spectra of LNFP II and LNFP III with the ^{1}H NMR assignment of their GlcNAc moiety.. 45
Figure S76. ^{1}H-^{13}C HSQC spectrum of LAcNAc 46
Figure S77. ^{1}H-^{13}C HSQC spectrum of LNB... 46
Table S1. Complete resonance assignment of the disaccharides LacNAc and LNB............... 47
Figure S78. Overlaid ^{1}H-^{15}N HSQC spectra of LNnT and para-LNnH.................................. 47
Figure S79. Full ^{1}H-^{15}N HSQC-TOCSY spectrum of LNT and LNnT mixture (1:1, H2O:D2O 9:1 v/v solvent at pH 3.0).. 48
Figure S80. Overlaid full ^{1}H-^{15}N HSQC-TOCSY spectra of LSTa and LSTb. 49
Figure S81. ^{1}H -^{15}N HSQC spectrum of *N*,*N*',*N*'',*N*'''-Tetraacetylchitotetraose (H_2O:D_2O 9:1 v/v solvent at pH 3.0) 49
Figure S82. ^{1}H -^{15}N HSQC-TOCSY spectrum of *N*,*N*',*N*'',*N*'''-Tetraacetylchitotetraose (H_2O:D_2O 9:1 v/v solvent at pH 3.0) 50

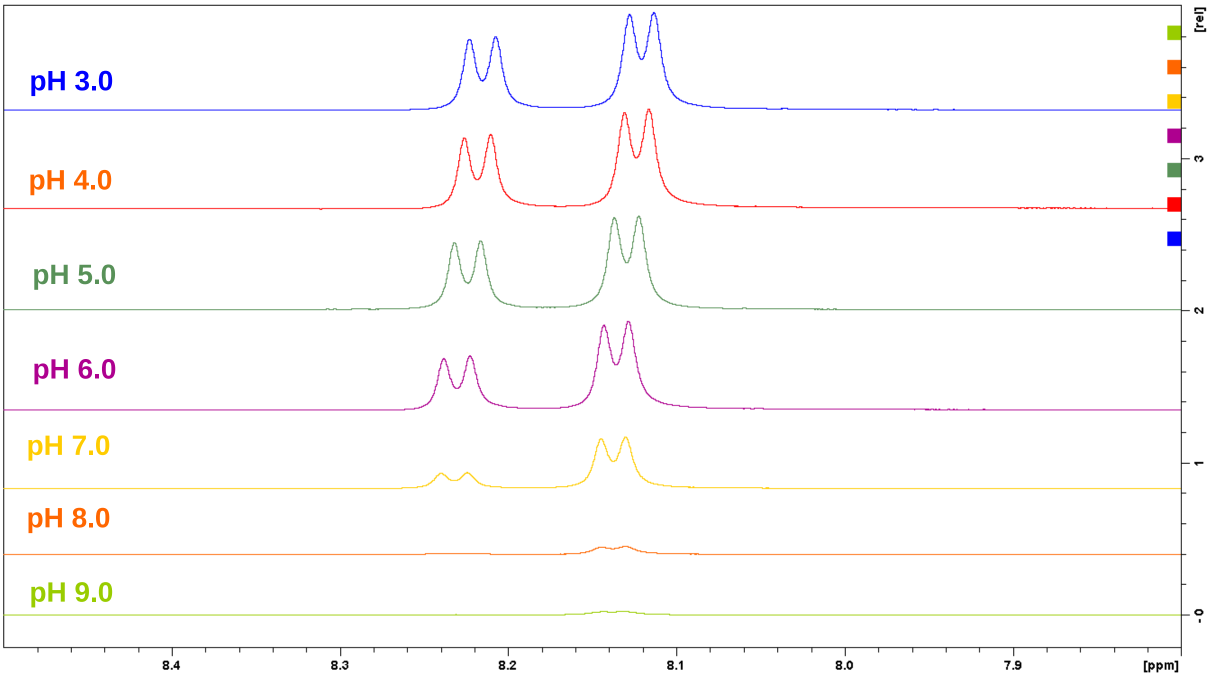

Figure S1. Selected regions of the ^{1}H NMR spectra of N-acetylglucosamine (GlcNAc) standard in H_2O:D_2O 9:1 v/v unbuffered solution, in the range of pH 3.0-9.0. Anomeric NH proton resonances of GlcNAc: β = 8.22 ppm, α = 8.12 ppm.

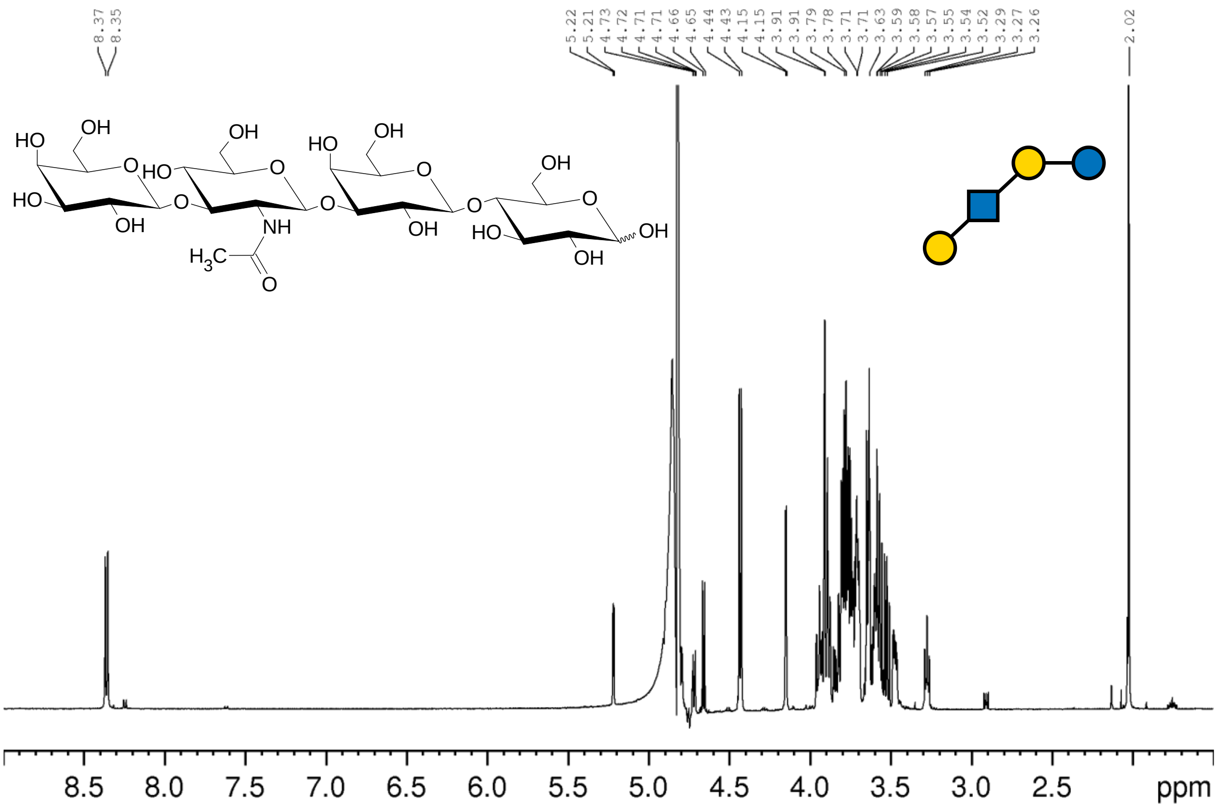

Figure S2. ^{1}H NMR spectrum of LNT (H_2O:D_2O 9:1 v/v solvent at pH 3.0)

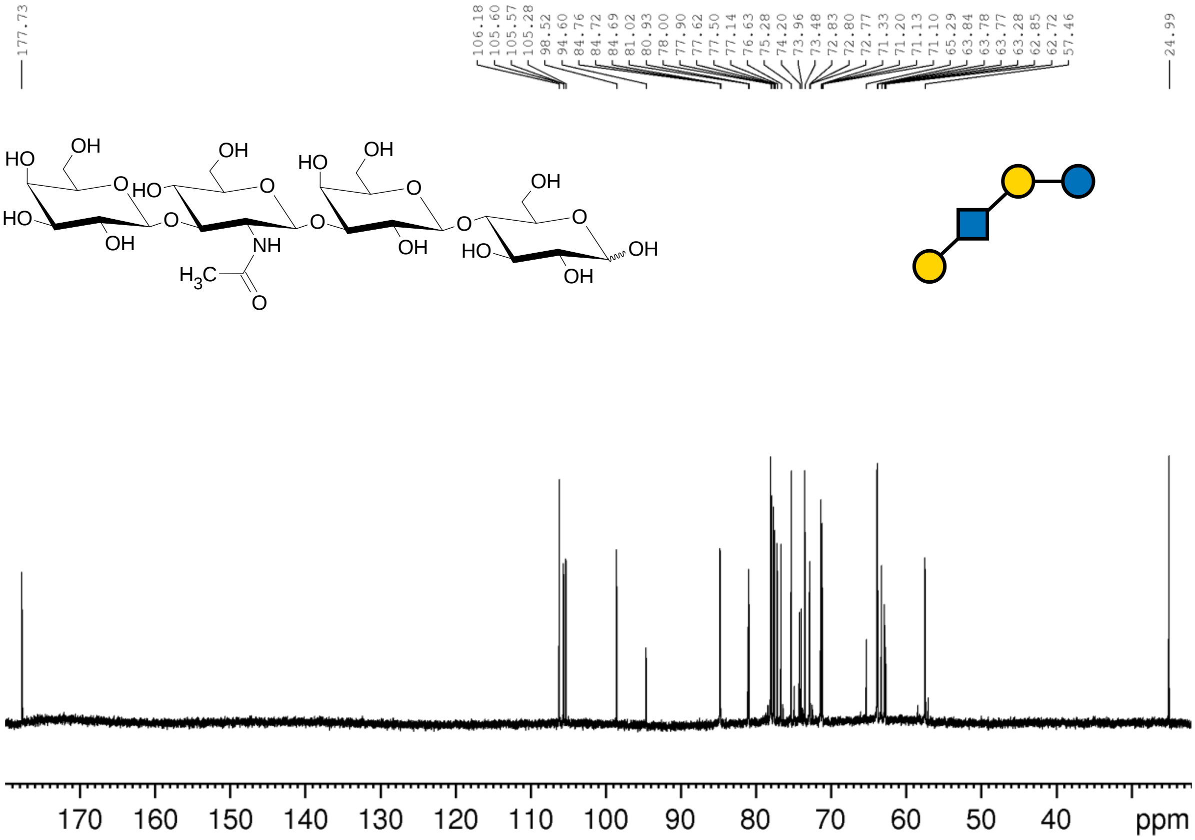

Figure S3. ^{13}C NMR spectrum of LNT (H_2O:D_2O 9:1 v/v solvent at pH 3.0)

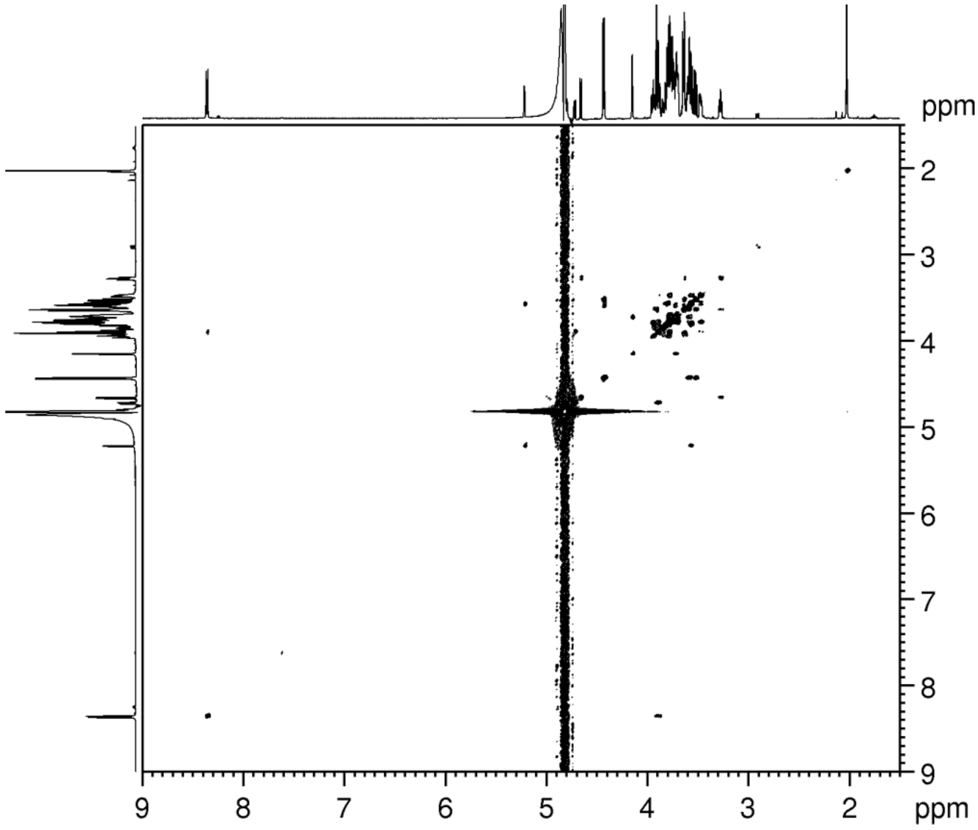

Figure S4. ^{1}H -^{1}H COSY spectrum of LNT (H_2O:D_2O 9:1 v/v solvent at pH 3.0)

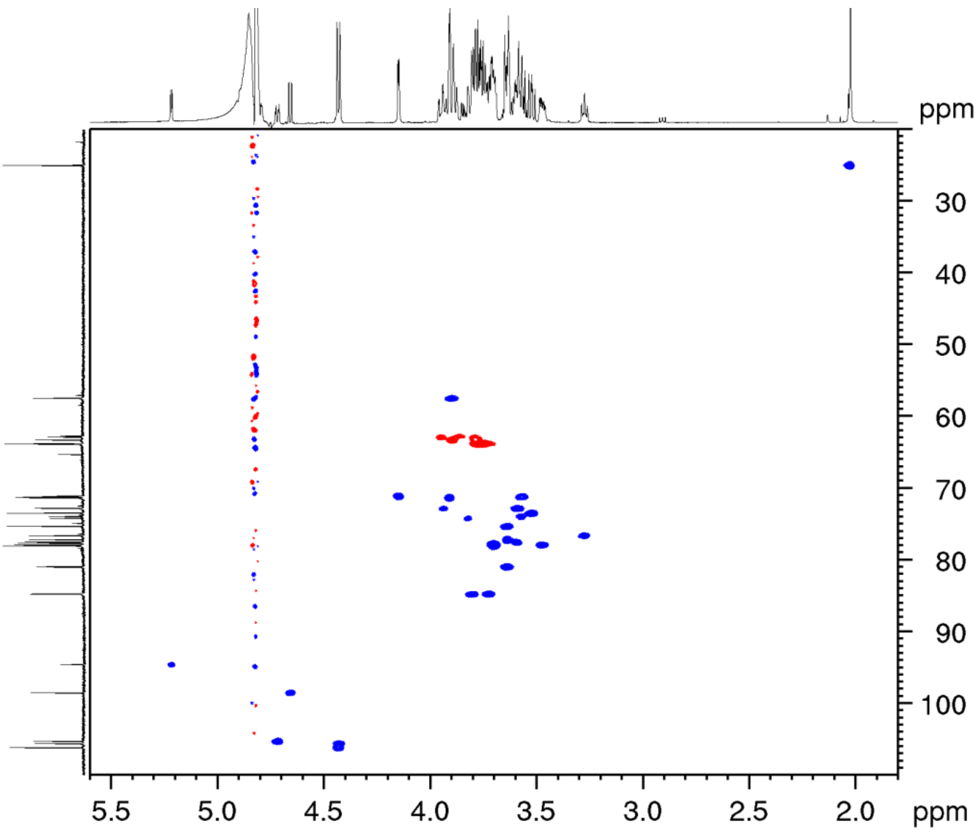

Figure S5. ^{1}H -^{13}C HSQC spectrum of LNT (H_2O:D_2O 9:1 v/v solvent at pH 3.0)

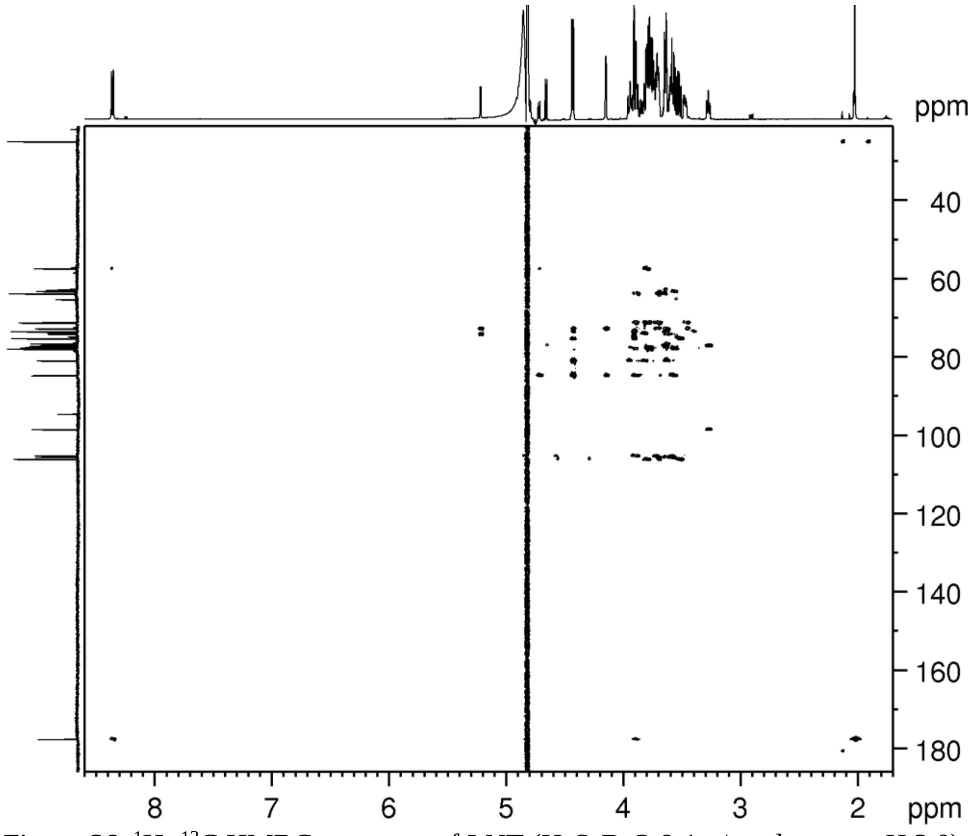

Figure S6. ^{1}H -^{13}C HMBC spectrum of LNT (H_2O:D_2O 9:1 v/v solvent at pH 3.0)

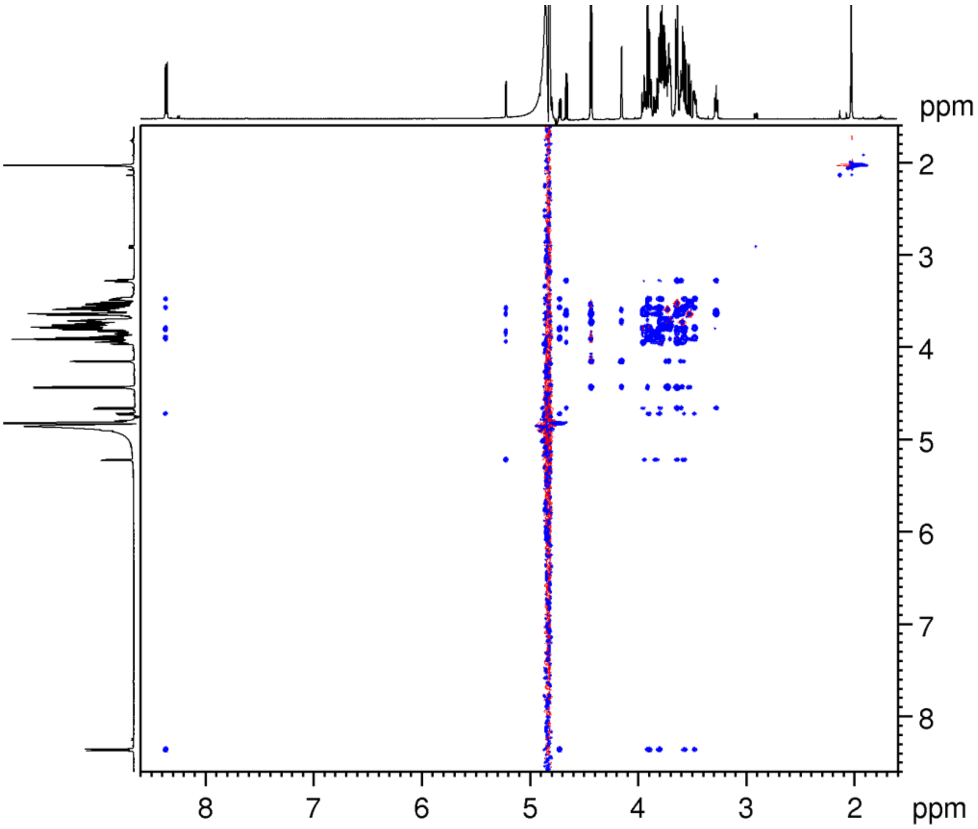

Figure S7. ^{1}H -^{1}H TOCSY spectrum of LNT (H_2O:D_2O 9:1 v/v solvent at pH 3.0)

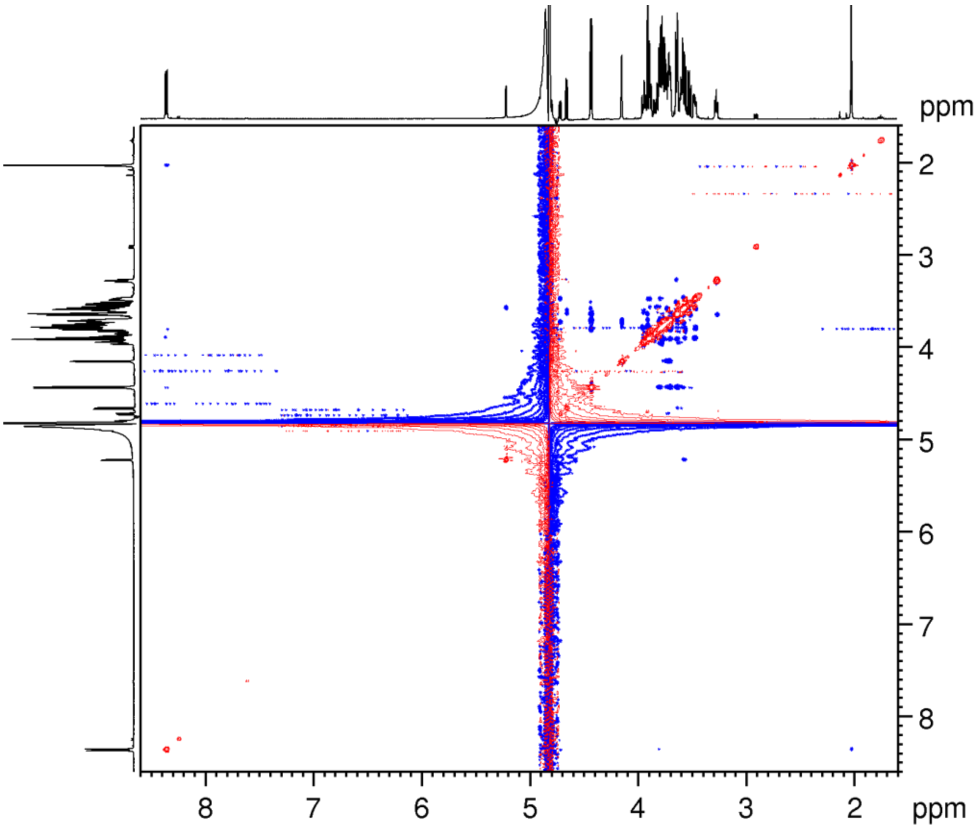

Figure S8. ^{1}H -^{1}H ROESY spectrum of LNT (H_2O:D_2O 9:1 v/v solvent at pH 3.0)

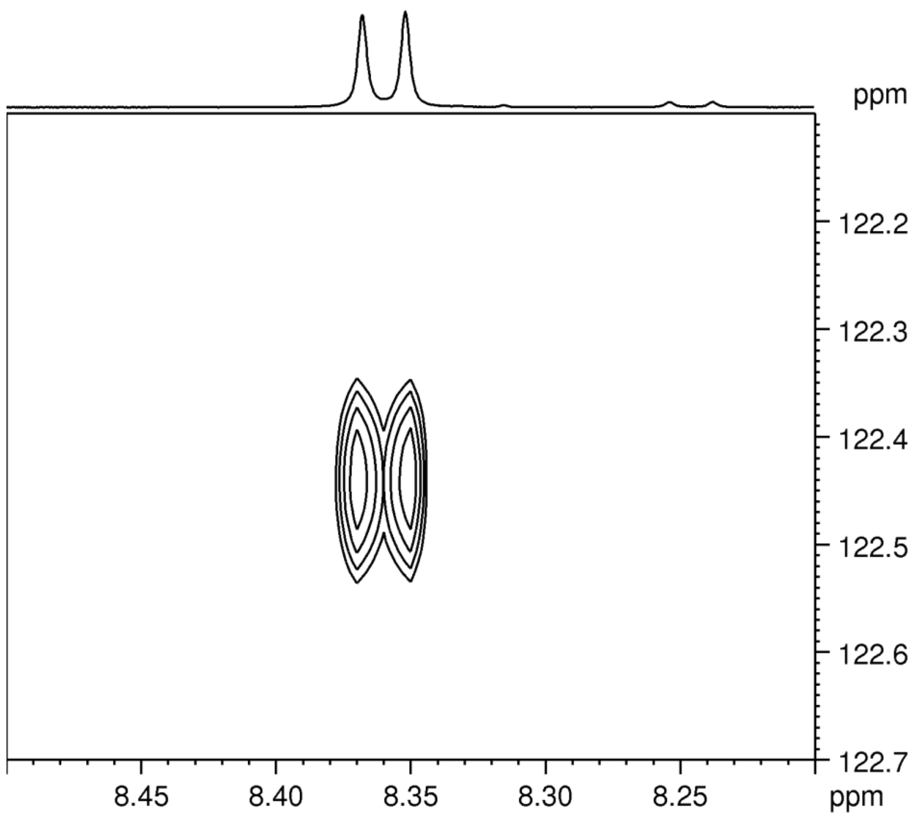

Figure S9. ^{1}H -^{15}N HSQC spectrum of LNT (H_2O:D_2O 9:1 v/v solvent at pH 3.0)

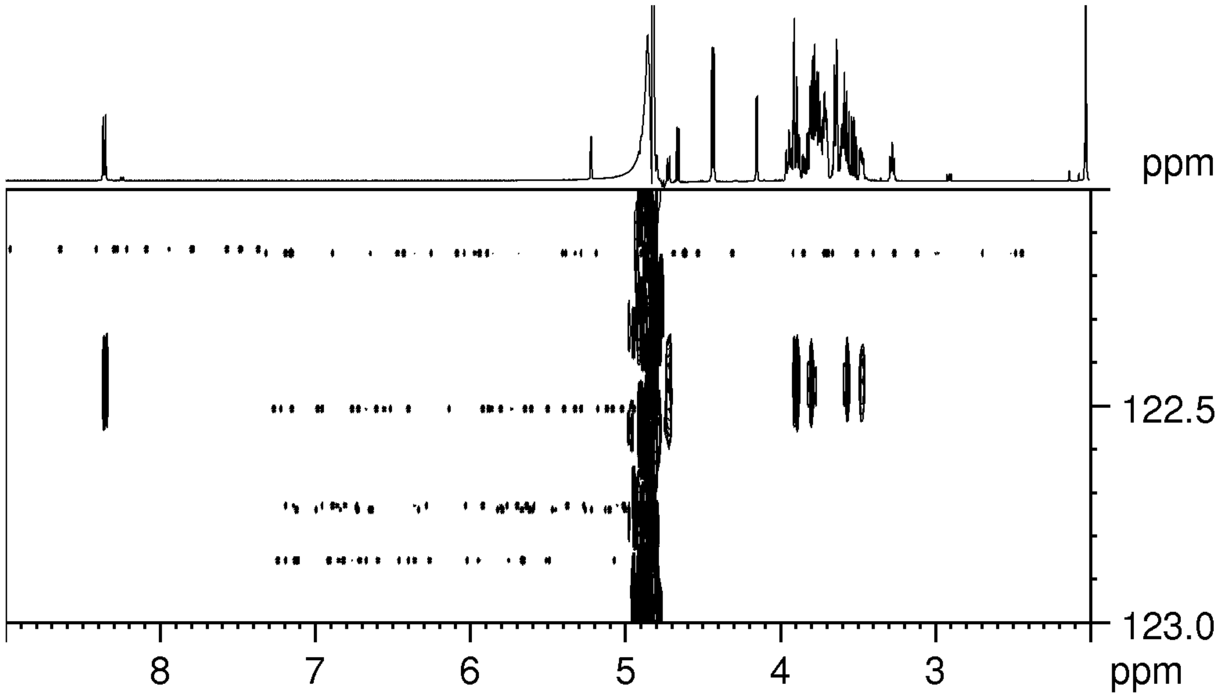

Figure S10. ^{1}H -^{15}N HSQC-TOCSY spectrum of LNT (H_2O:D_2O 9:1 v/v solvent at pH 3.0)

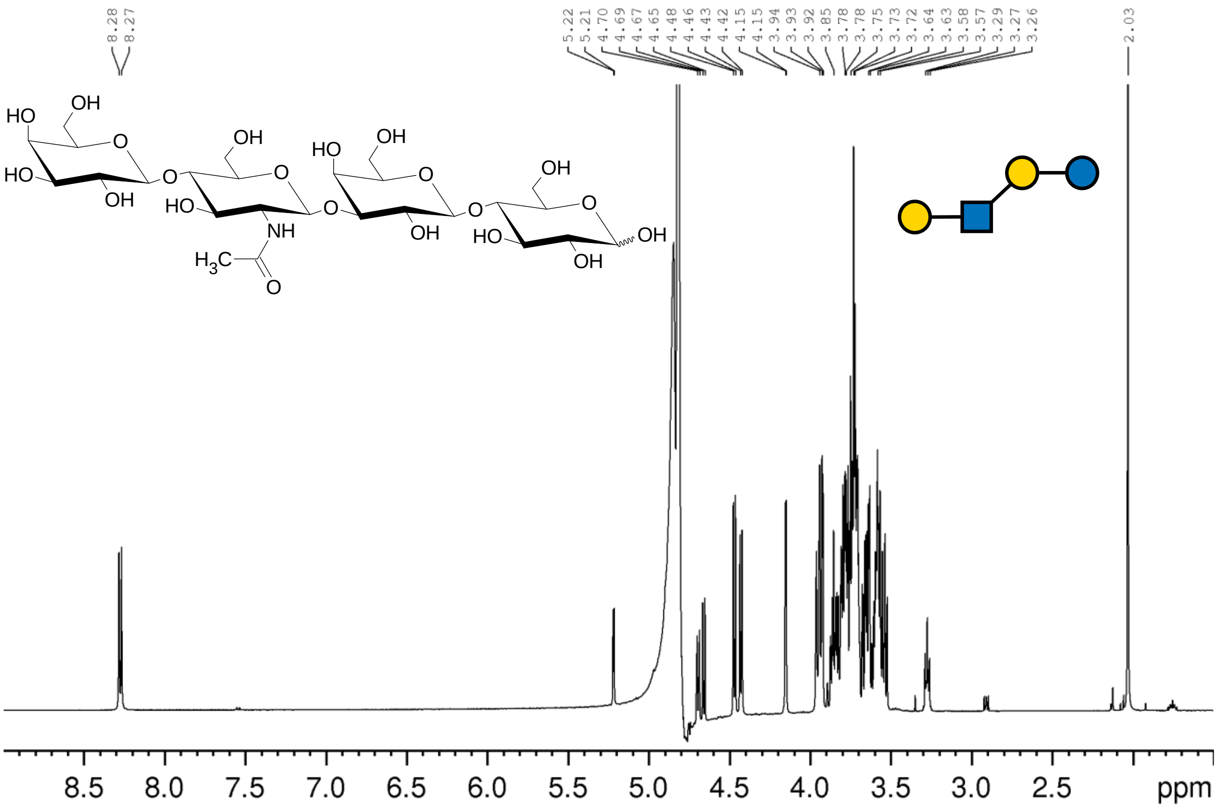

Figure S11. ^{1}H NMR spectrum of LNnT (H_2O:D_2O 9:1 v/v solvent at pH 3.0)

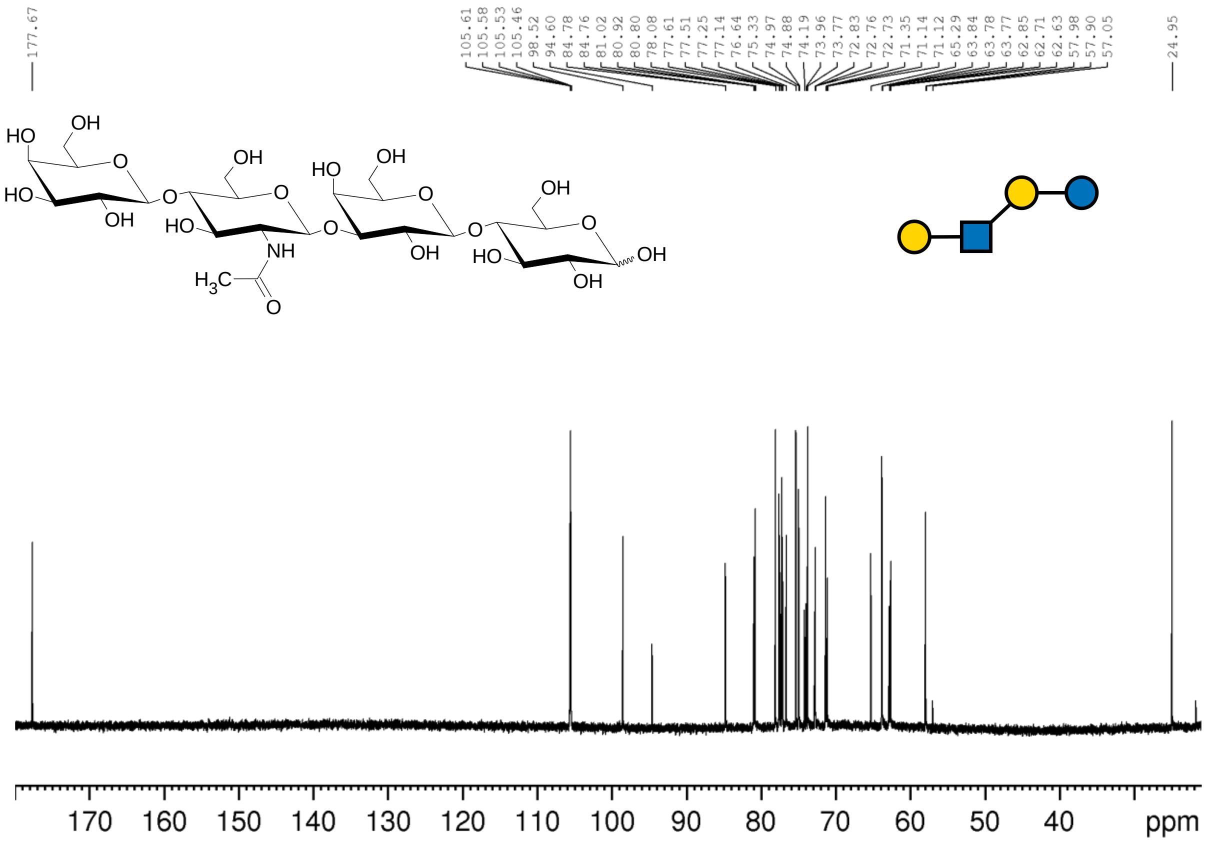

Figure S12. ^{13}C NMR spectrum of LNnT (H_2O:D_2O 9:1 v/v solvent at pH 3.0)

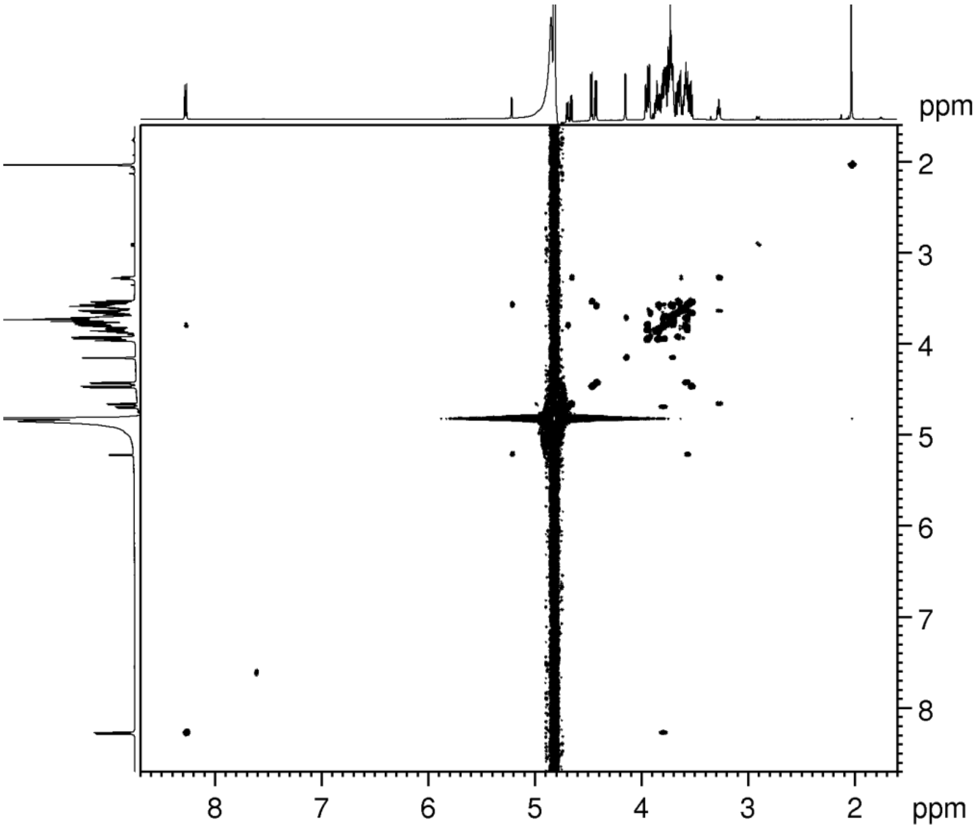

Figure S13. ^{1}H -^{1}H COSY spectrum of LNnT (H_2O:D_2O 9:1 v/v solvent at pH 3.0)

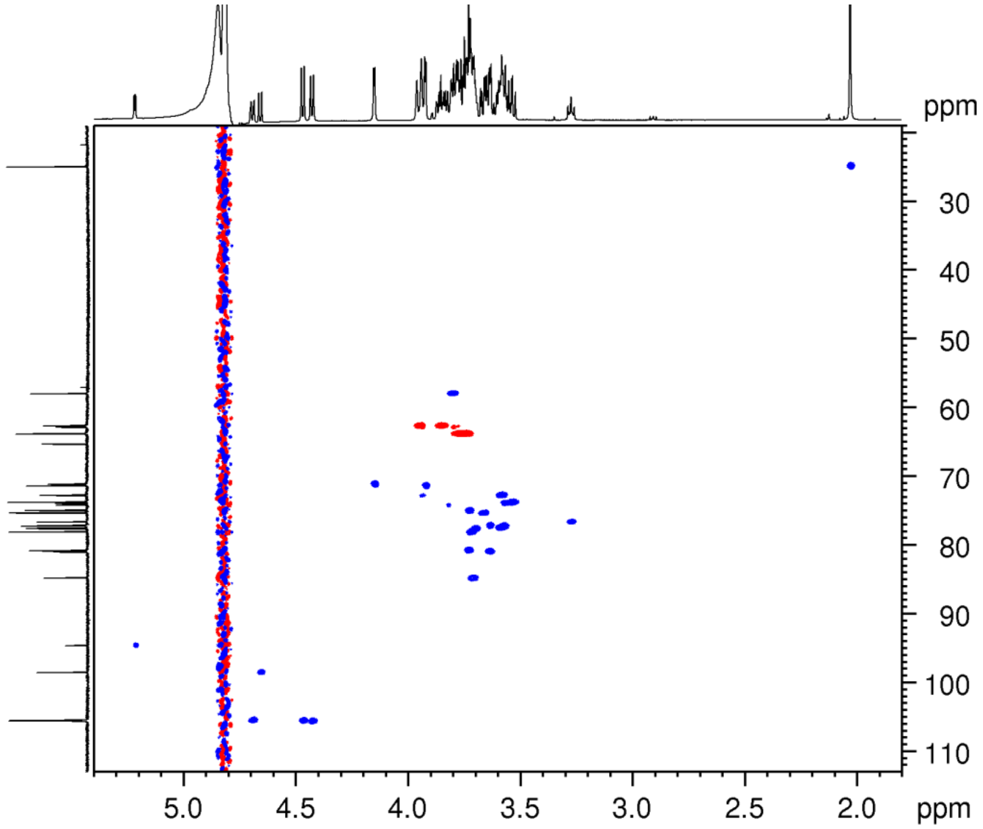

Figure S14. ^{1}H -^{13}C HSQC spectrum of LNnT (H_2O:D_2O 9:1 v/v solvent at pH 3.0)

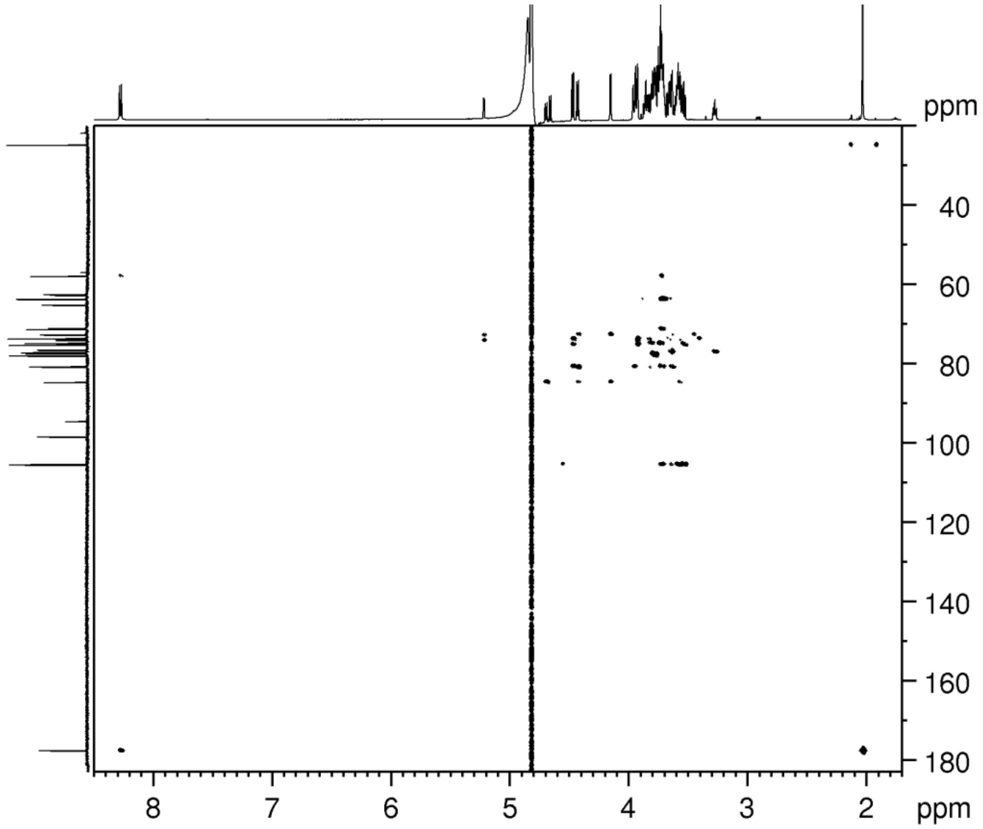

Figure S15. ^{1}H -^{13}C HMBC spectrum of LNnT (H_2O:D_2O 9:1 v/v solvent at pH 3.0)

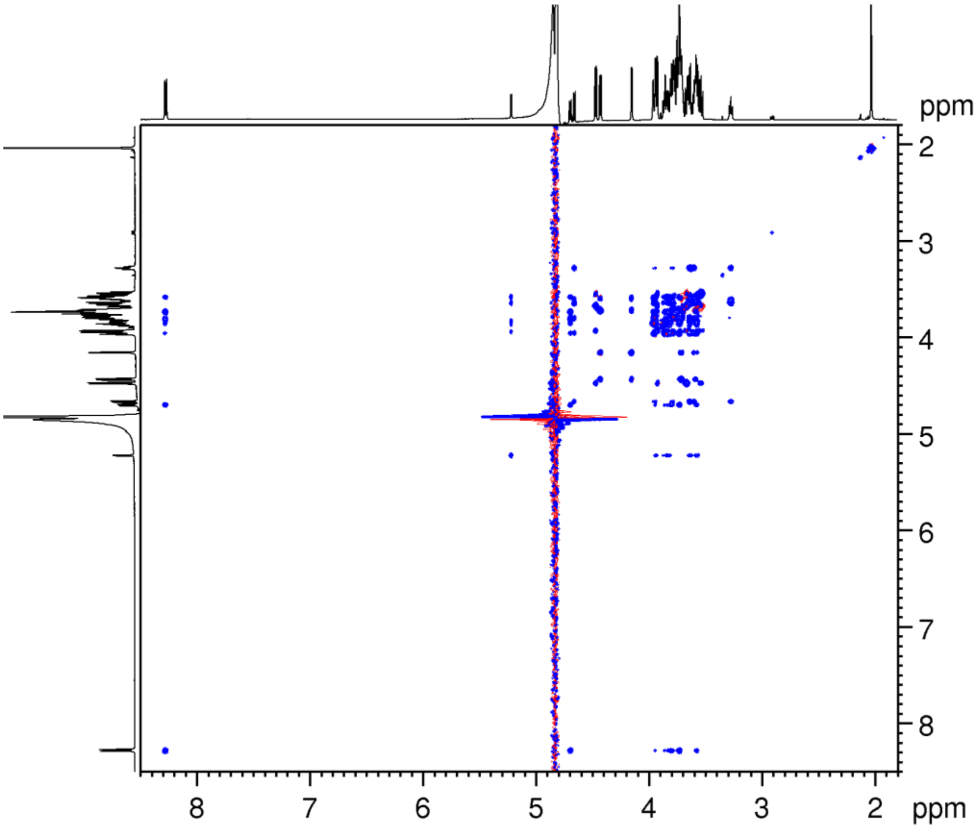

Figure S16. ^{1}H -^{1}H TOCSY spectrum of LNnT (H_2O:D_2O 9:1 v/v solvent at pH 3.0)

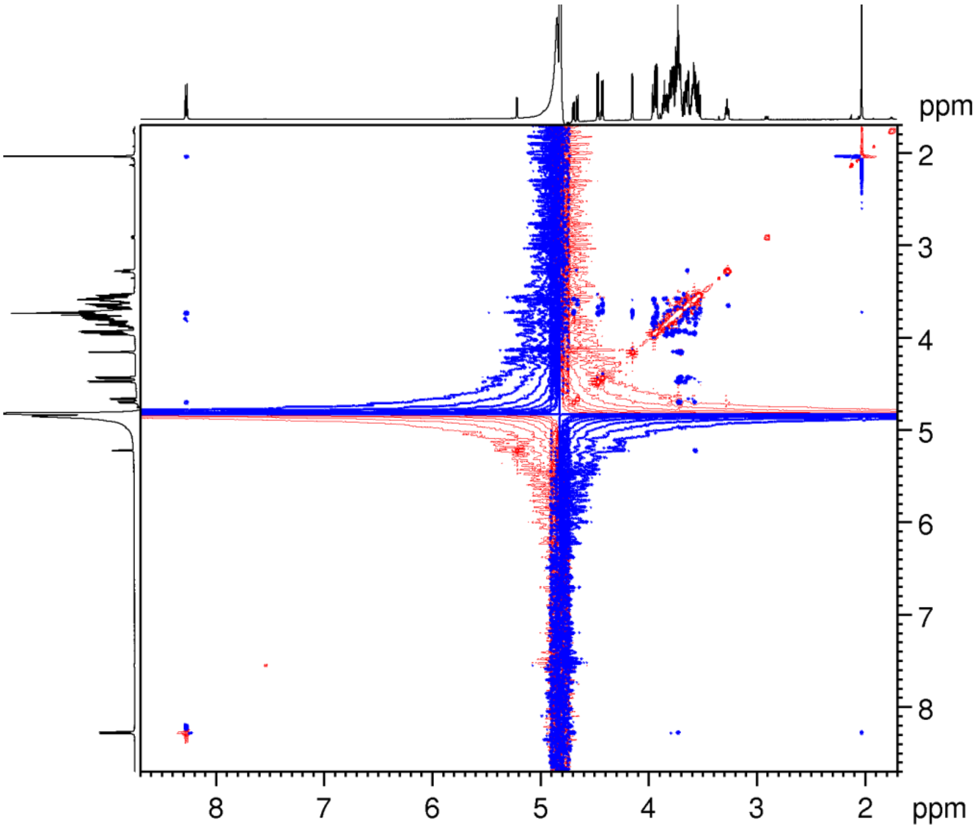

Figure S17. ^{1}H -^{1}H ROESY spectrum of LNnT (H_2O:D_2O 9:1 v/v solvent at pH 3.0)

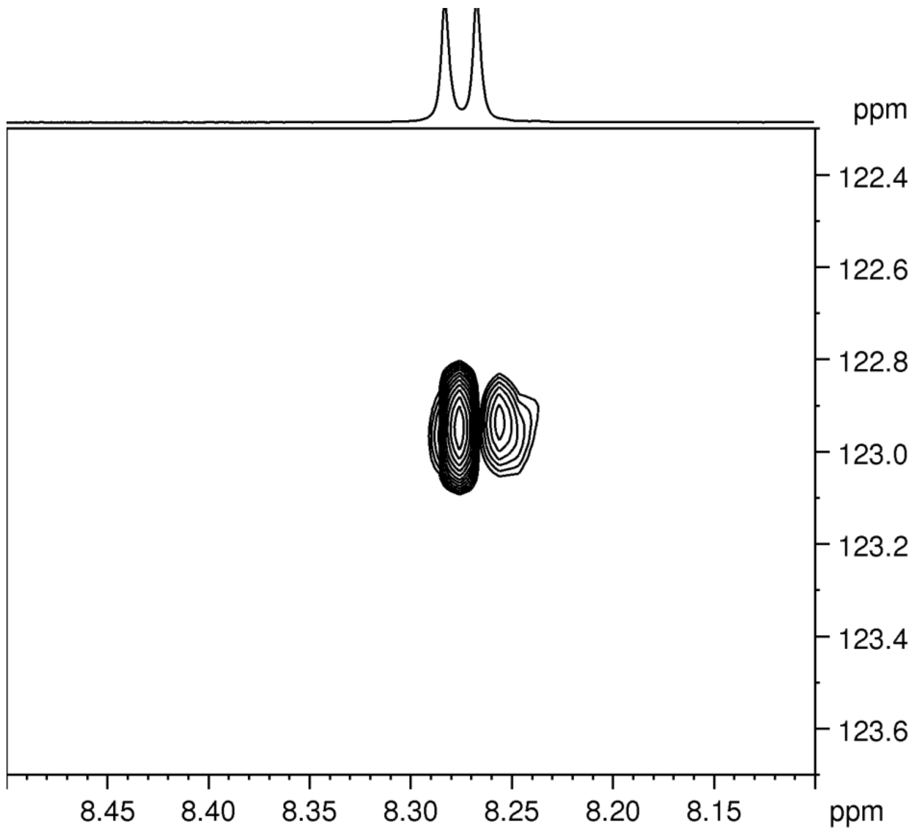

Figure S18. ^{1}H -^{15}N HSQC spectrum of LNnT (H_2O:D_2O 9:1 v/v solvent at pH 3.0)

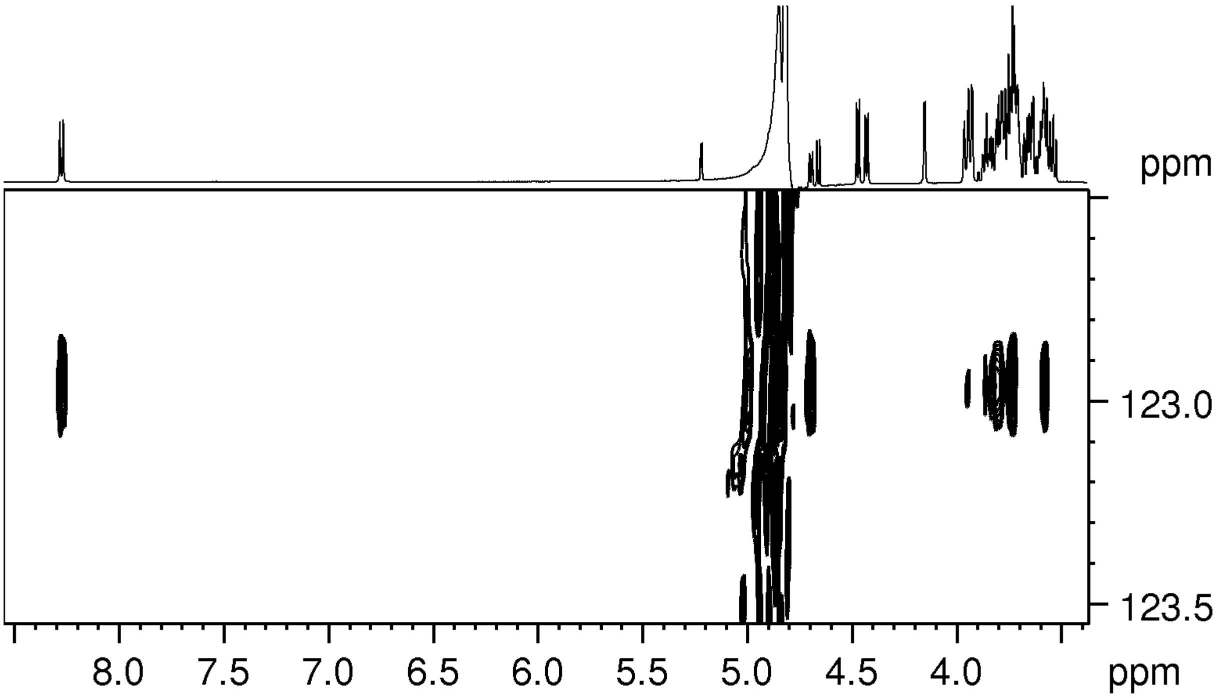

Figure S19. ^{1}H -^{15}N HSQC-TOCSY spectrum of LNnT (H_2O:D_2O 9:1 v/v solvent at pH 3.0)

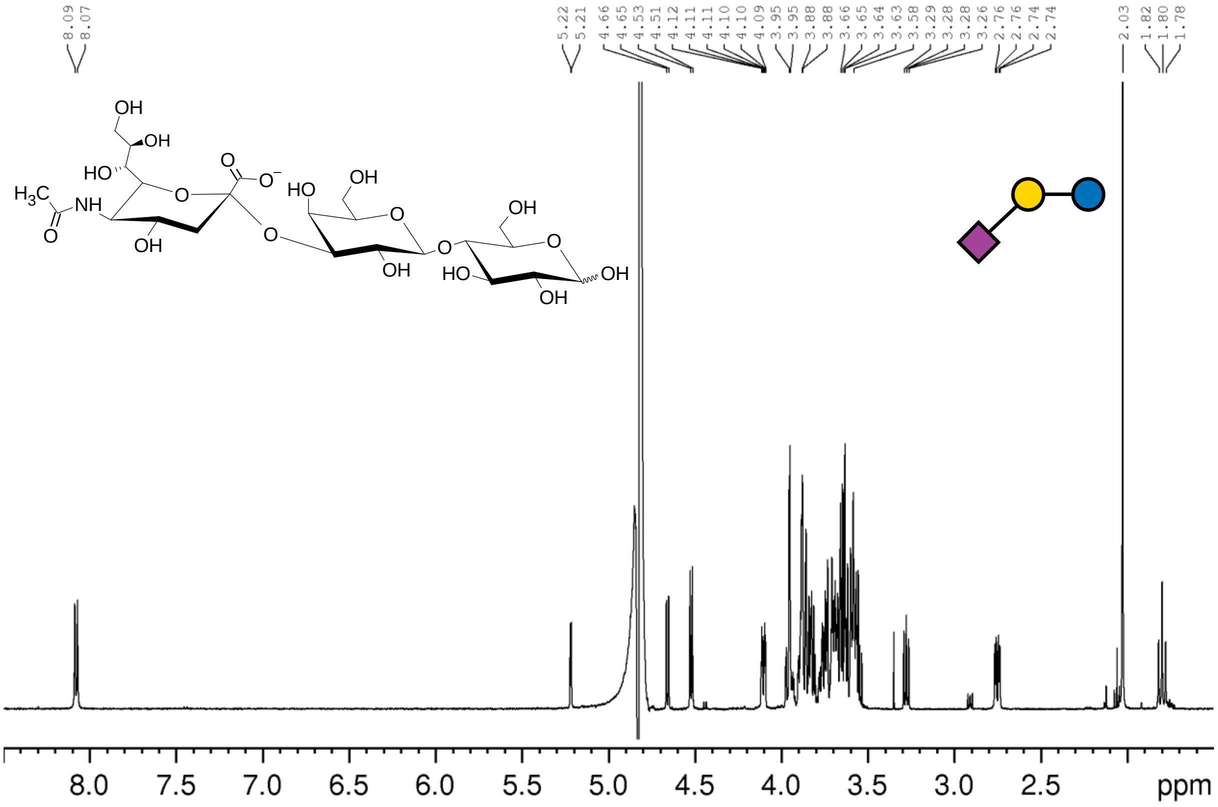

Figure S20. ^{1}H NMR spectrum of 3'SL sodium salt (H_2O:D_2O 9:1 v/v solvent at pH 3.0)

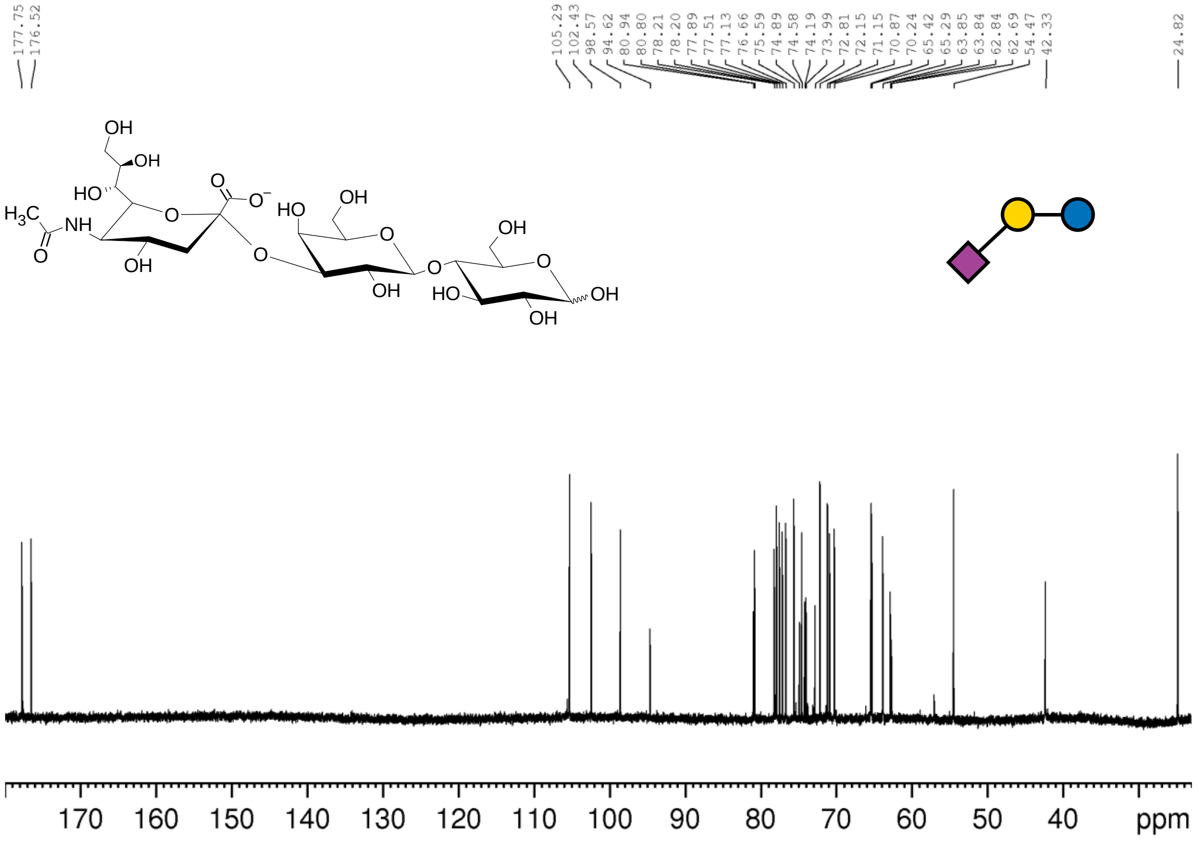

Figure S21. ^{13}C NMR spectrum of 3'SL sodium salt (H_2O:D_2O 9:1 v/v solvent at pH 3.0)

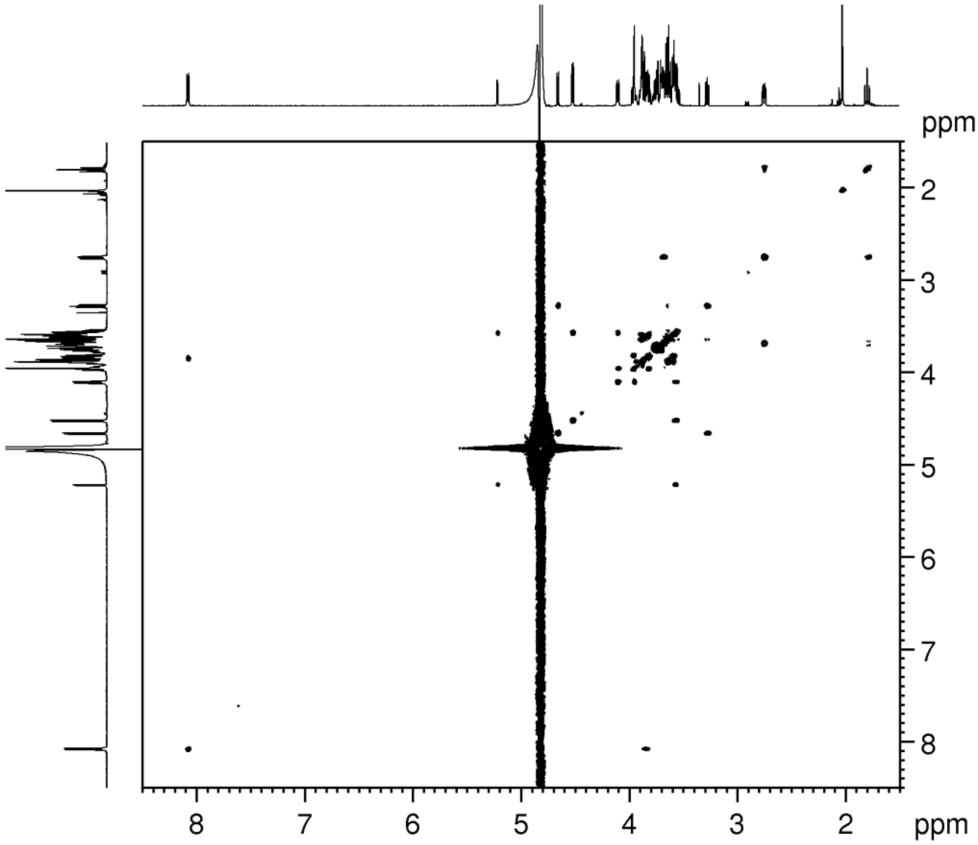

Figure S22. ^{1}H -^{1}H COSY spectrum of 3’SL sodium salt (H_2O:D_2O 9:1 v/v solvent at pH 3.0)

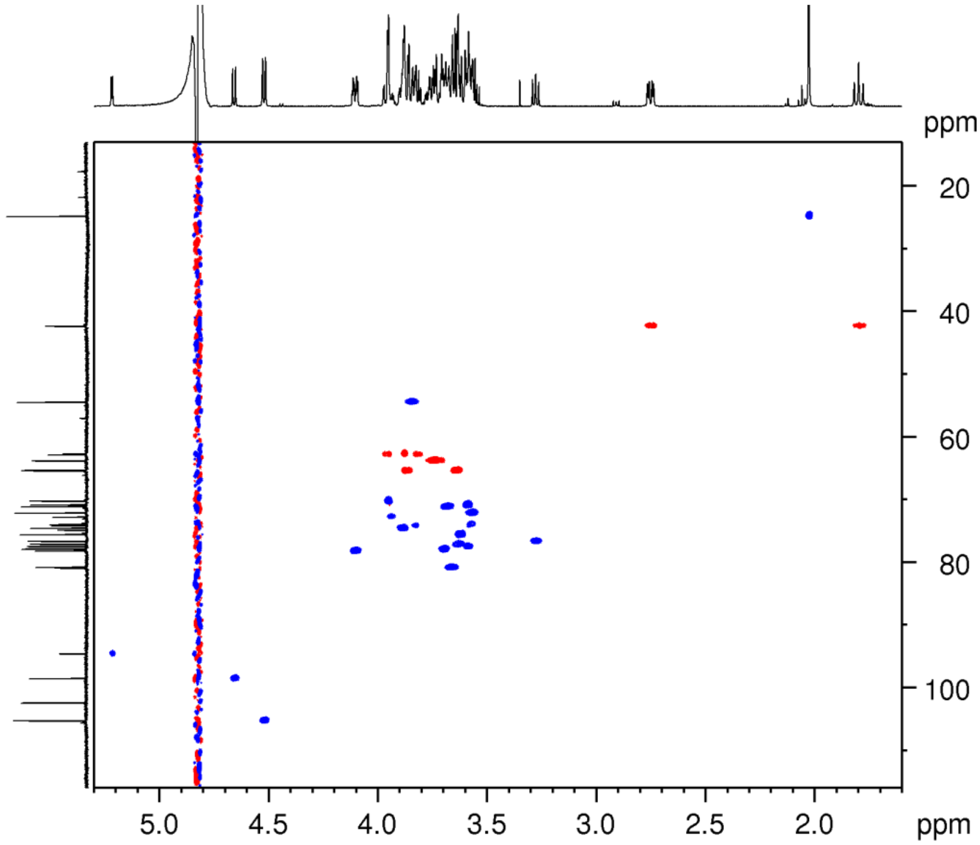

Figure S23. ^{1}H -^{13}C HSQC spectrum of 3’SL sodium salt (H_2O:D_2O 9:1 v/v solvent at pH 3.0)

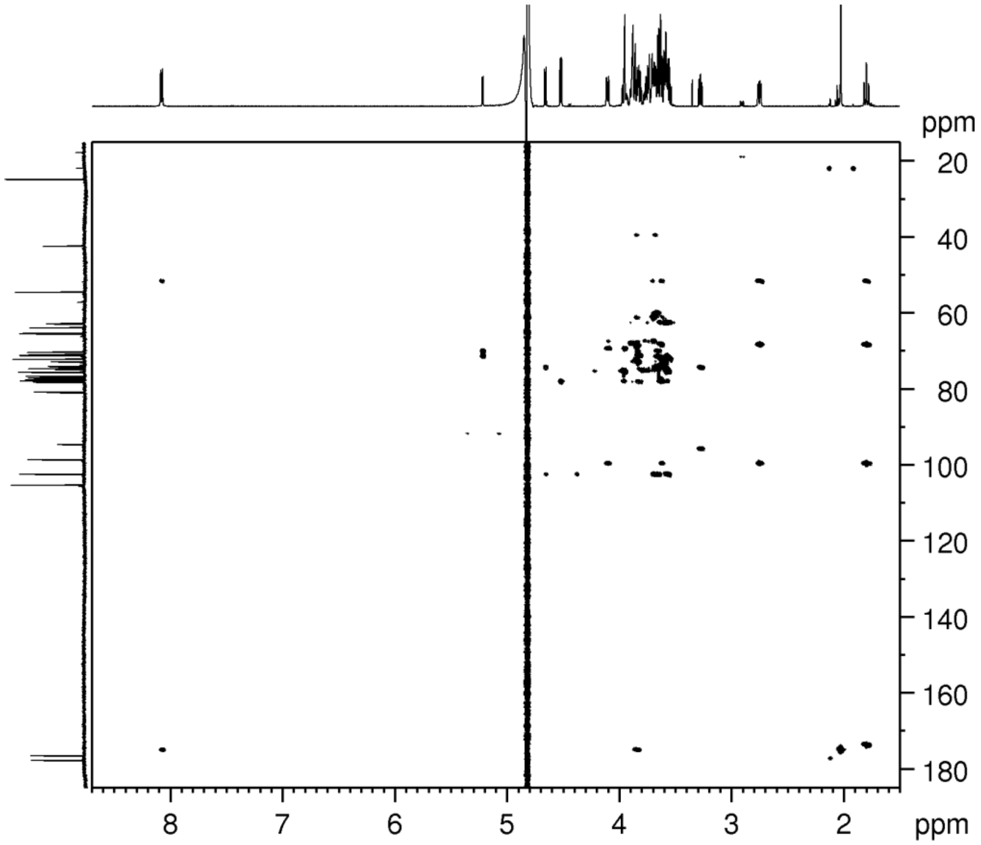

Figure S24. ^{1}H -^{13}C HMBC spectrum of 3’SL sodium salt (H_2O:D_2O 9:1 v/v solvent at pH 3.0)

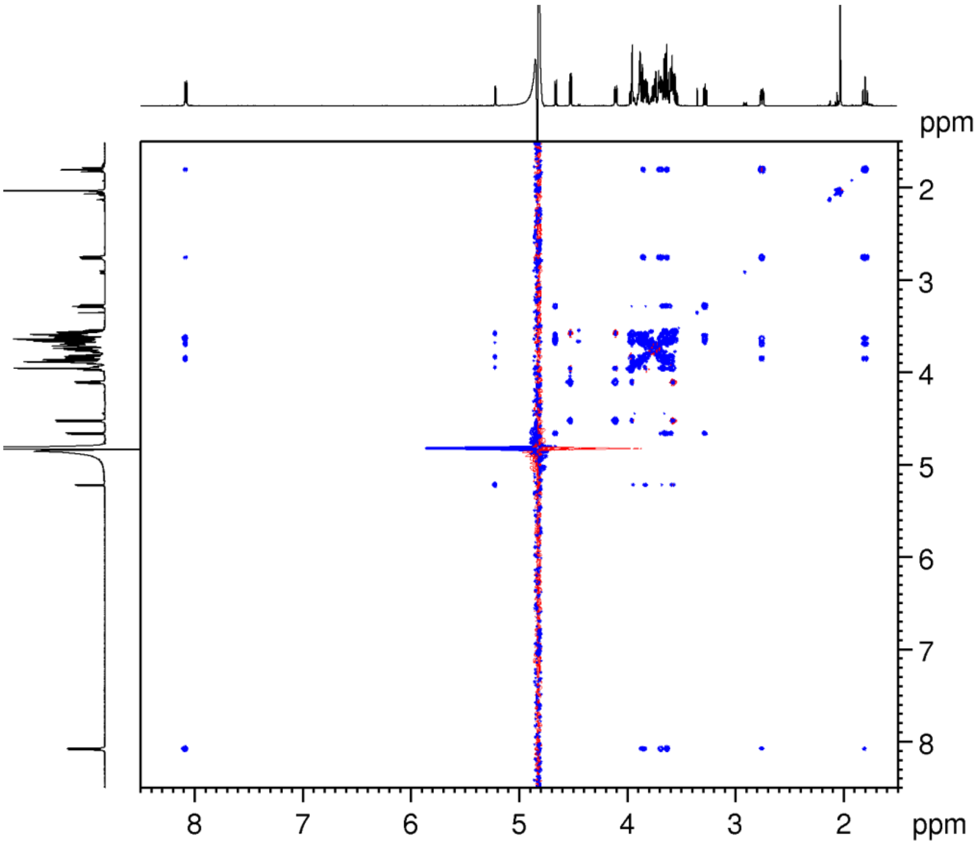

Figure S25. ^{1}H -^{1}H TOCSY spectrum of 3’SL sodium salt (H_2O:D_2O 9:1 v/v solvent at pH 3.0)

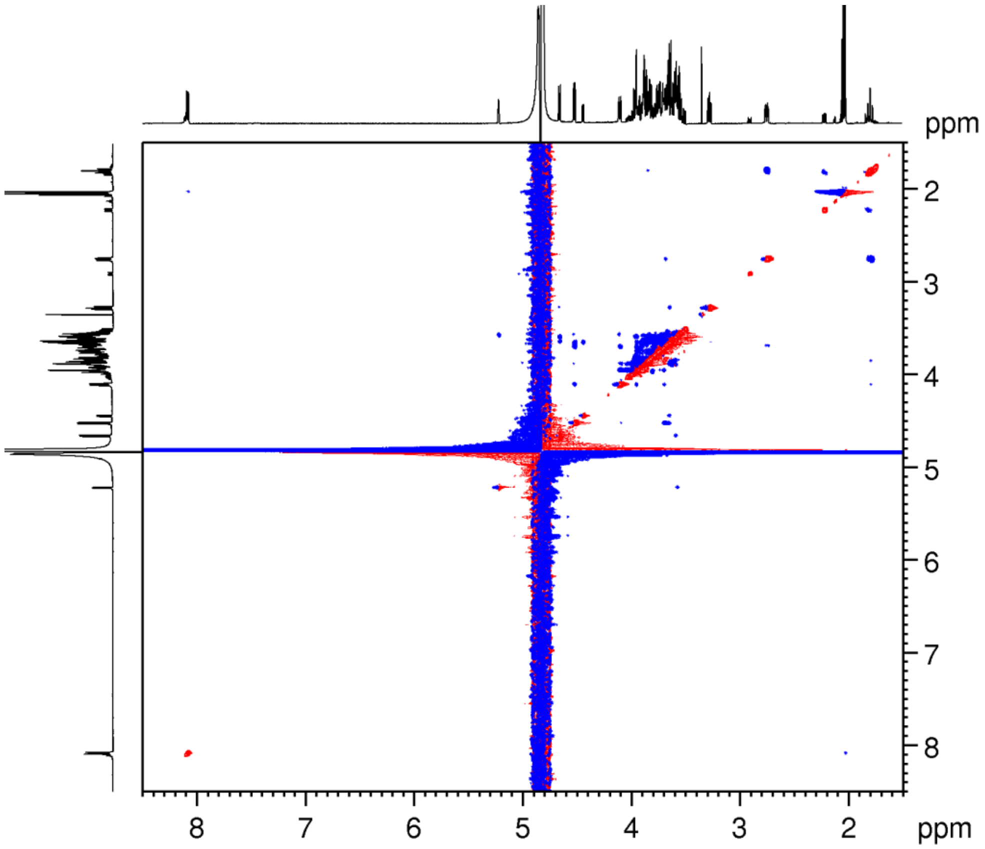

Figure S26. ^{1}H -^{1}H ROESY spectrum of 3'SL sodium salt (H_2O:D_2O 9:1 v/v solvent at pH 3.0)

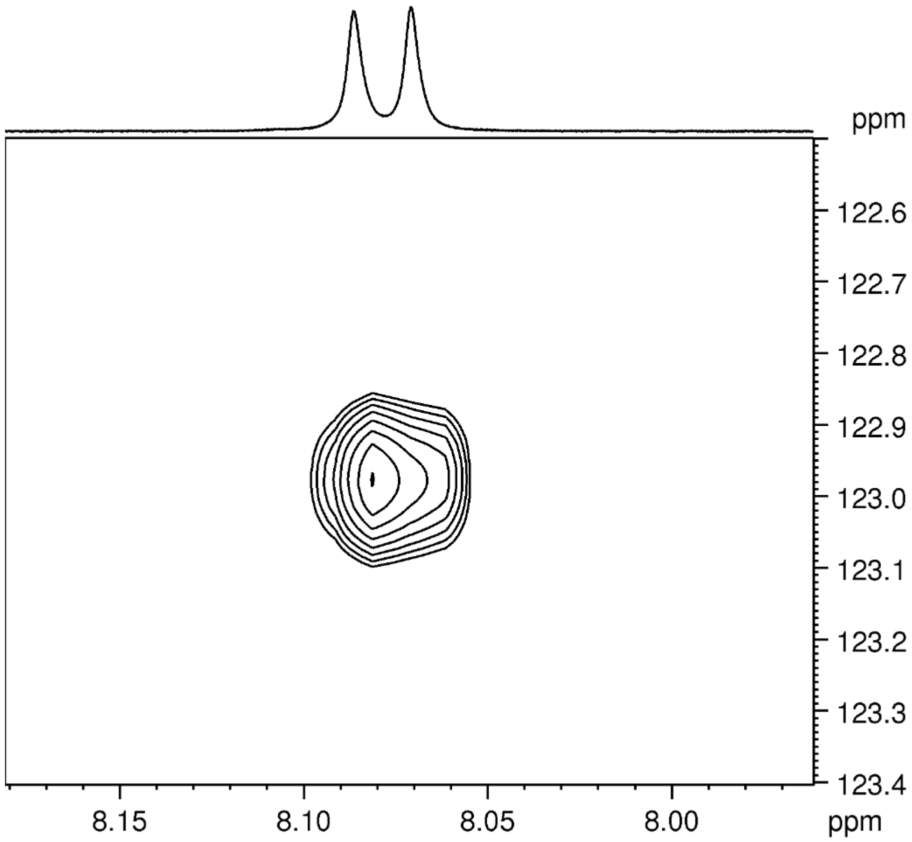

Figure S27. ^{1}H -^{15}N HSQC spectrum of 3'SL sodium salt (H_2O:D_2O 9:1 v/v solvent at pH 3.0)

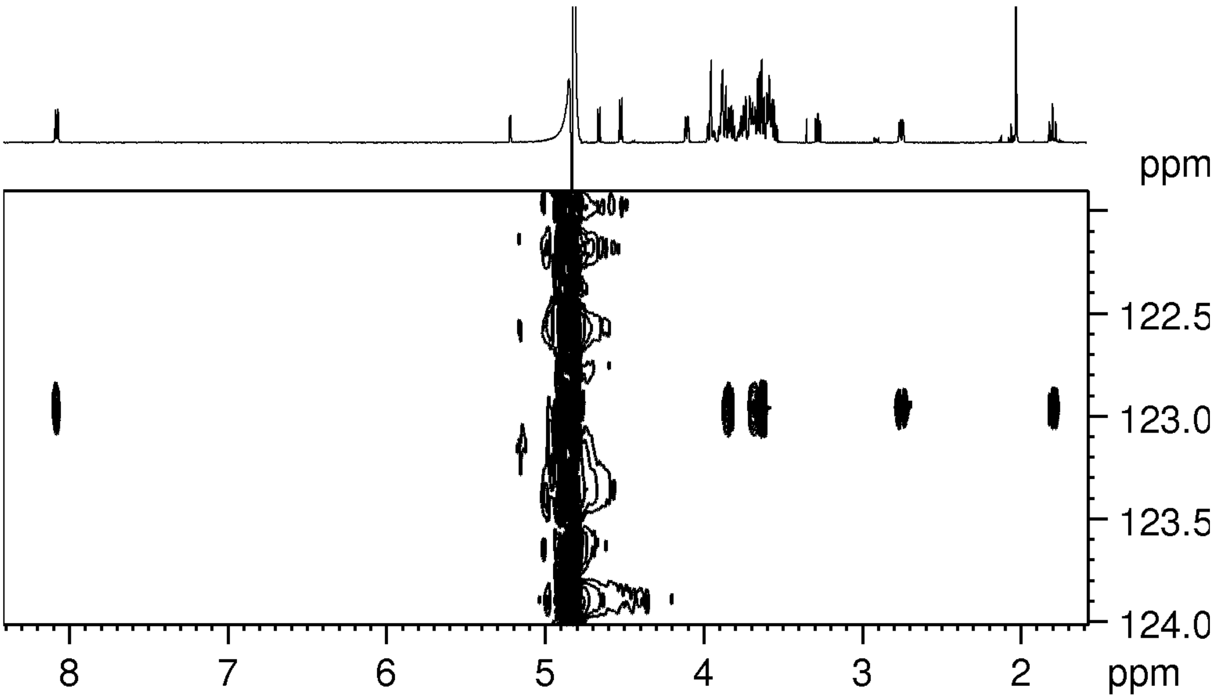

Figure S28. ^{1}H -^{15}N HSQC-TOCSY spectrum of 3’SL sodium salt (H_2O:D_2O 9:1 v/v solvent at pH 3.0)

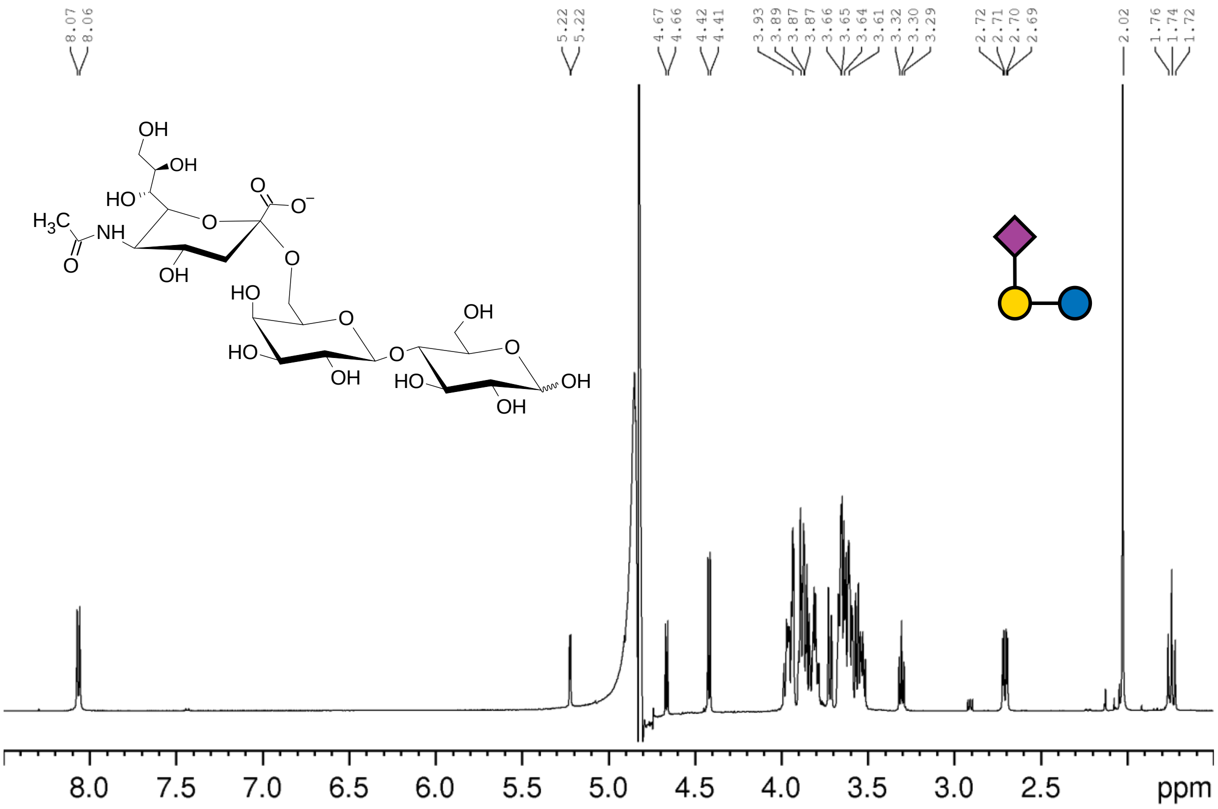

Figure S29. ^{1}H NMR spectrum of 6'SL sodium salt (H_2O:D_2O 9:1 v/v solvent at pH 3.0)

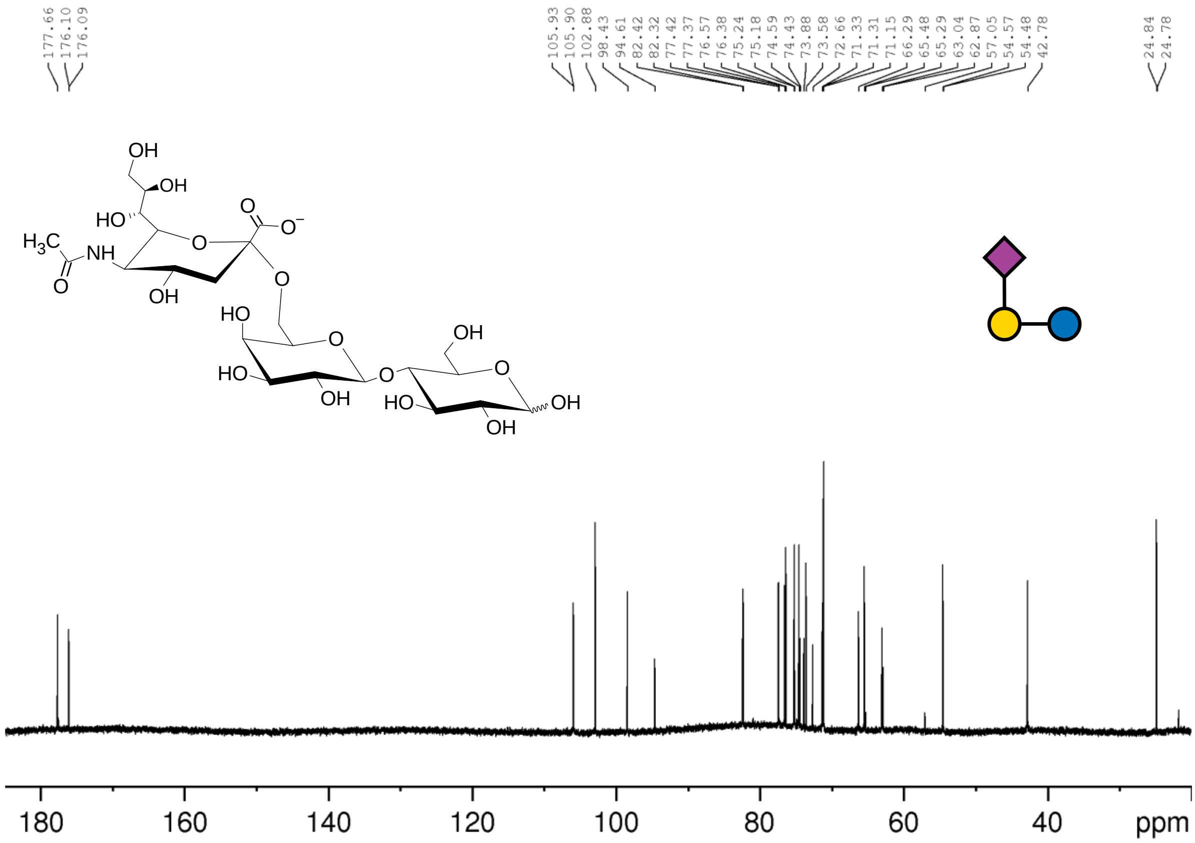

Figure S30. ^{13}C NMR spectrum of 6'SL sodium salt (H_2O:D_2O 9:1 v/v solvent at pH 3.0)

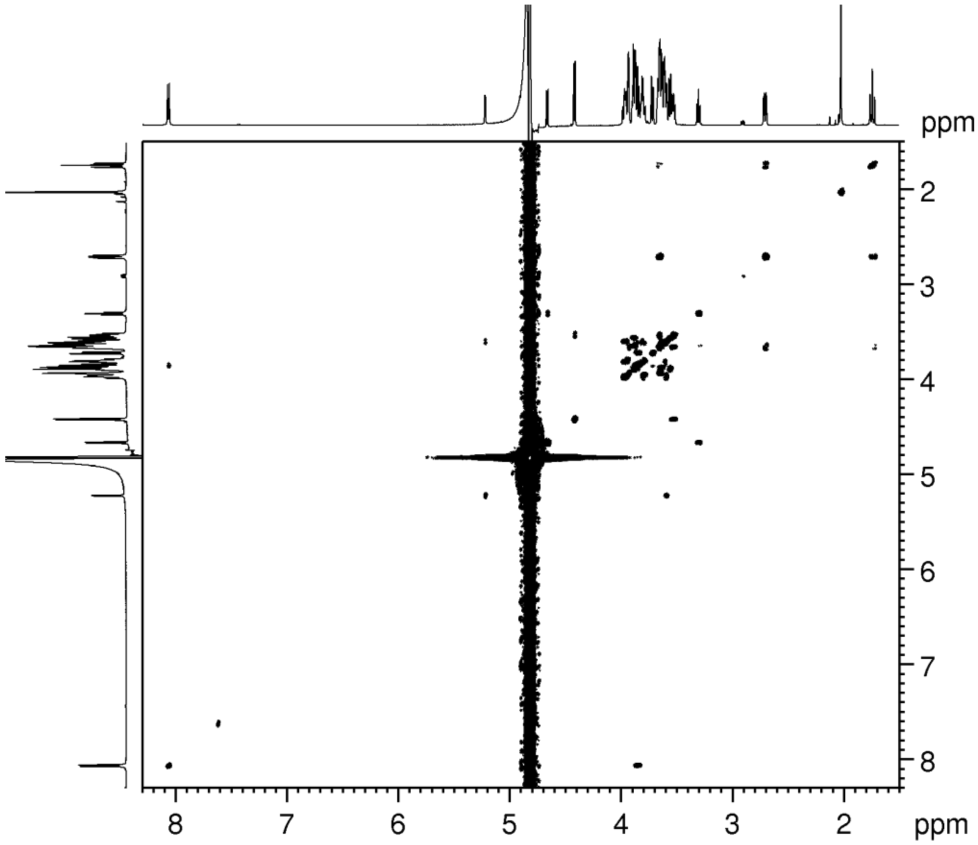

Figure S31. ^{1}H -^{1}H COSY spectrum of 6’SL sodium salt (H_2O:D_2O 9:1 v/v solvent at pH 3.0)

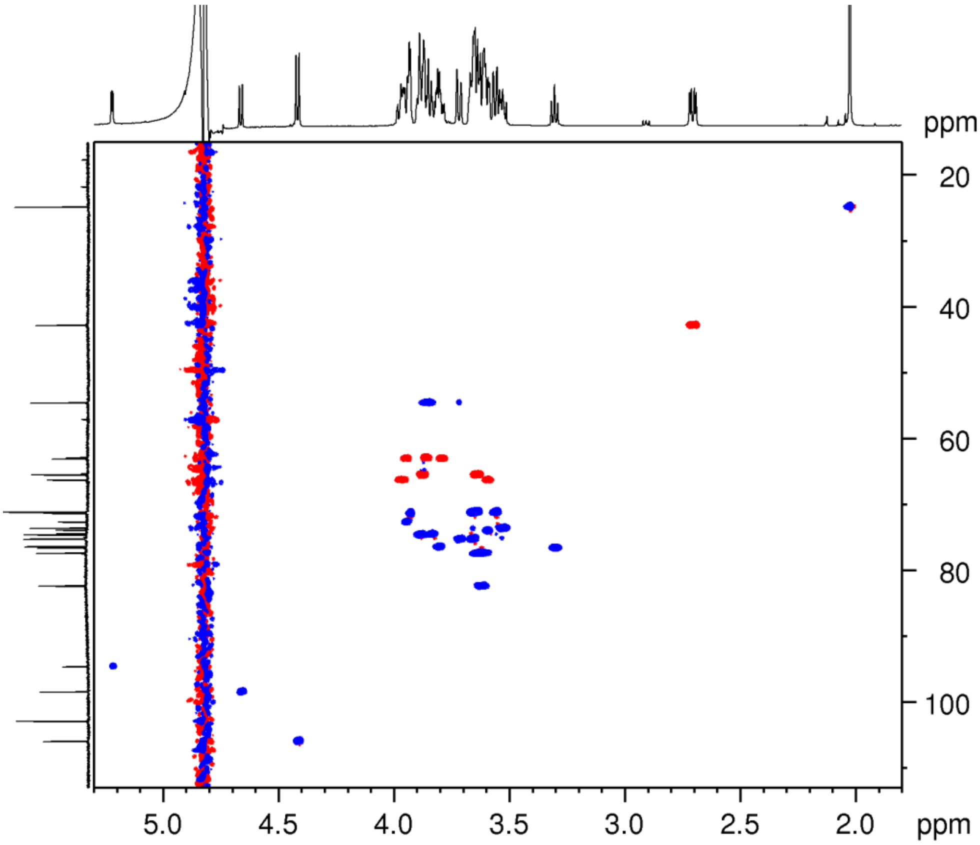

Figure S32. ^{1}H -^{13}C HSQC spectrum of 6’SL sodium salt (H_2O:D_2O 9:1 v/v solvent at pH 3.0)

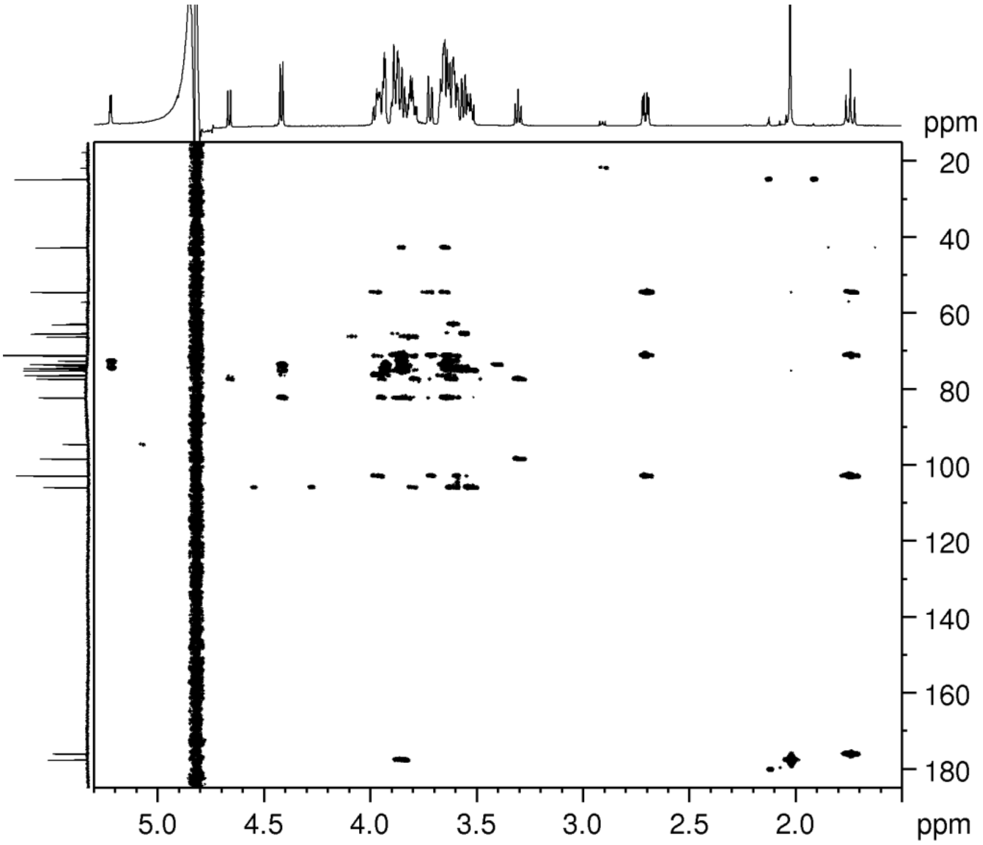

Figure S33. ^{1}H -^{13}C HMBC spectrum of 6’SL sodium salt (H_2O:D_2O 9:1 v/v solvent at pH 3.0)

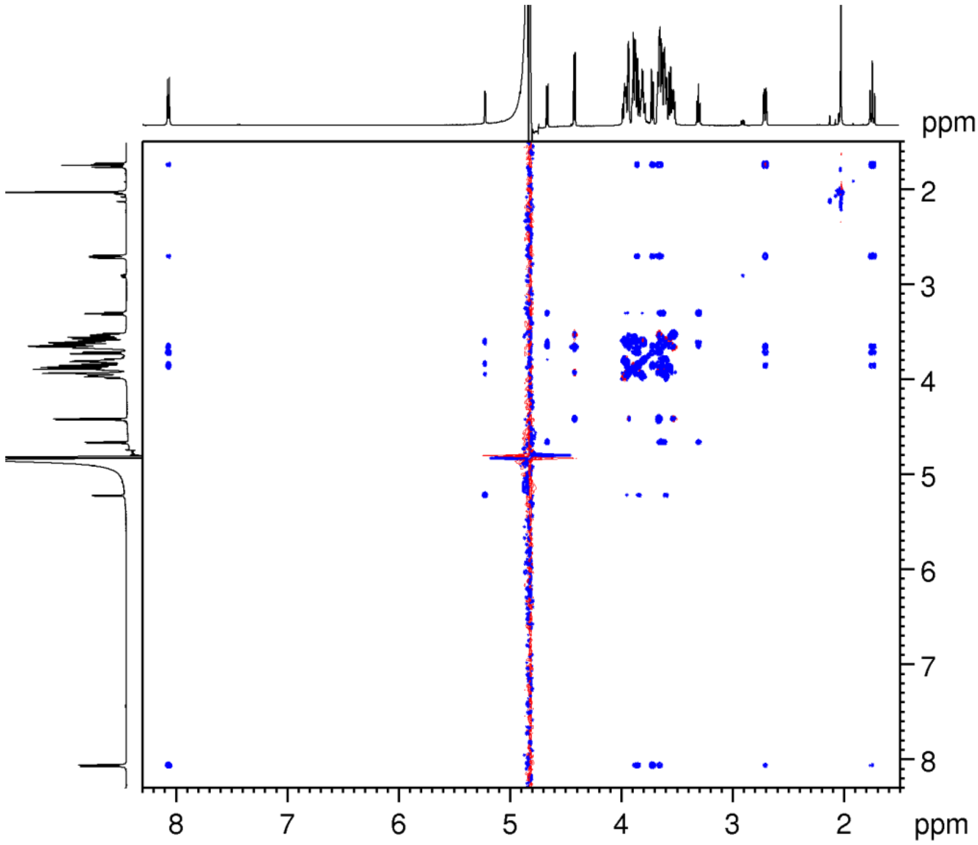

Figure S34. ^{1}H -^{1}H TOCSY spectrum of 6’SL sodium salt (H_2O:D_2O 9:1 v/v solvent at pH 3.0)

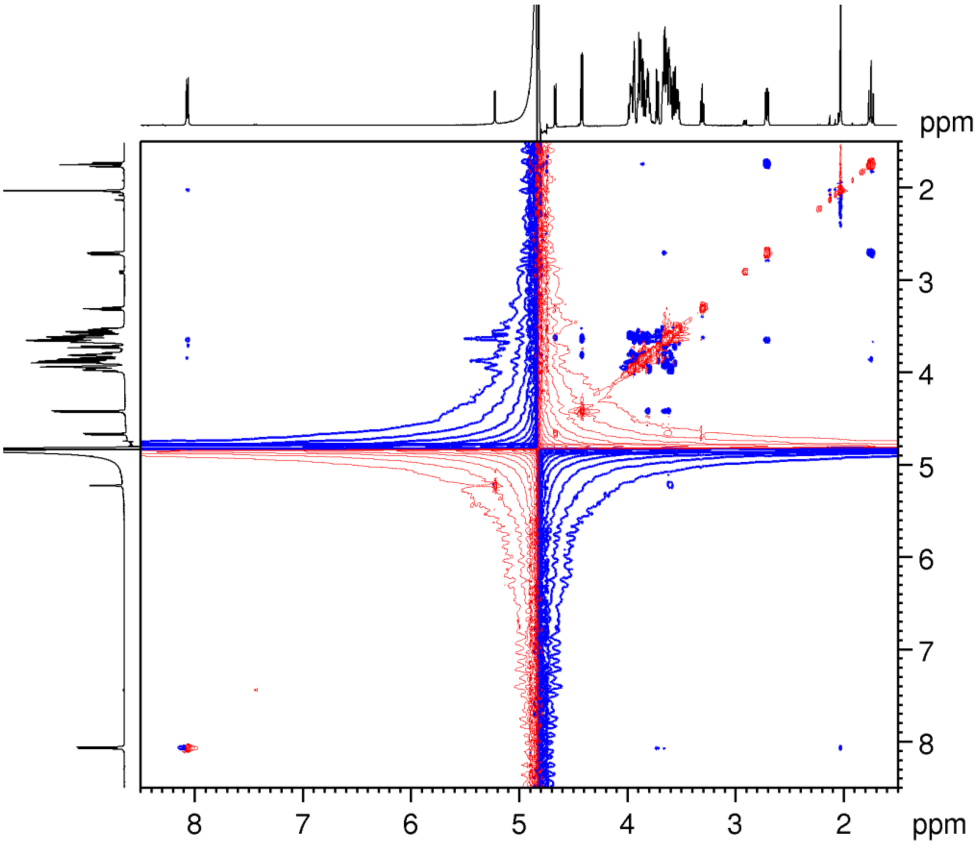

Figure S35. ^1H -^1H ROESY spectrum of 6'SL sodium salt (H_2O:D_2O 9:1 v/v solvent at pH 3.0)

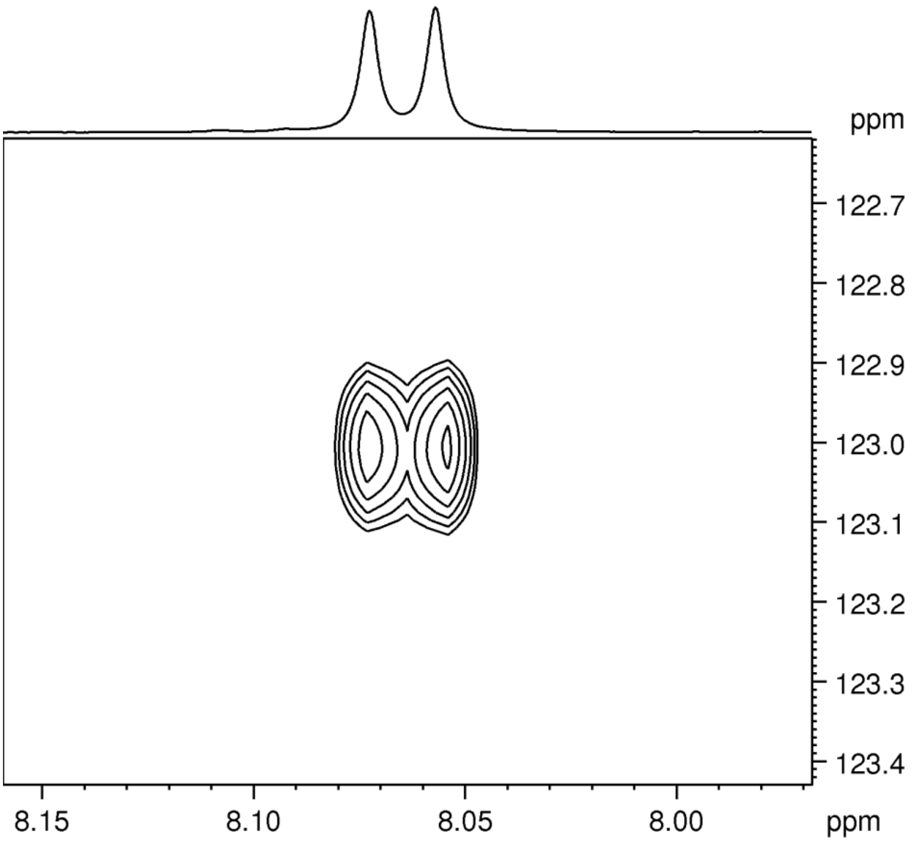

Figure S36. ^1H -^{15}N HSQC spectrum of 6'SL sodium salt (H_2O:D_2O 9:1 v/v solvent at pH 3.0)

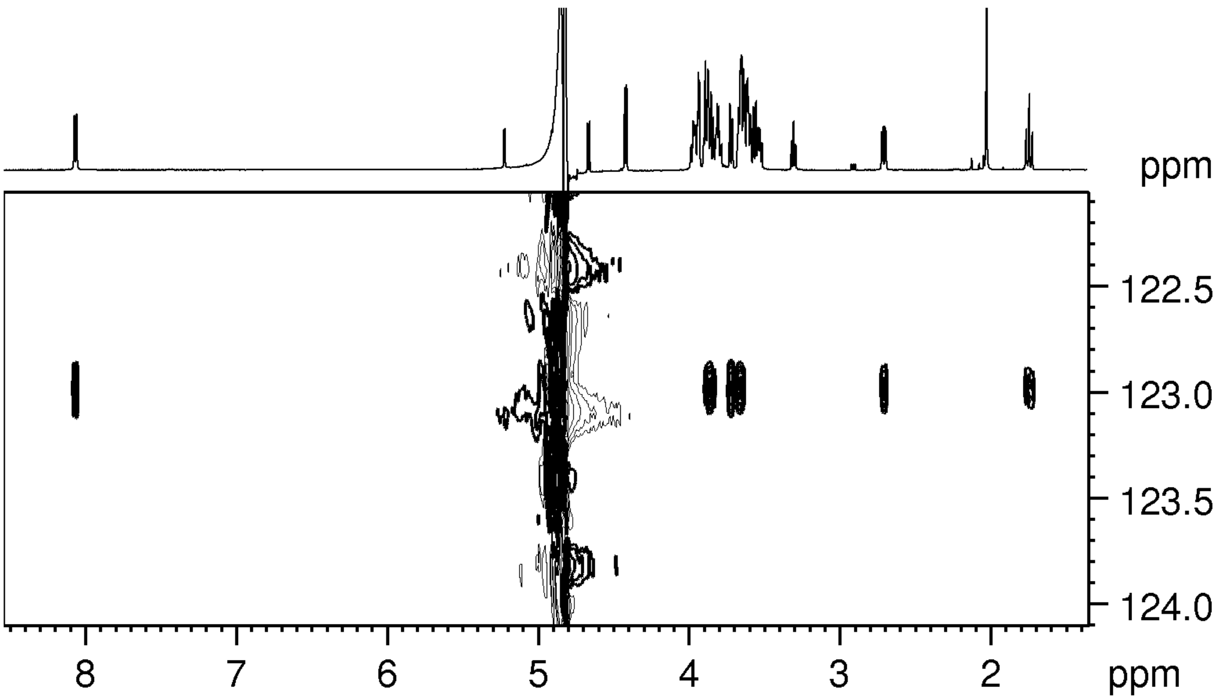

Figure S37. H -^{15}N HSQC-TOCSY spectrum of 6’SL sodium salt (H_2O:D_2O 9:1 v/v solvent at pH 3.0)

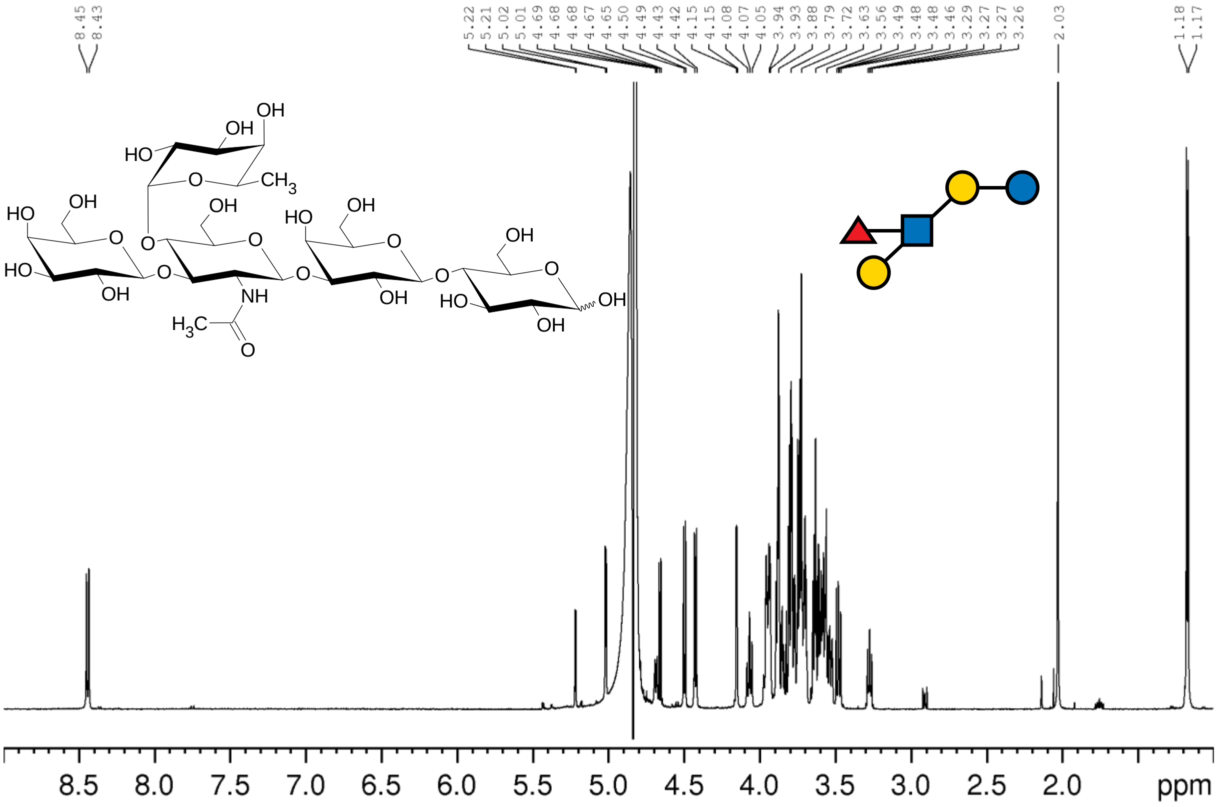

Figure S38. ^{1}H NMR spectrum of LNFP II (H_2O:D_2O 9:1 v/v solvent at pH 3.0)

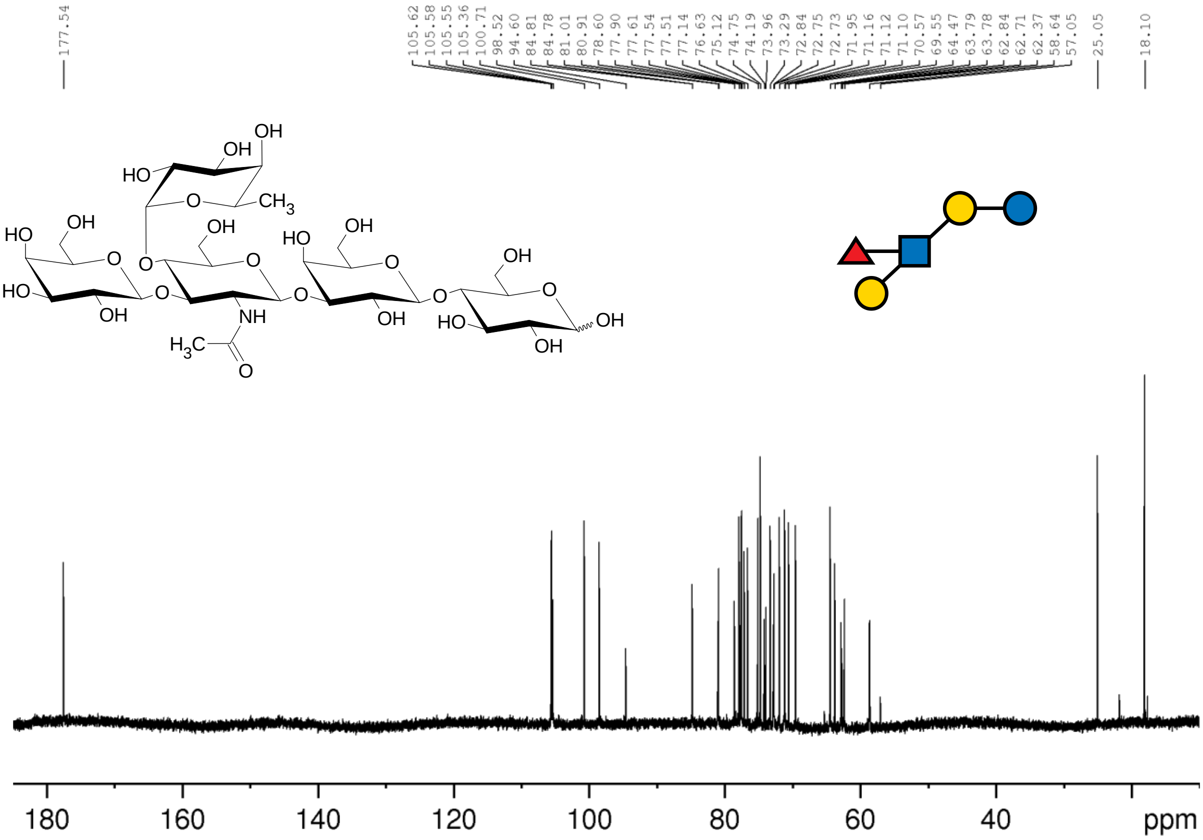

Figure S39. ^{13}C NMR spectrum of LNFP II (H_2O:D_2O 9:1 v/v solvent at pH 3.0)

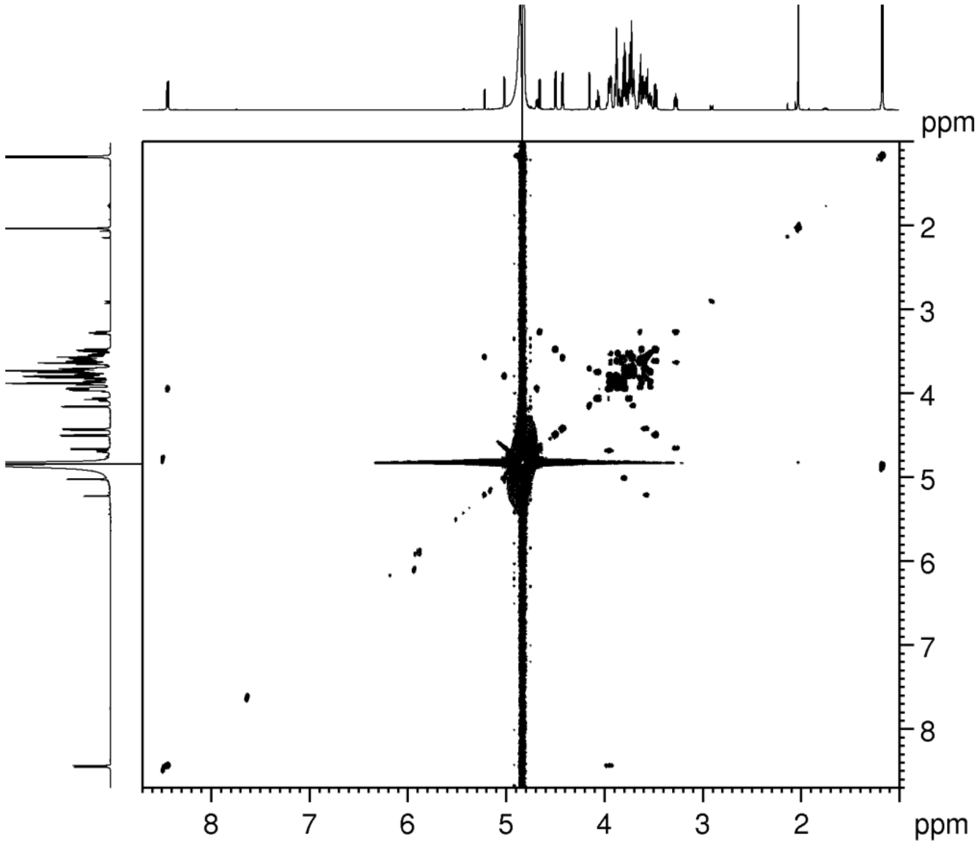

Figure S40. ^{1}H -^{1}H COSY spectrum of LNFP II (H_2O:D_2O 9:1 v/v solvent at pH 3.0)

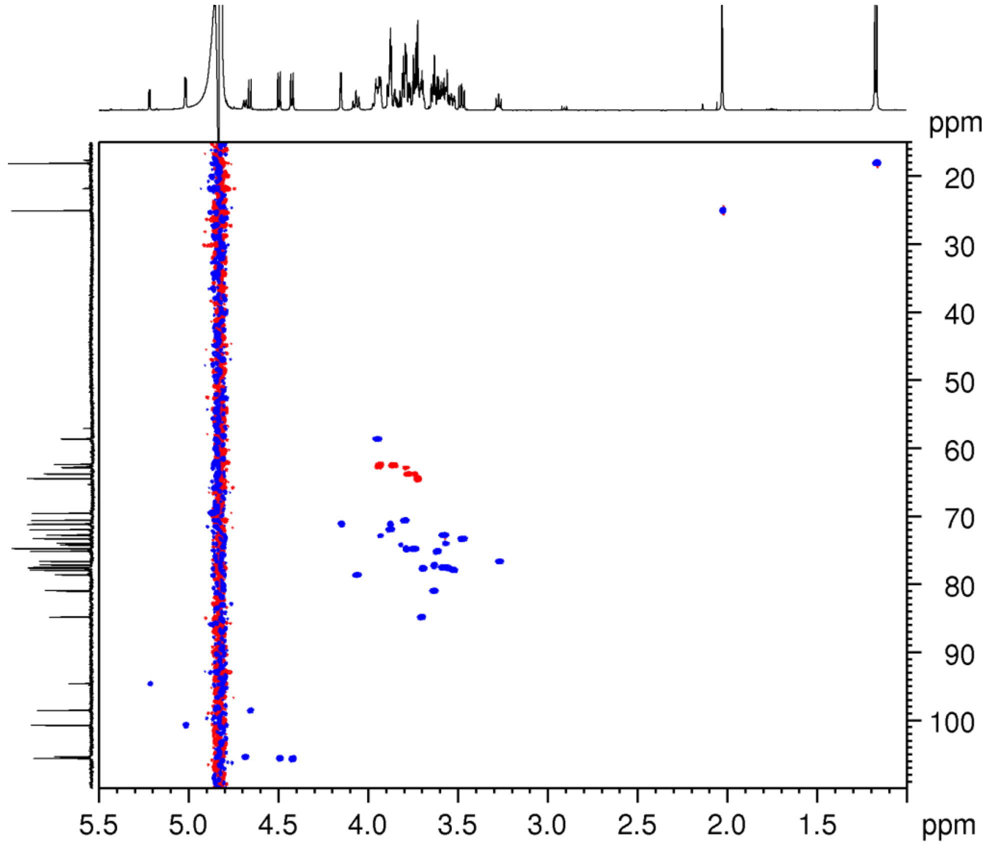

Figure S41. ^{1}H -^{13}C HSQC spectrum of LNFP II (H_2O:D_2O 9:1 v/v solvent at pH 3.0)

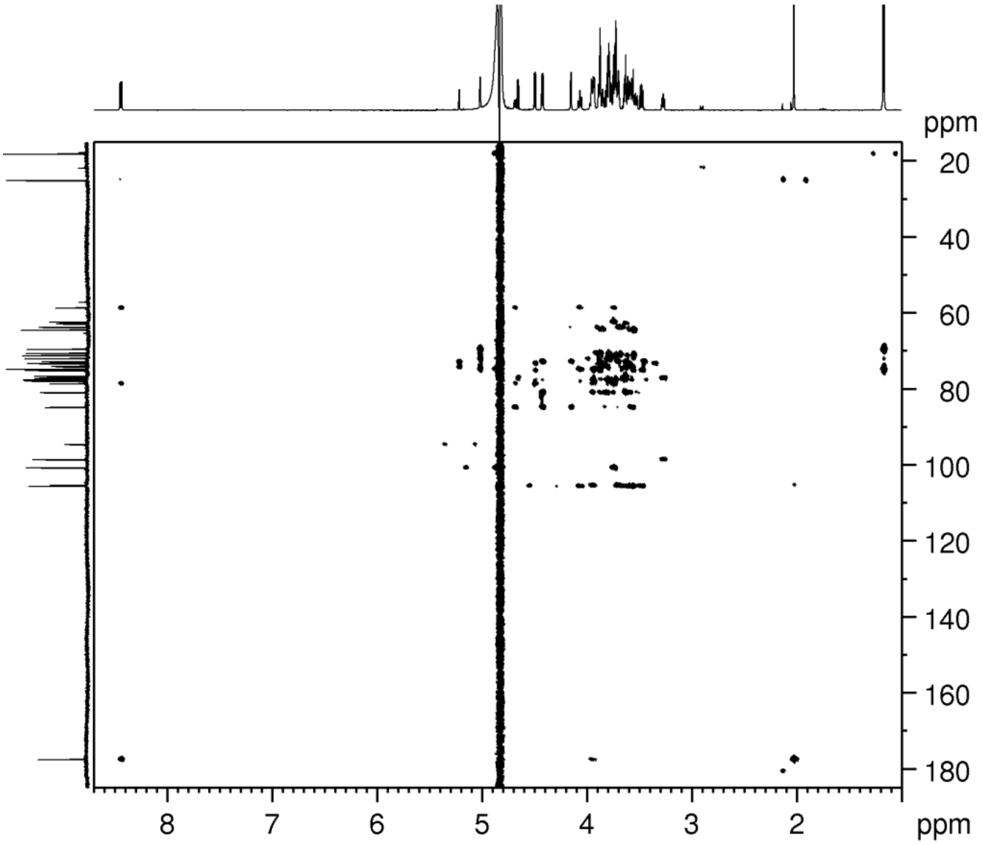

Figure S42. ^{1}H -^{13}C HMBC spectrum of LNFP II (H_2O:D_2O 9:1 v/v solvent at pH 3.0)

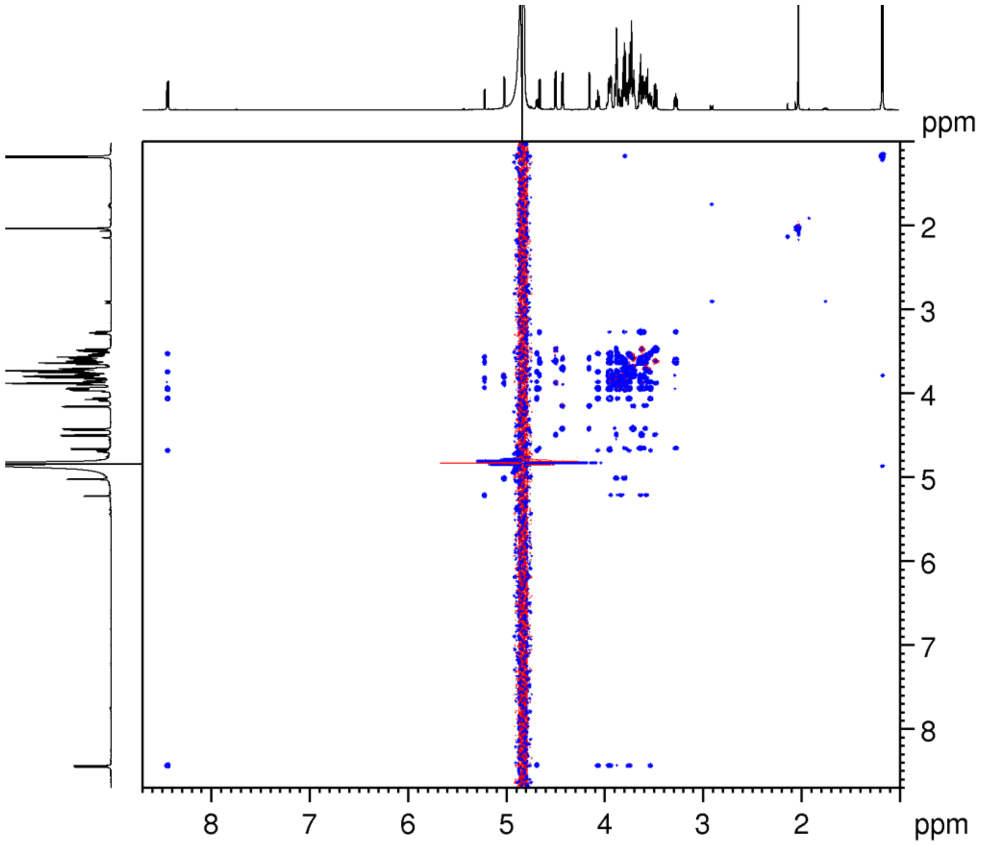

Figure S43. ^{1}H -^{1}H TOCSY spectrum of LNFP II (H_2O:D_2O 9:1 v/v solvent at pH 3.0)

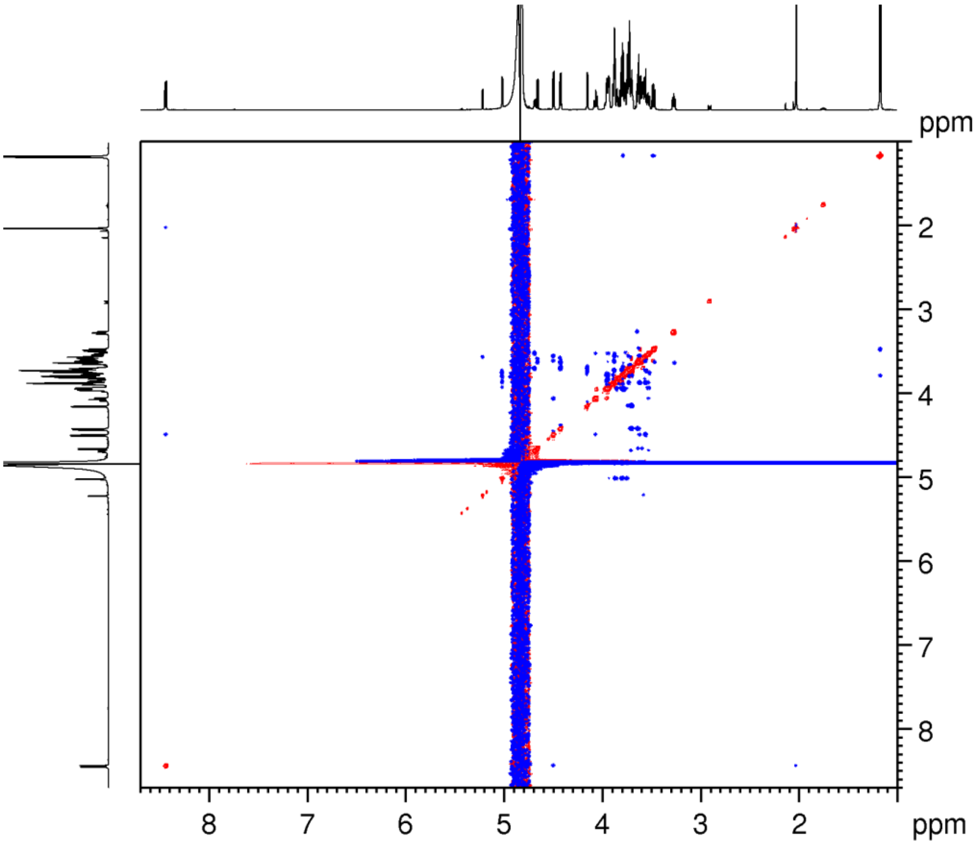

Figure S44. ^{1}H -^{1}H ROESY spectrum of LNFP II (H_2O:D_2O 9:1 v/v solvent at pH 3.0)

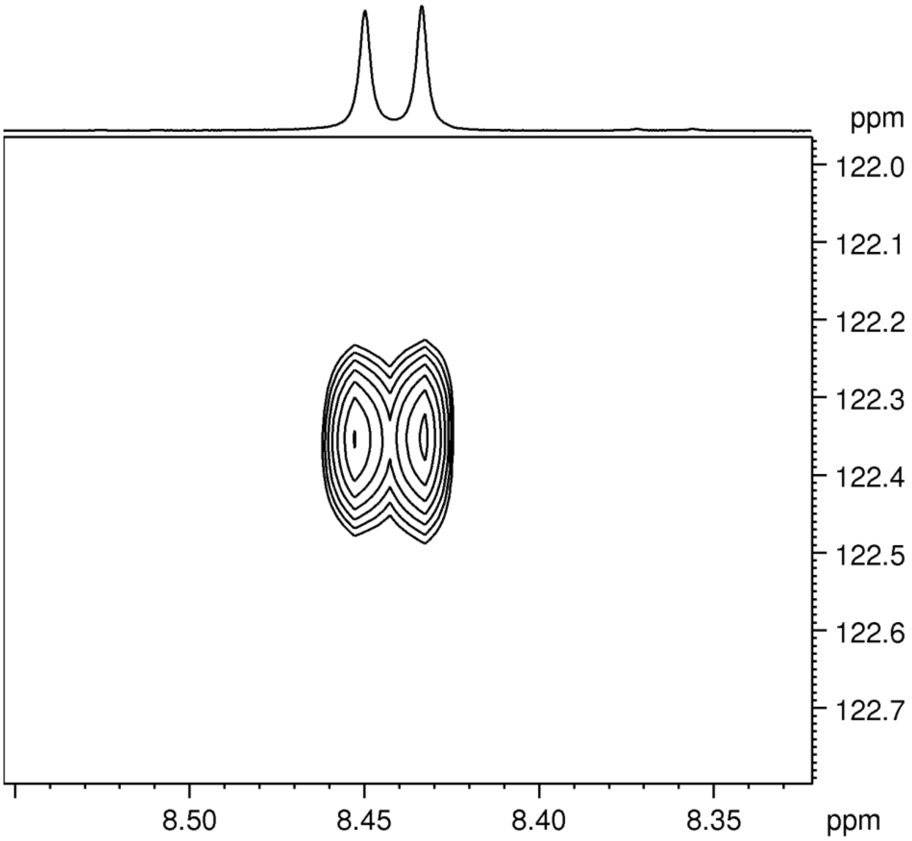

Figure S45. ^{1}H -^{15}N HSQC spectrum of LNFP II (H_2O:D_2O 9:1 v/v solvent at pH 3.0)

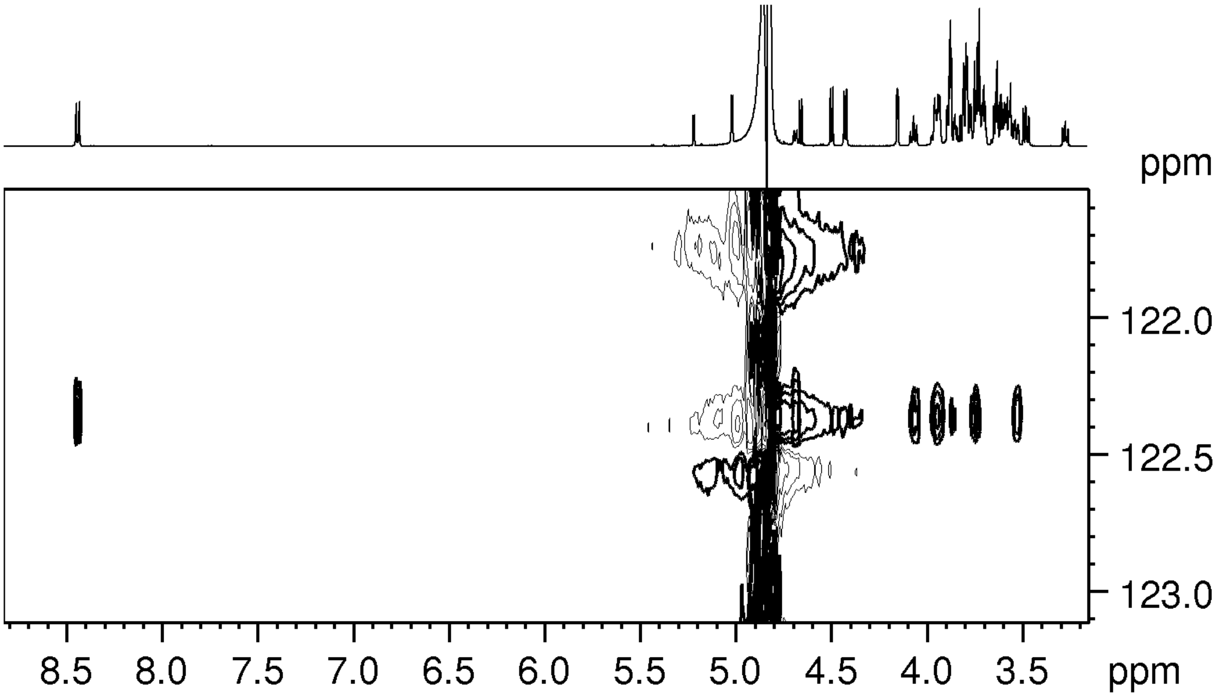

Figure S46. ^{1}H -^{15}N HSQC-TOCSY spectrum of LNFP II (H_2O:D_2O 9:1 v/v solvent at pH 3.0)

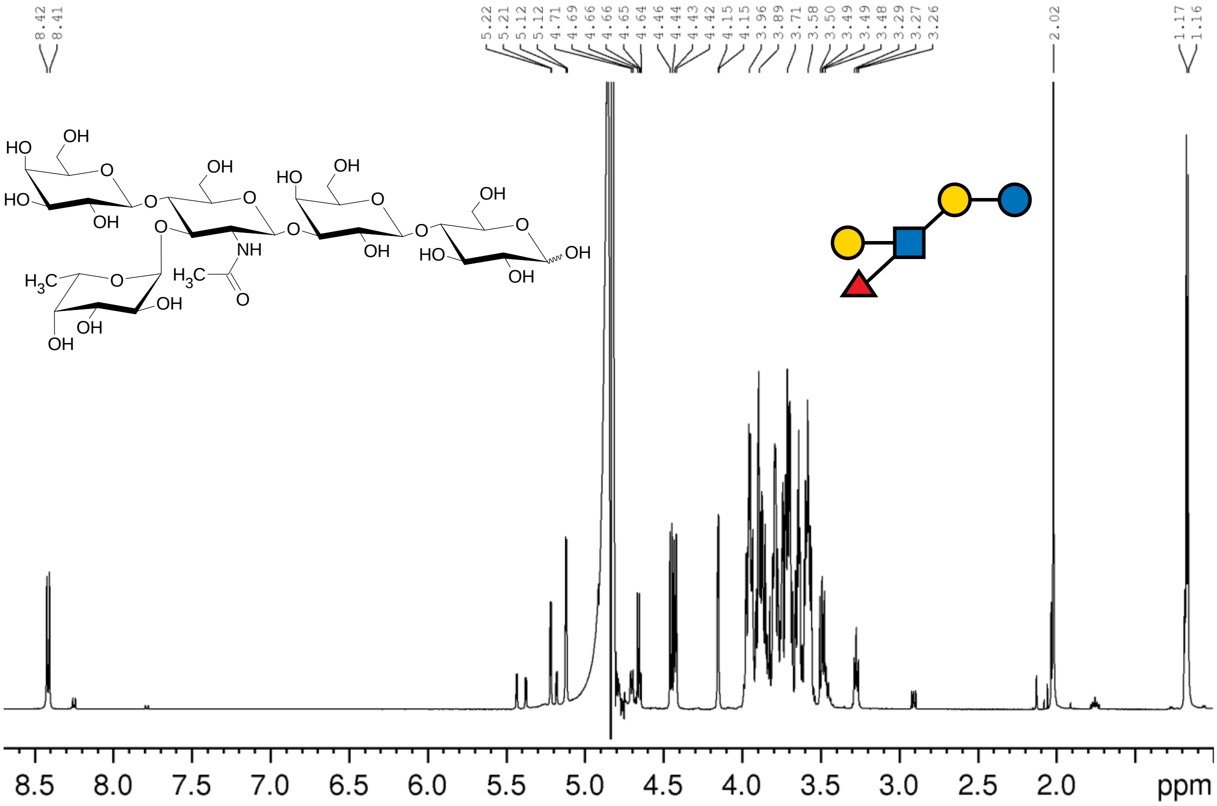

Figure S47. ^{1}H NMR spectrum of LNFP III (H_2O:D_2O 9:1 v/v solvent at pH 3.0)

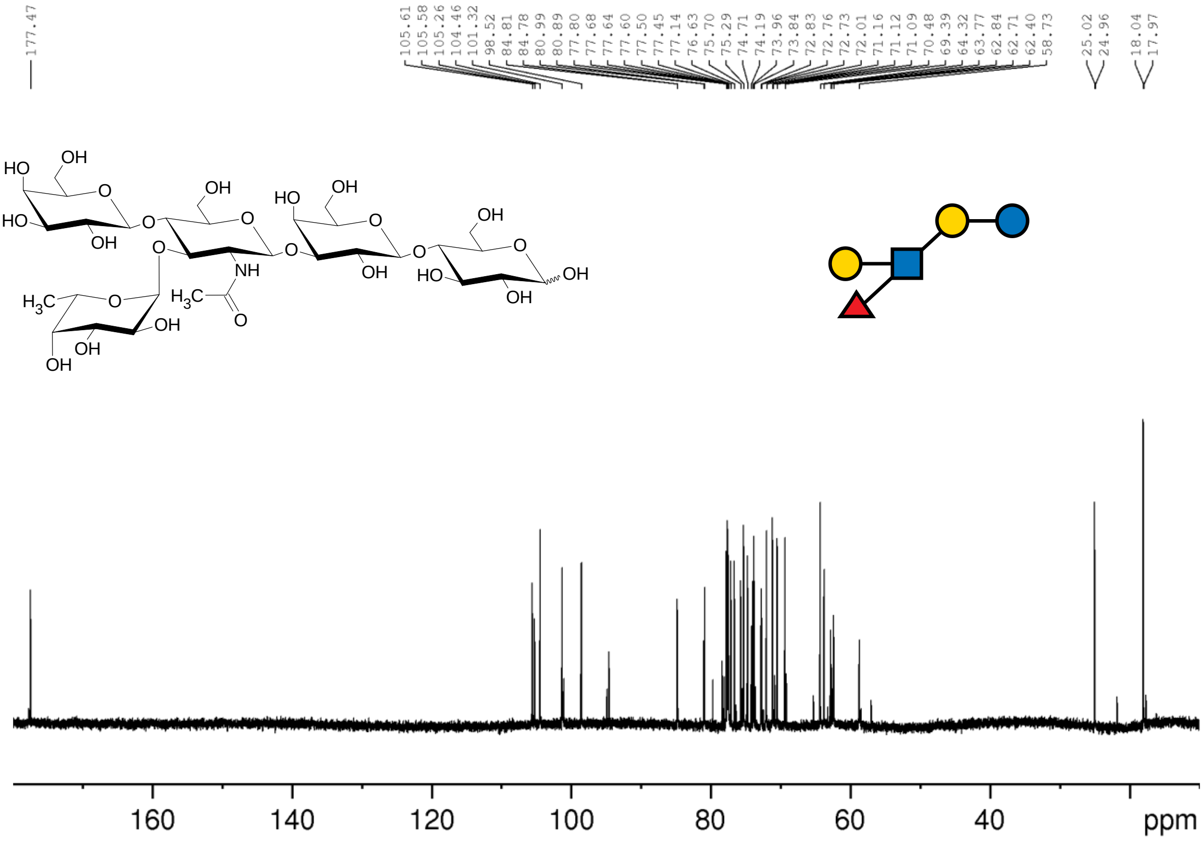

Figure S48. ^{13}C NMR spectrum of LNFP III (H_2O:D_2O 9:1 v/v solvent at pH 3.0)

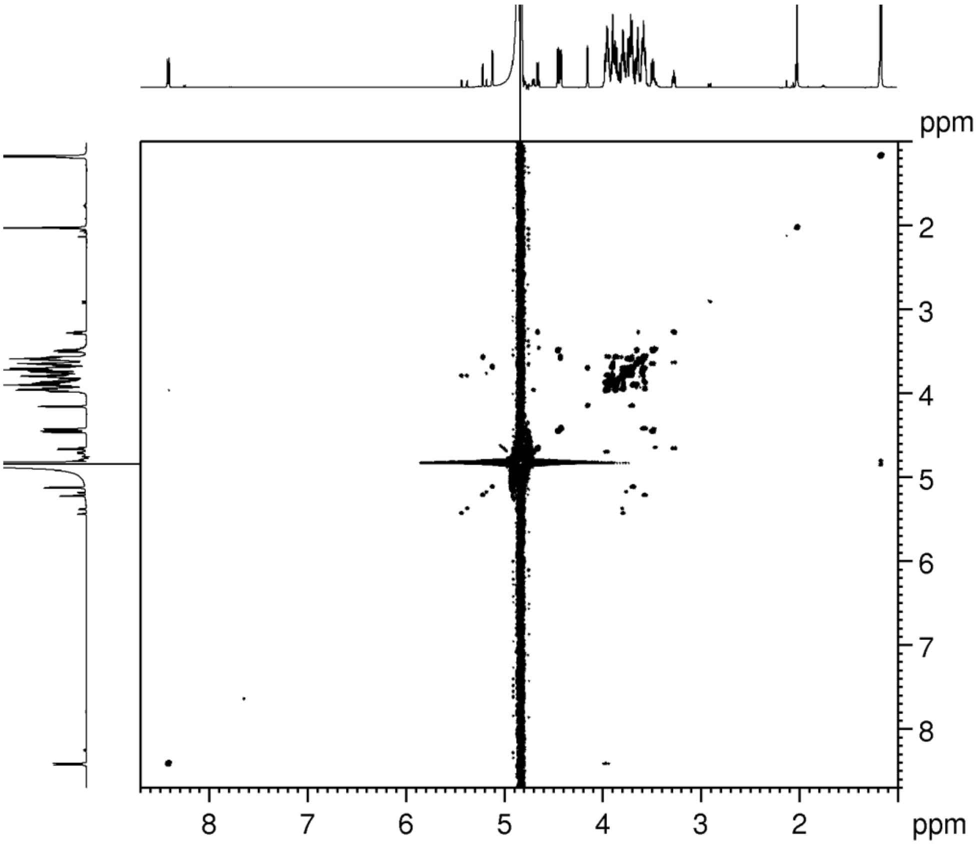

Figure S49. ^{1}H -^{1}H COSY spectrum of LNFP III (H_2O:D_2O 9:1 v/v solvent at pH 3.0)

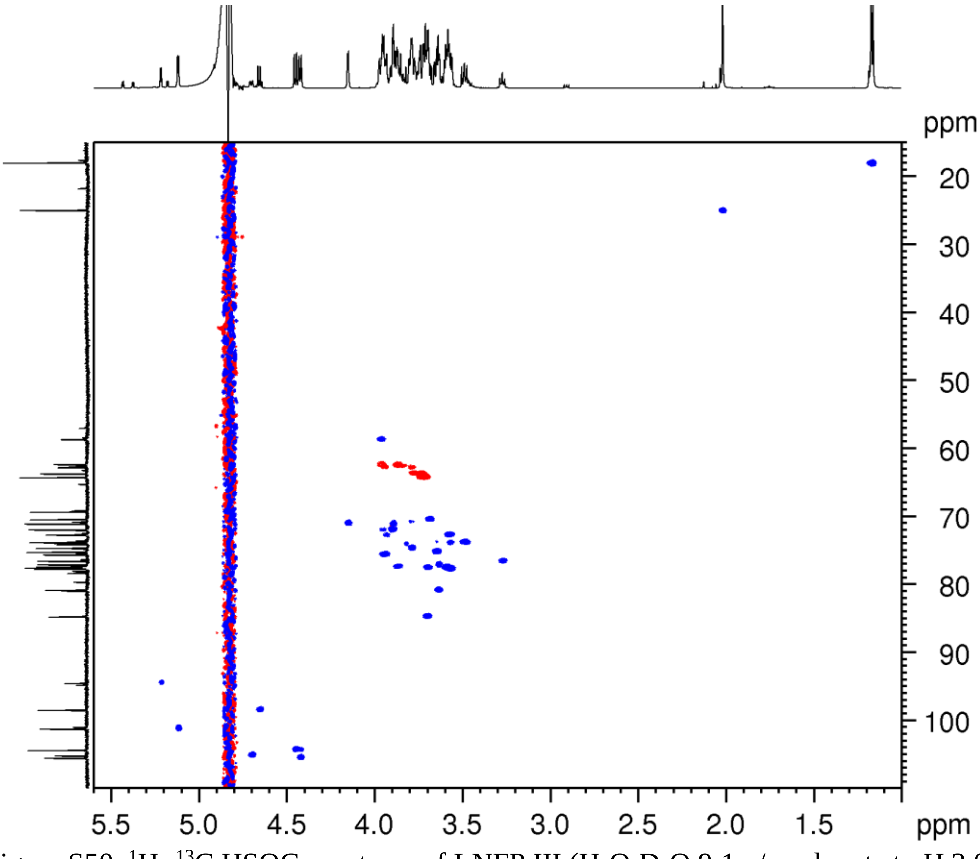

Figure S50. ^{1}H -^{13}C HSQC spectrum of LNFP III (H_2O:D_2O 9:1 v/v solvent at pH 3.0)

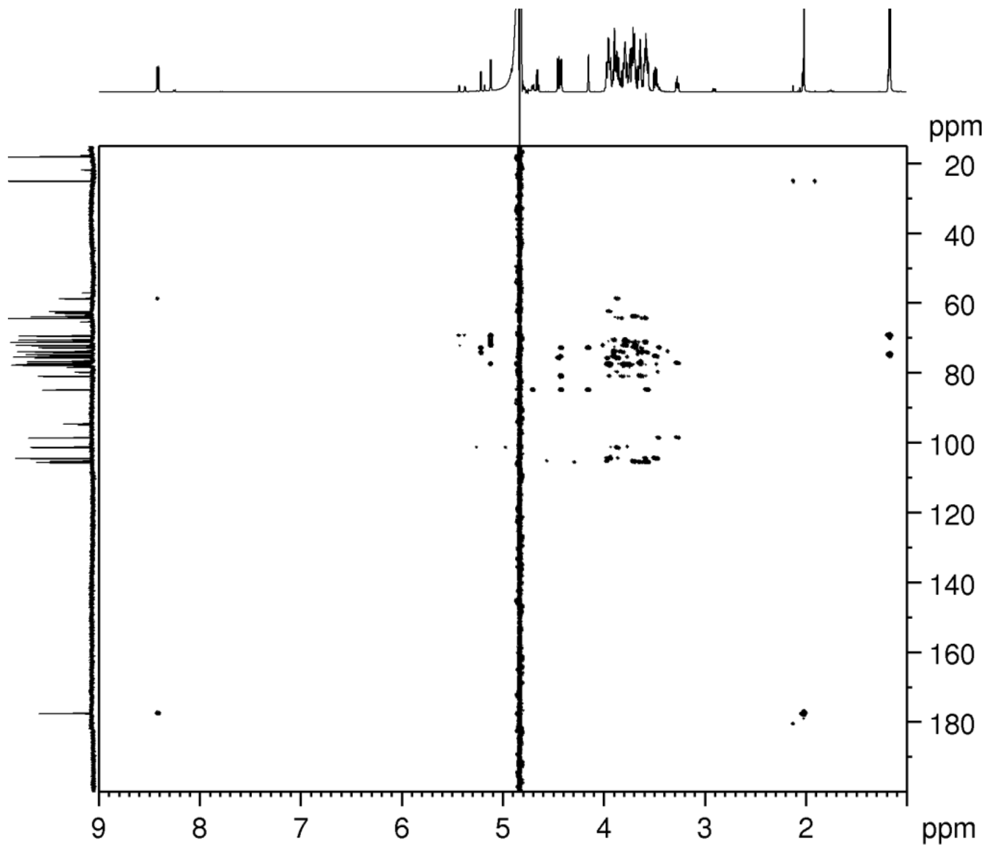

Figure S51. ^{1}H -^{13}C HMBC spectrum of LNFP III (H_2O:D_2O 9:1 v/v solvent at pH 3.0)

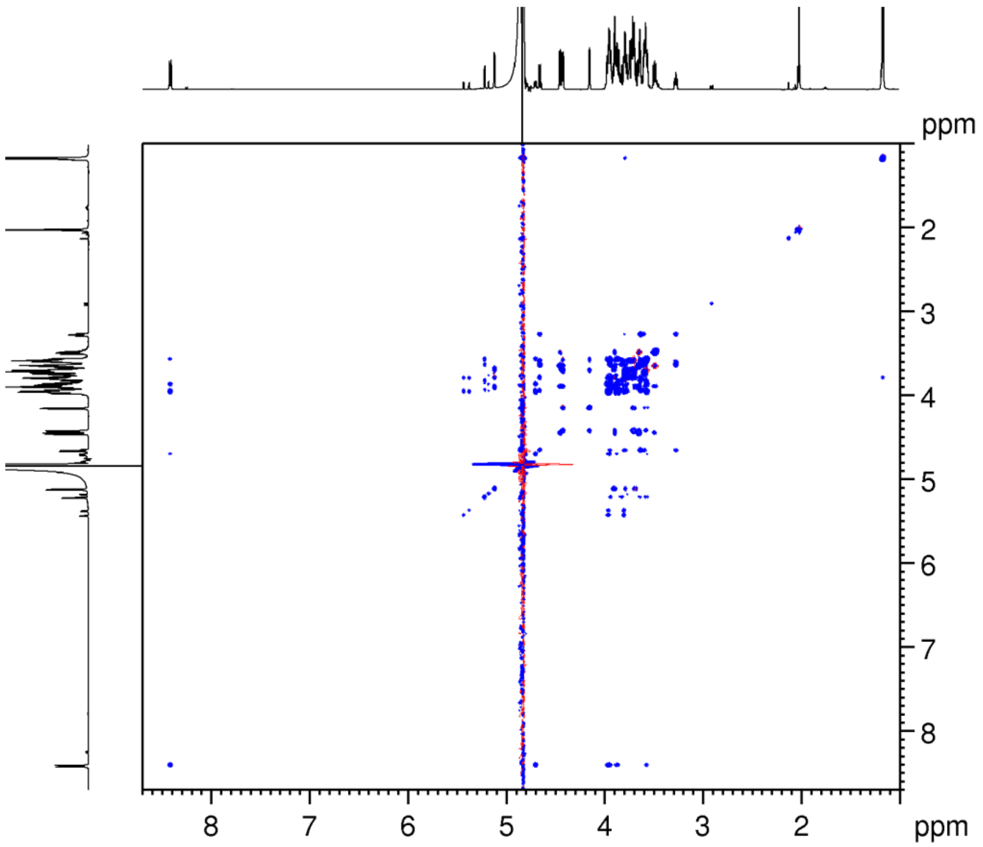

Figure S52. ^{1}H -^{1}H TOCSY spectrum of LNFP III (H_2O:D_2O 9:1 v/v solvent at pH 3.0)

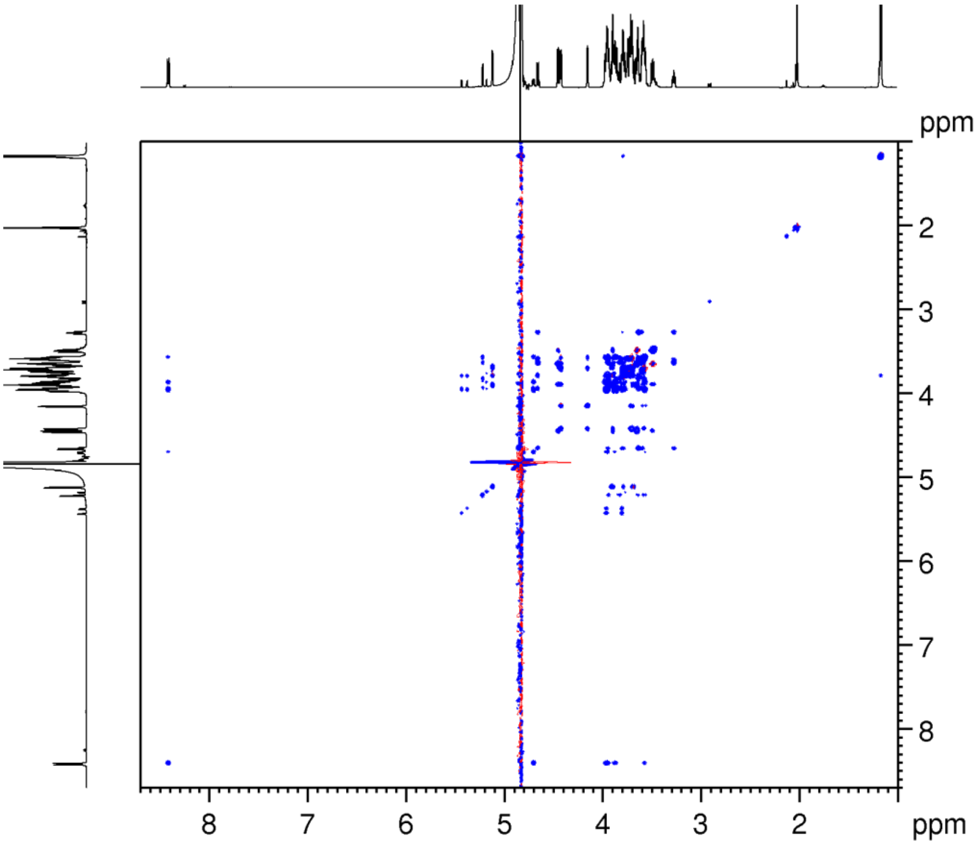

Figure S53. ^{1}H -^{1}H ROESY spectrum of LNFP III (H_2O:D_2O 9:1 v/v solvent at pH 3.0)

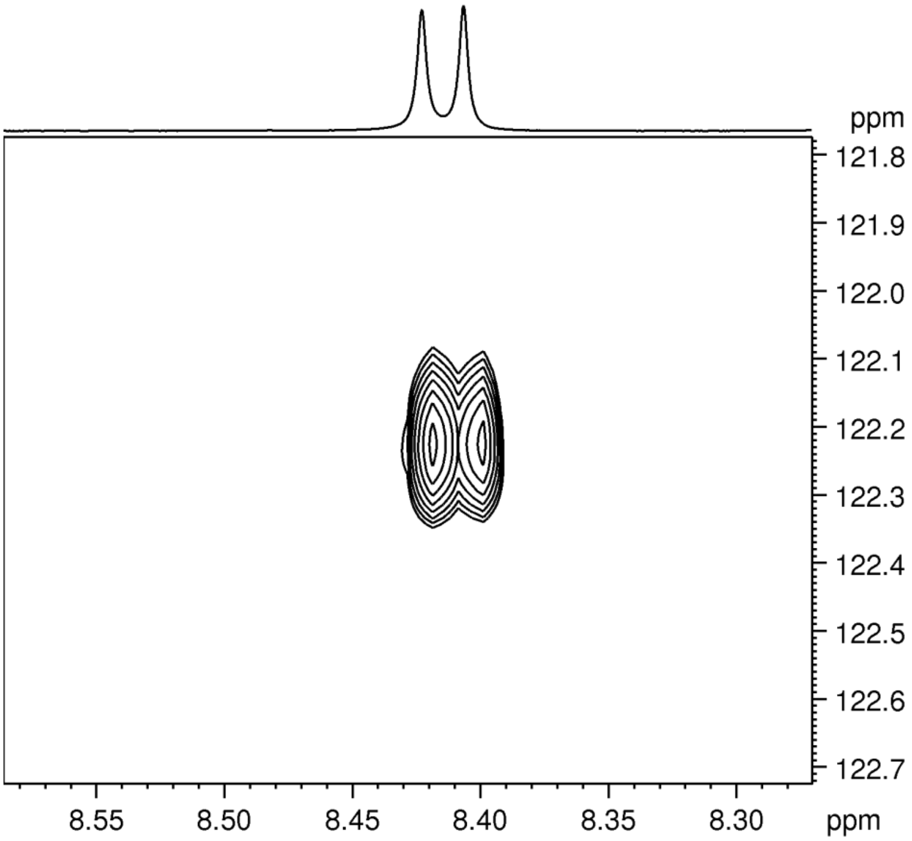

Figure S54. ^{1}H -^{15}N HSQC spectrum of LNFP III (H_2O:D_2O 9:1 v/v solvent at pH 3.0)

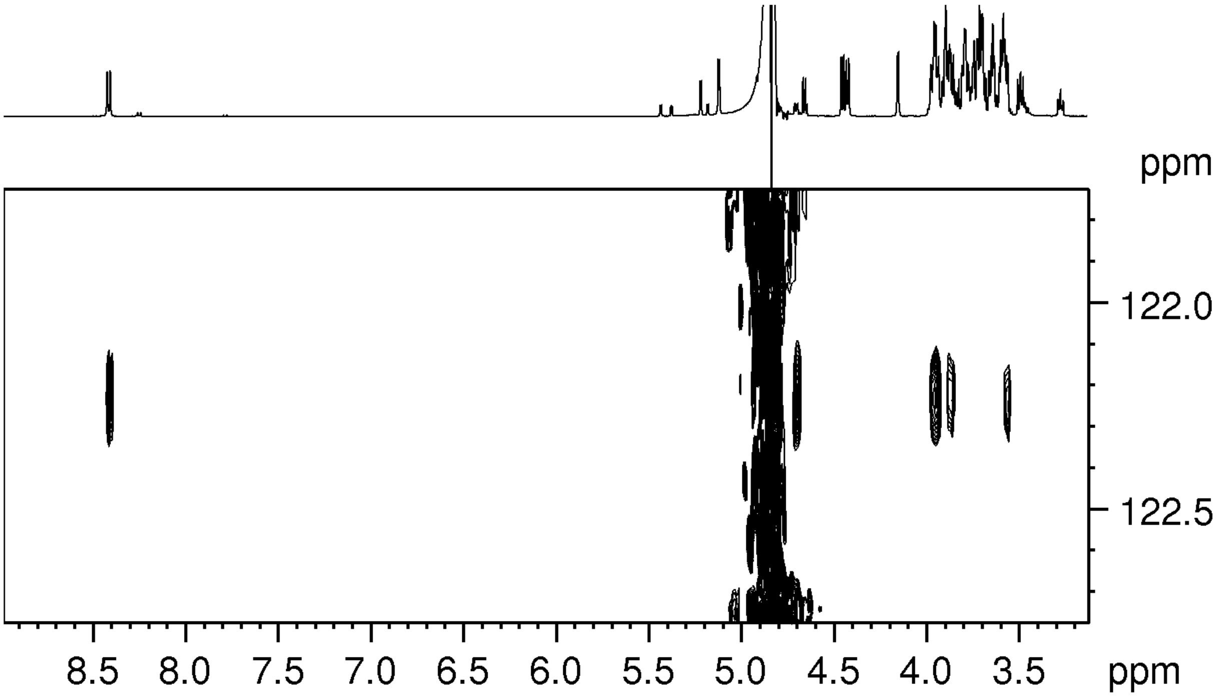

Figure S55. ^{1}H -^{15}N HSQC-TOCSY spectrum of LNFP III (H_2O:D_2O 9:1 v/v solvent at pH 3.0)

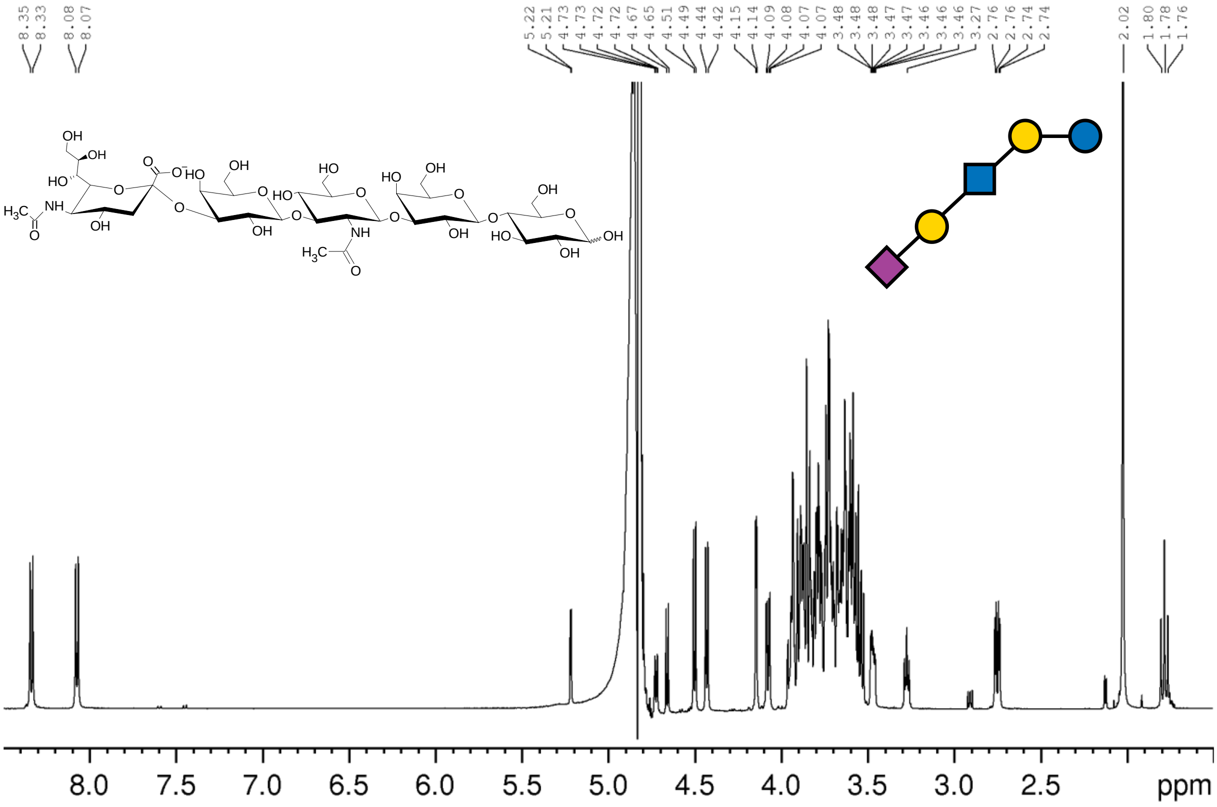

Figure S56. ^{1}H NMR spectrum of LSTa (H_2O:D_2O 9:1 v/v solvent at pH 3.0)

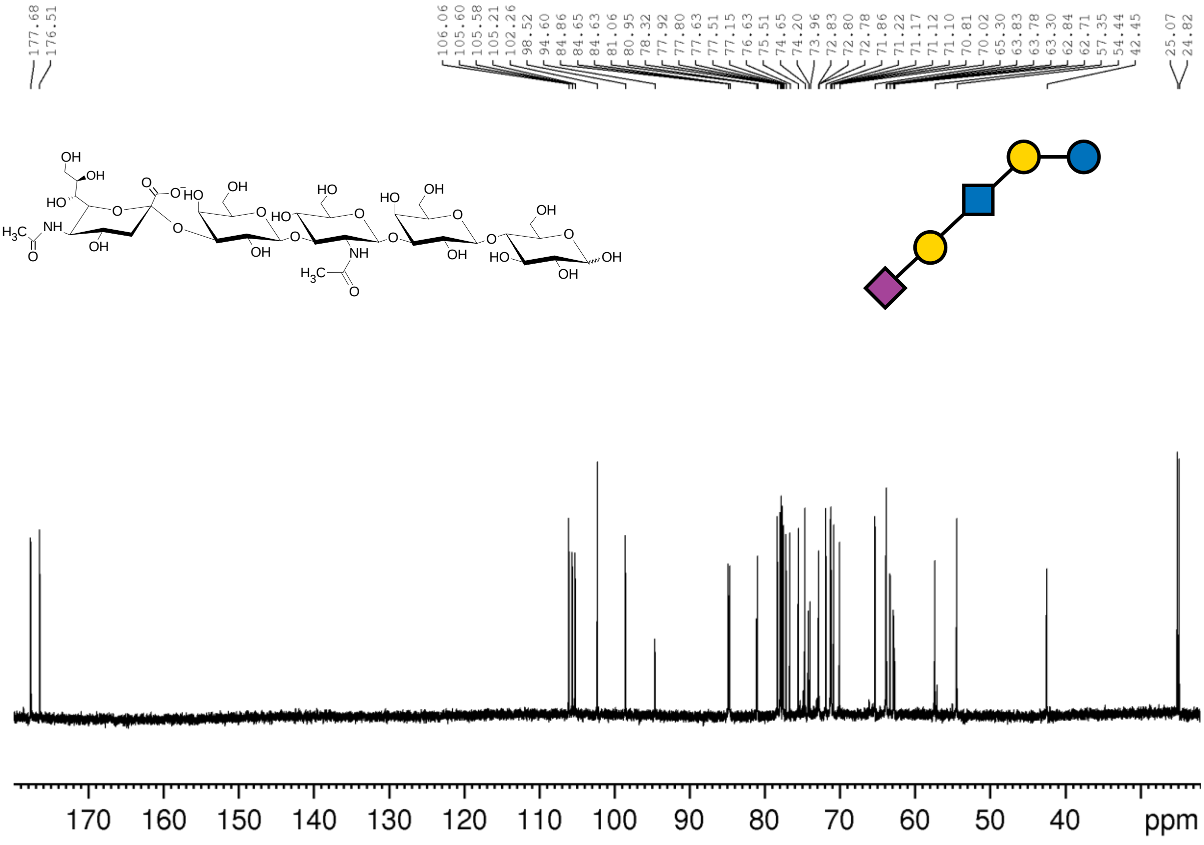

Figure S57. ^{13}C NMR spectrum of LSTa (H_2O:D_2O 9:1 v/v solvent at pH 3.0)

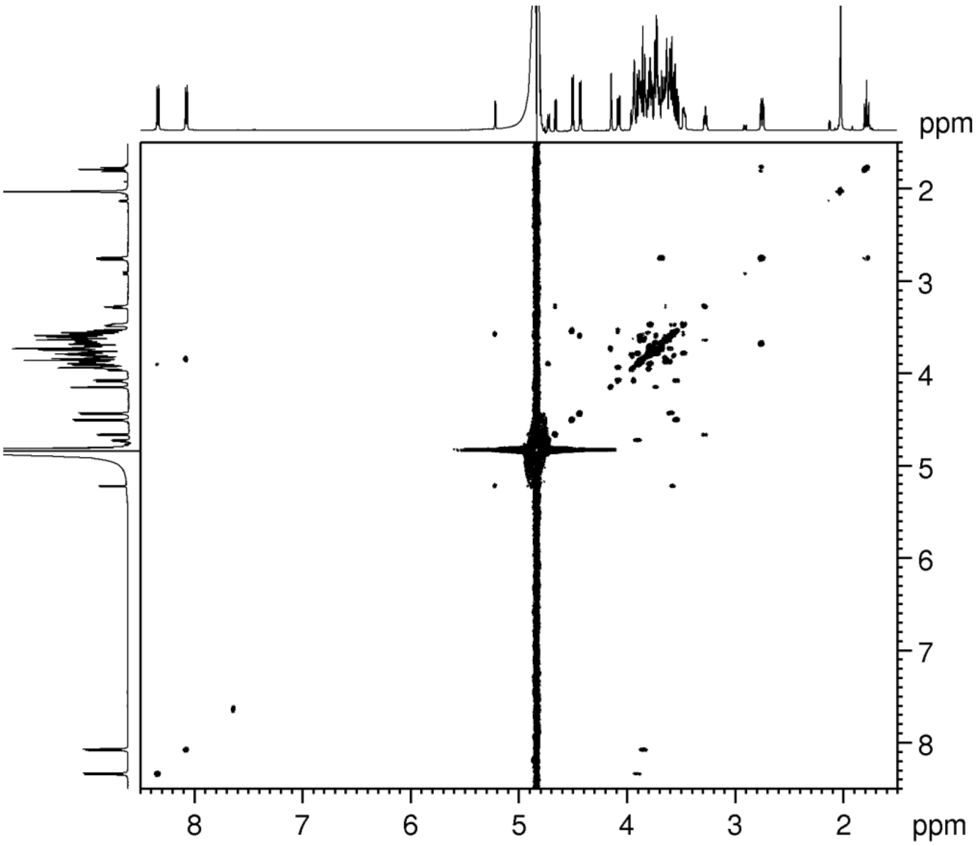

Figure S58. ^{1}H -^{1}H COSY spectrum of LSTa (H_2O:D_2O 9:1 v/v solvent at pH 3.0)

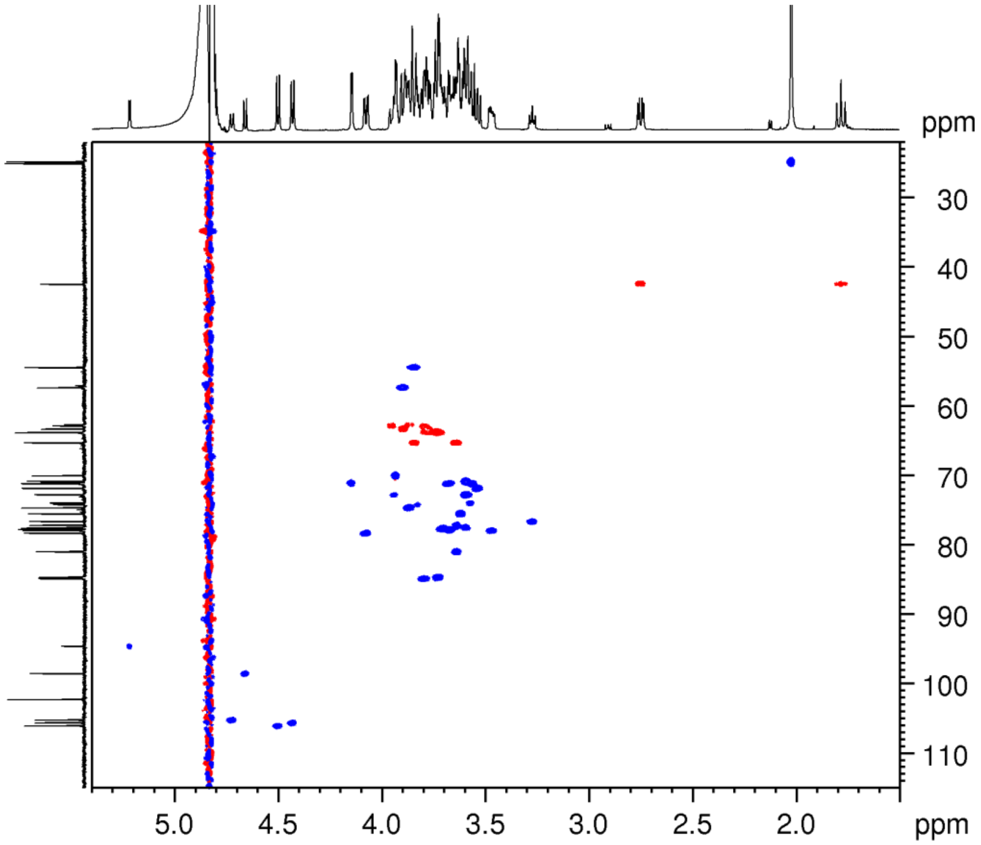

Figure S59. ^{1}H -^{13}C HSQC spectrum of LSTa (H_2O:D_2O 9:1 v/v solvent at pH 3.0)

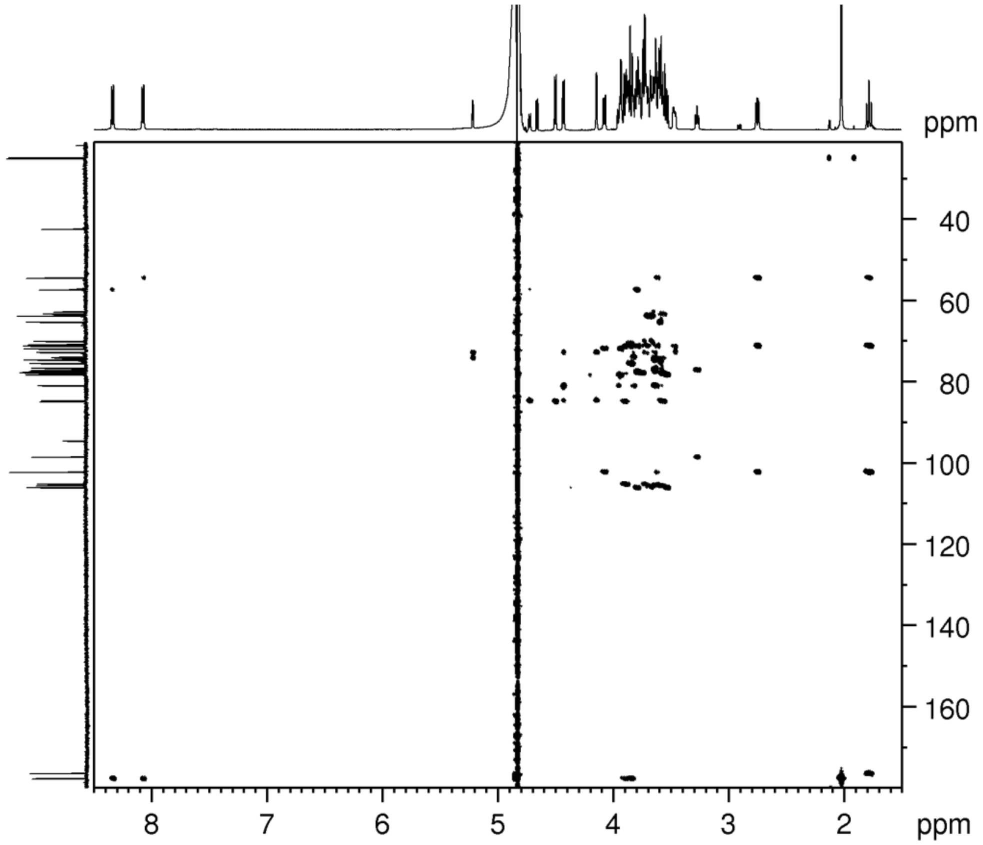

Figure S60. ^{1}H -^{13}C HMBC spectrum of LSTa (H_2O:D_2O 9:1 v/v solvent at pH 3.0)

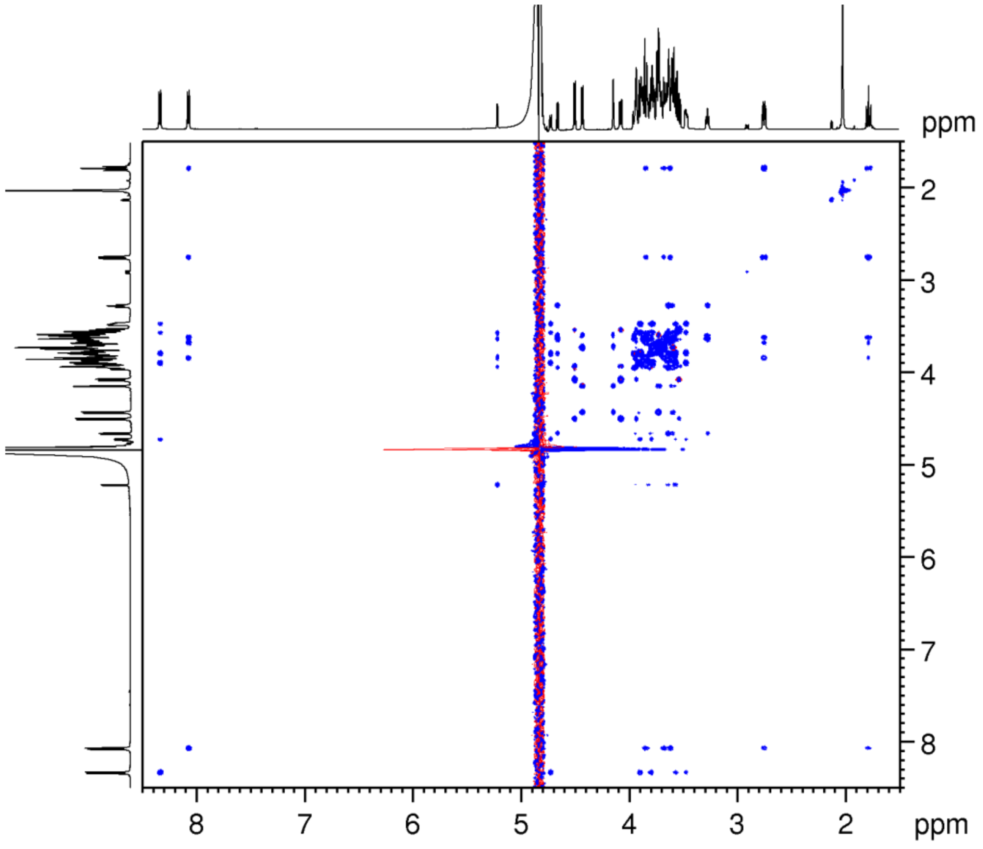

Figure S61. ^{1}H -^{1}H TOCSY spectrum of LSTa (H_2O:D_2O 9:1 v/v solvent at pH 3.0)

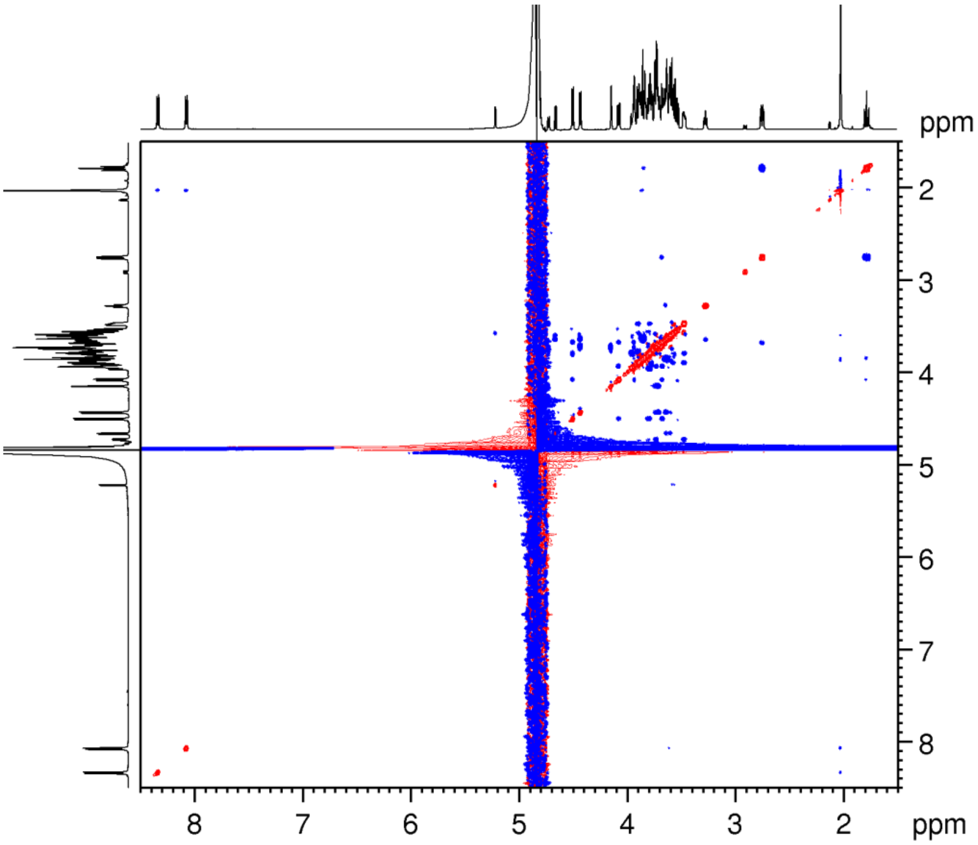

Figure S62. ^{1}H -^{1}H ROESY spectrum of LSTa (H_2O:D_2O 9:1 v/v solvent at pH 3.0)

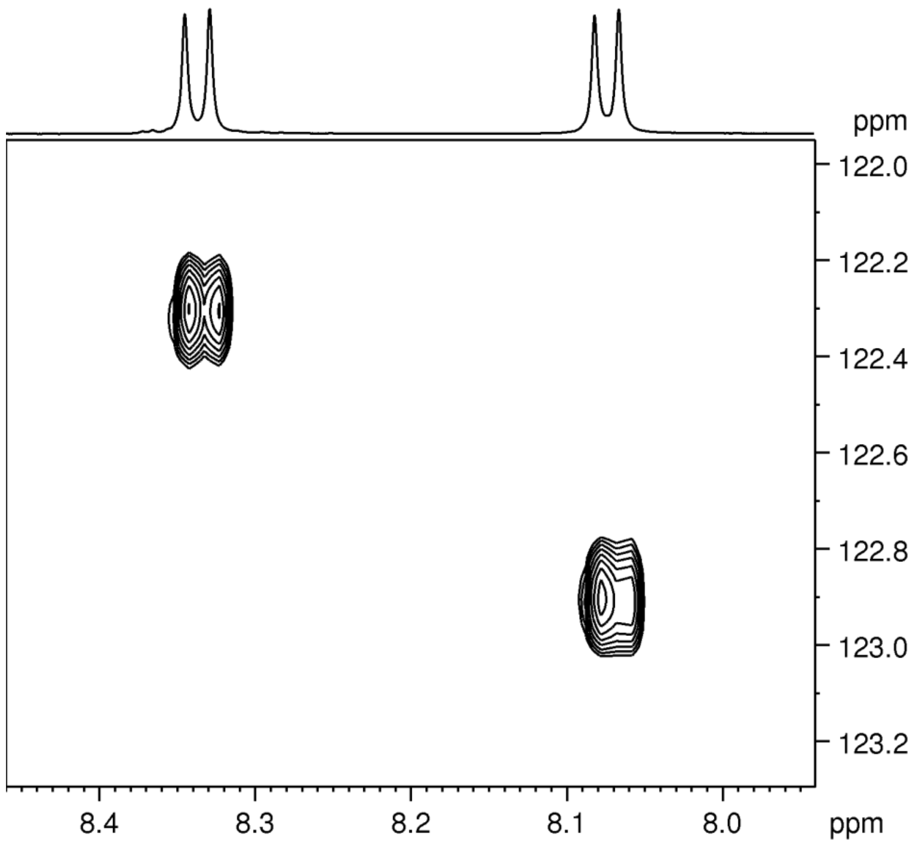

Figure S63. ^{1}H -^{15}N HSQC spectrum of LSTa (H_2O:D_2O 9:1 v/v solvent at pH 3.0)

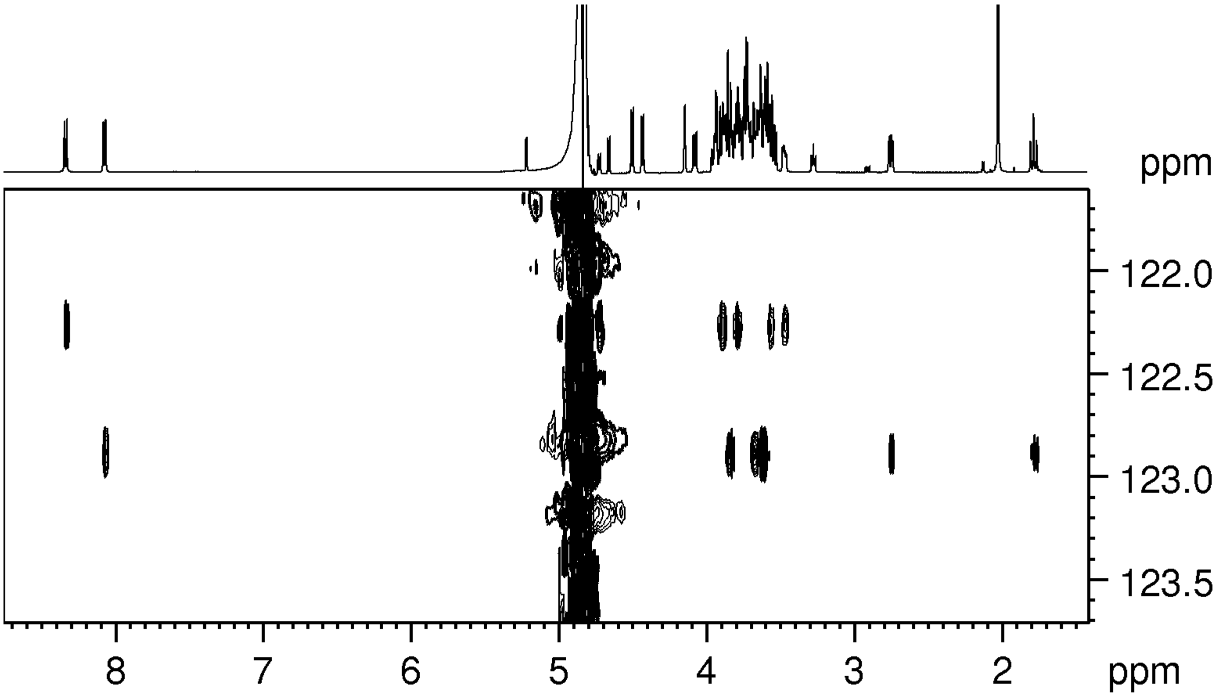

Figure S64. ^{1}H -^{15}N HSQC-TOCSY spectrum of LSTa (H_2O:D_2O 9:1 v/v solvent at pH 3.0)

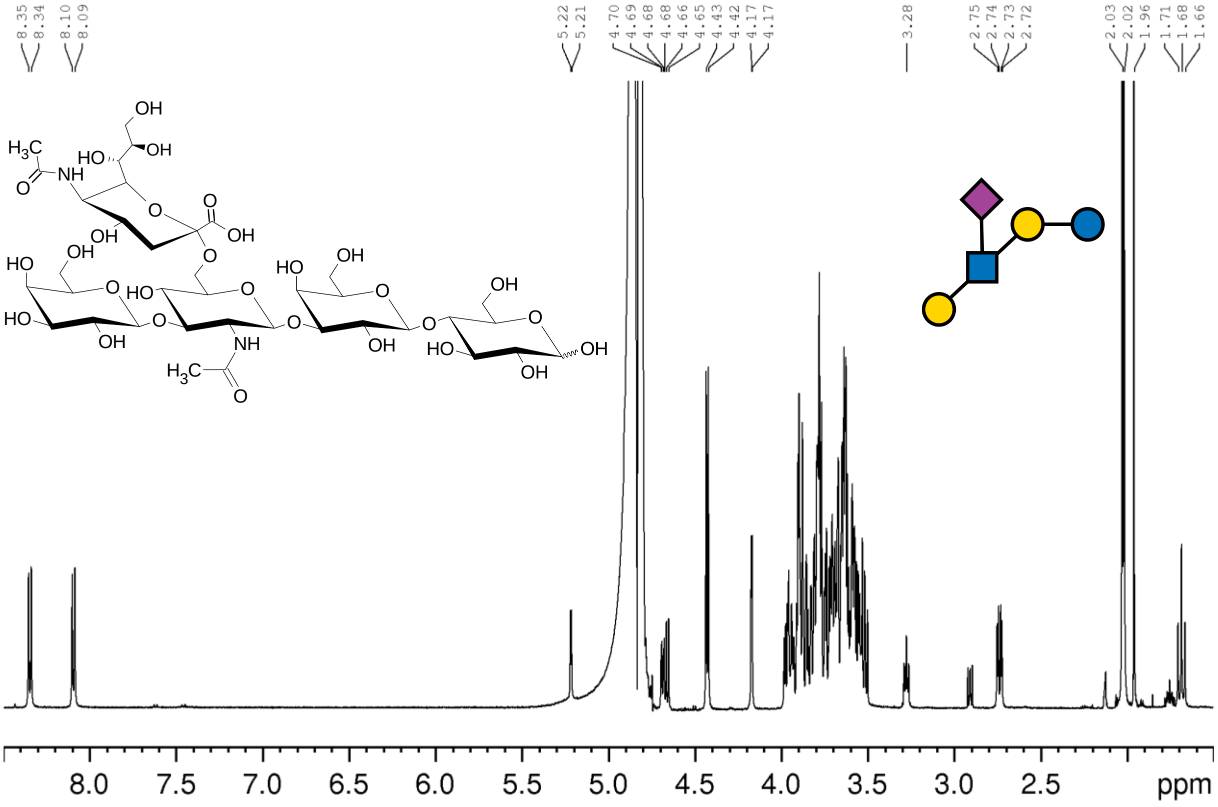

Figure S65. ^{1}H NMR spectrum of LSTb (H_2O:D_2O 9:1 v/v solvent at pH 3.0)

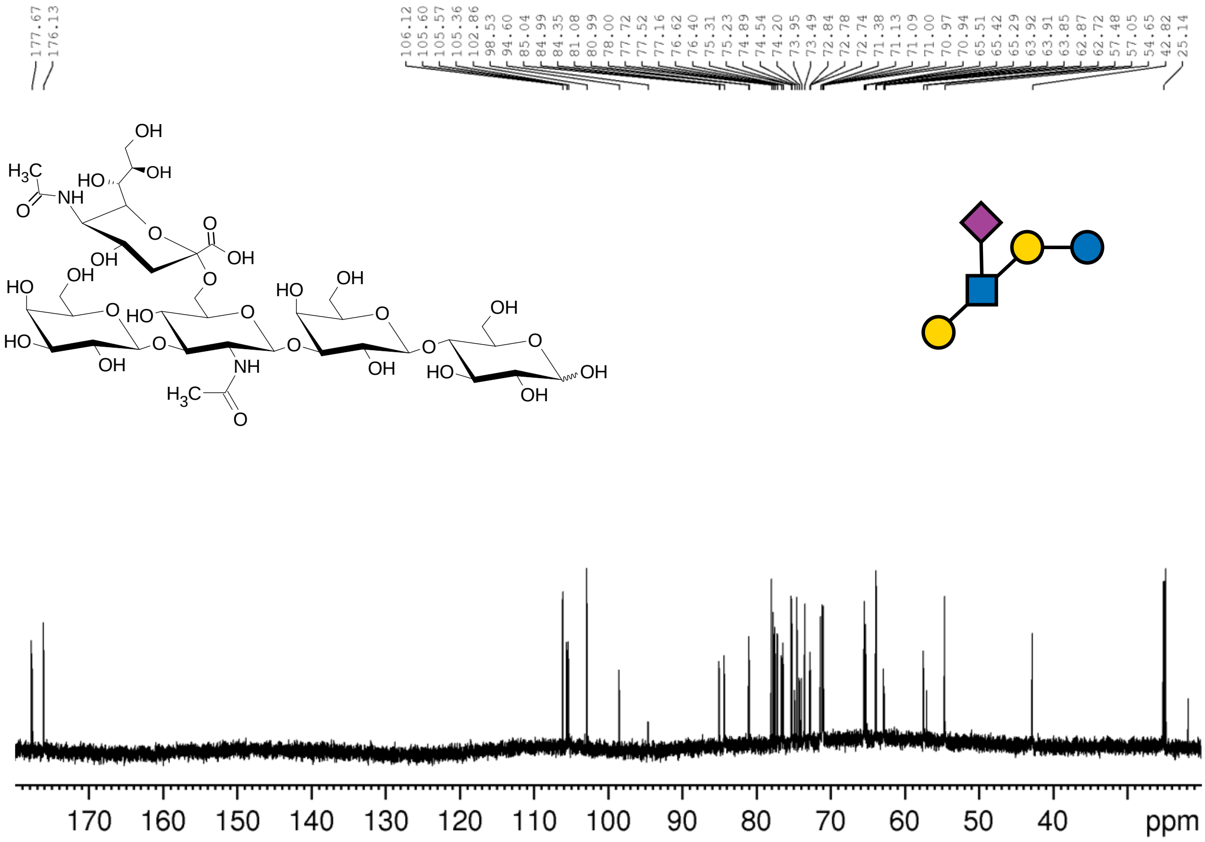

Figure S66. ^{13}C NMR spectrum of LSTb (H_2O:D_2O 9:1 v/v solvent at pH 3.0)

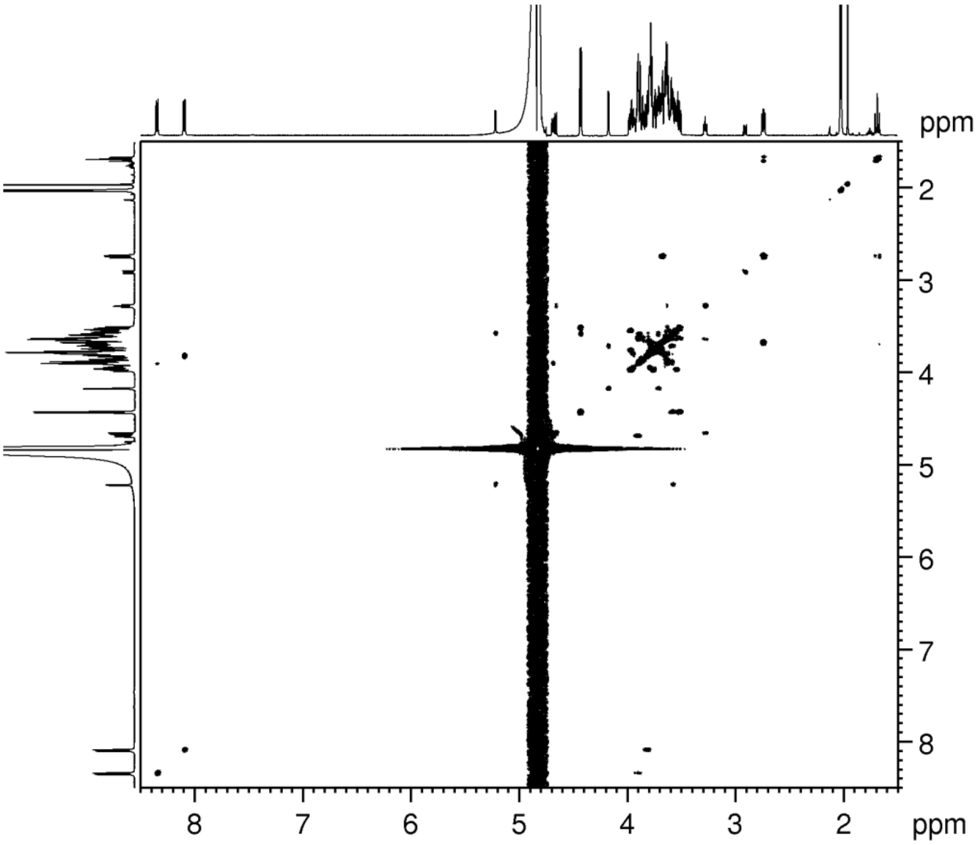

Figure S67. 1H -1H COSY spectrum of LSTb (H_2O:D_2O 9:1 v/v solvent at pH 3.0)

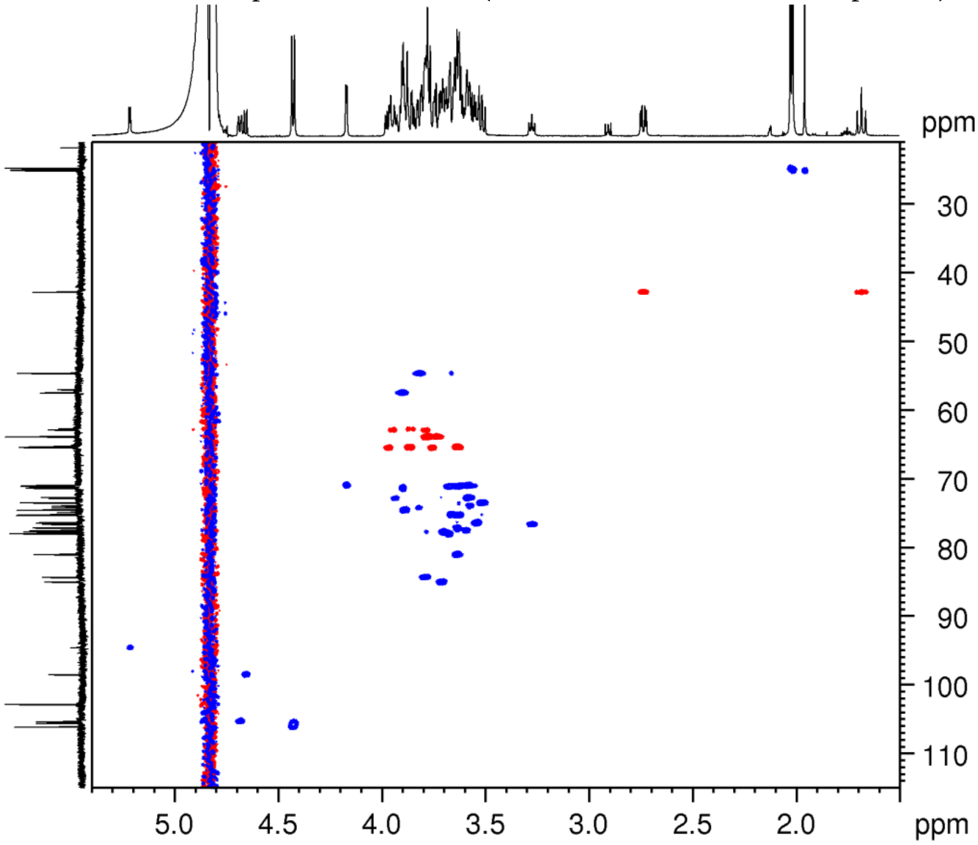

Figure S68. ^{1}H -^{13}C HSQC spectrum of LSTb (H_2O:D_2O 9:1 v/v solvent at pH 3.0)

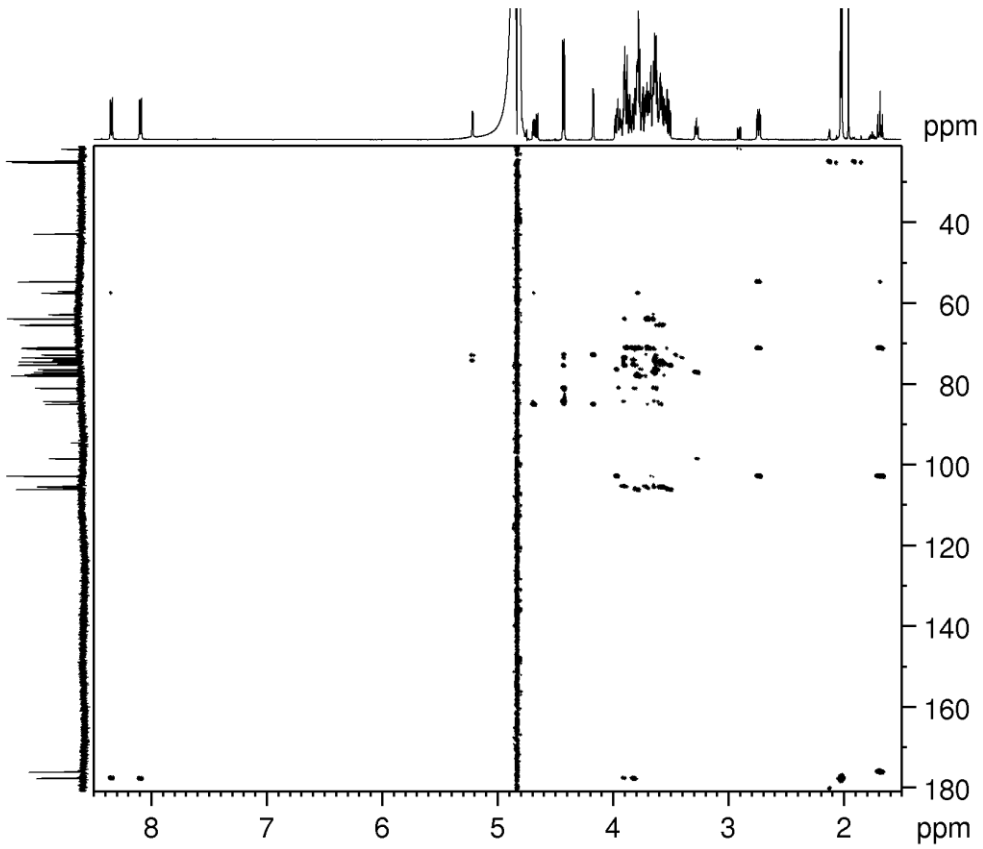

Figure S69. ^{1}H -^{13}C HMBC spectrum of LSTb (H_2O:D_2O 9:1 v/v solvent at pH 3.0)

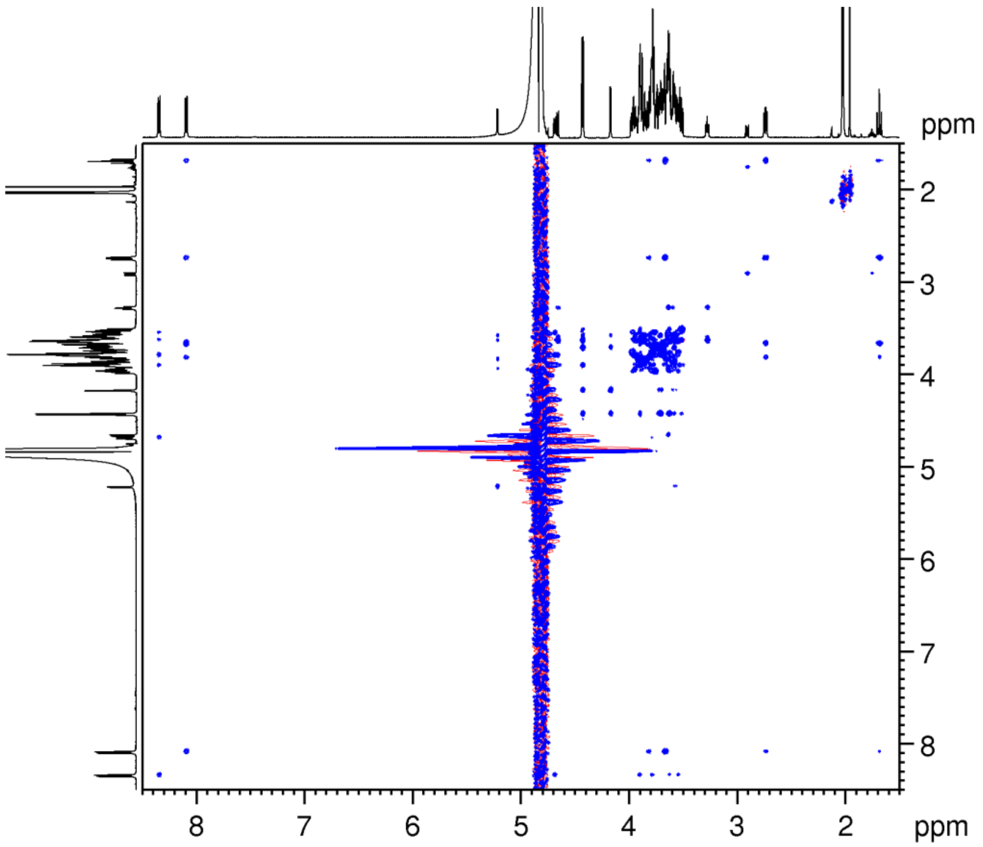

Figure S70. ^{1}H -^{1}H TOCSY spectrum of LSTb (H_2O:D_2O 9:1 v/v solvent at pH 3.0)

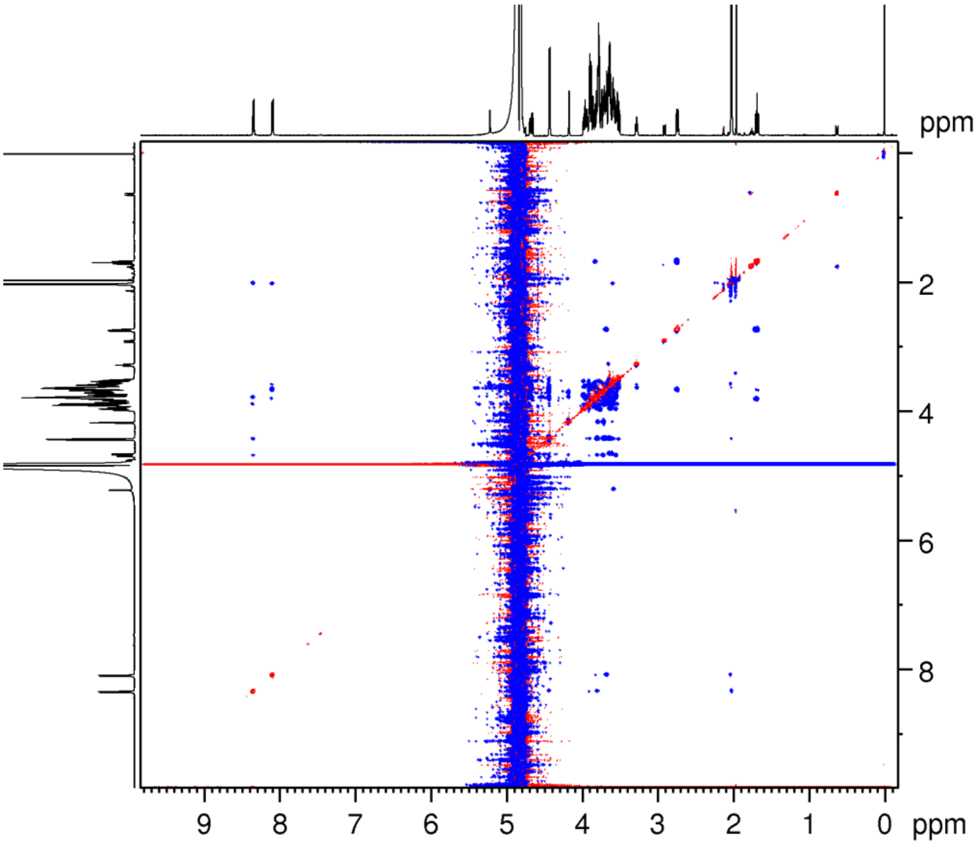

Figure S71. ^1H -^1H ROESY spectrum of LSTb (H_2O:D_2O 9:1 v/v solvent at pH 3.0)

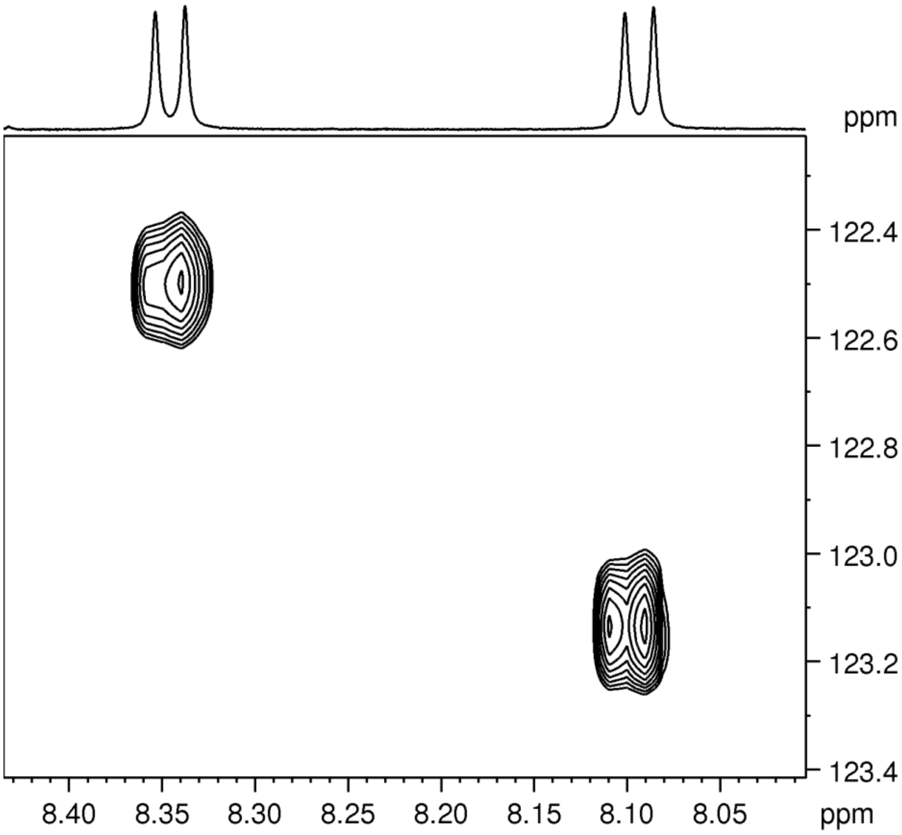

Figure S72. ^1H -^{15}N HSQC spectrum of LSTb (H_2O:D_2O 9:1 v/v solvent at pH 3.0)

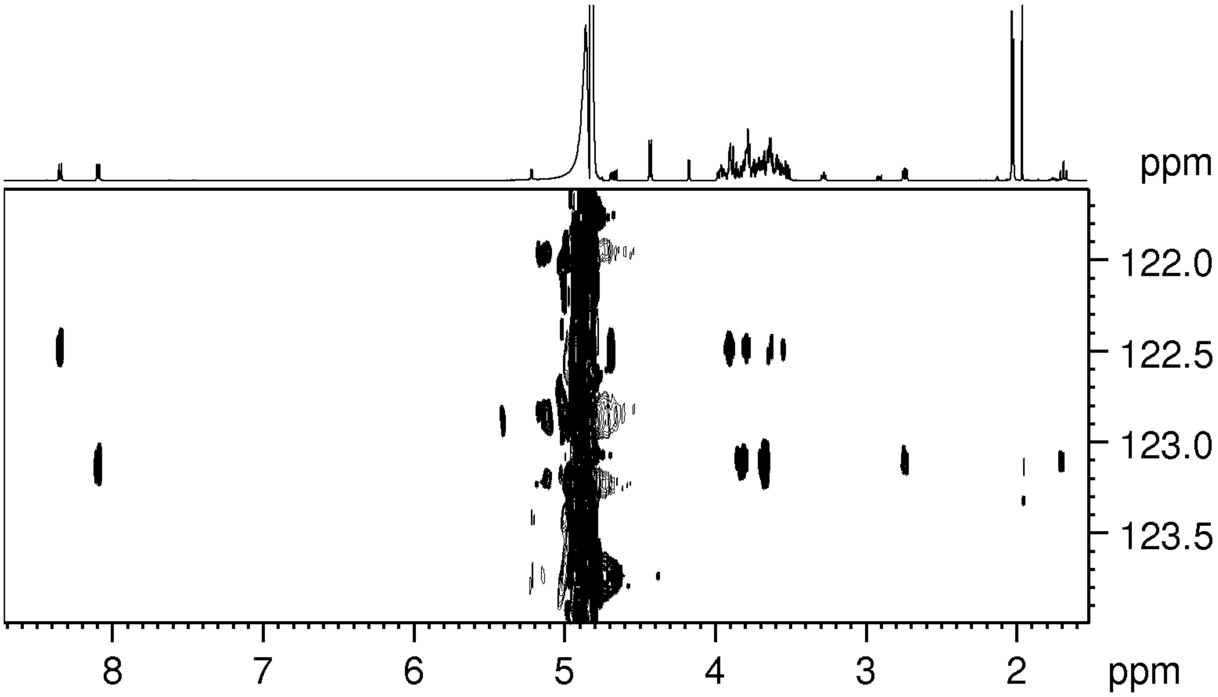

Figure S73. ^{1}H -^{15}N HSQC-TOCSY spectrum of LSTb (H_2O:D_2O 9:1 v/v solvent at pH 3.0)

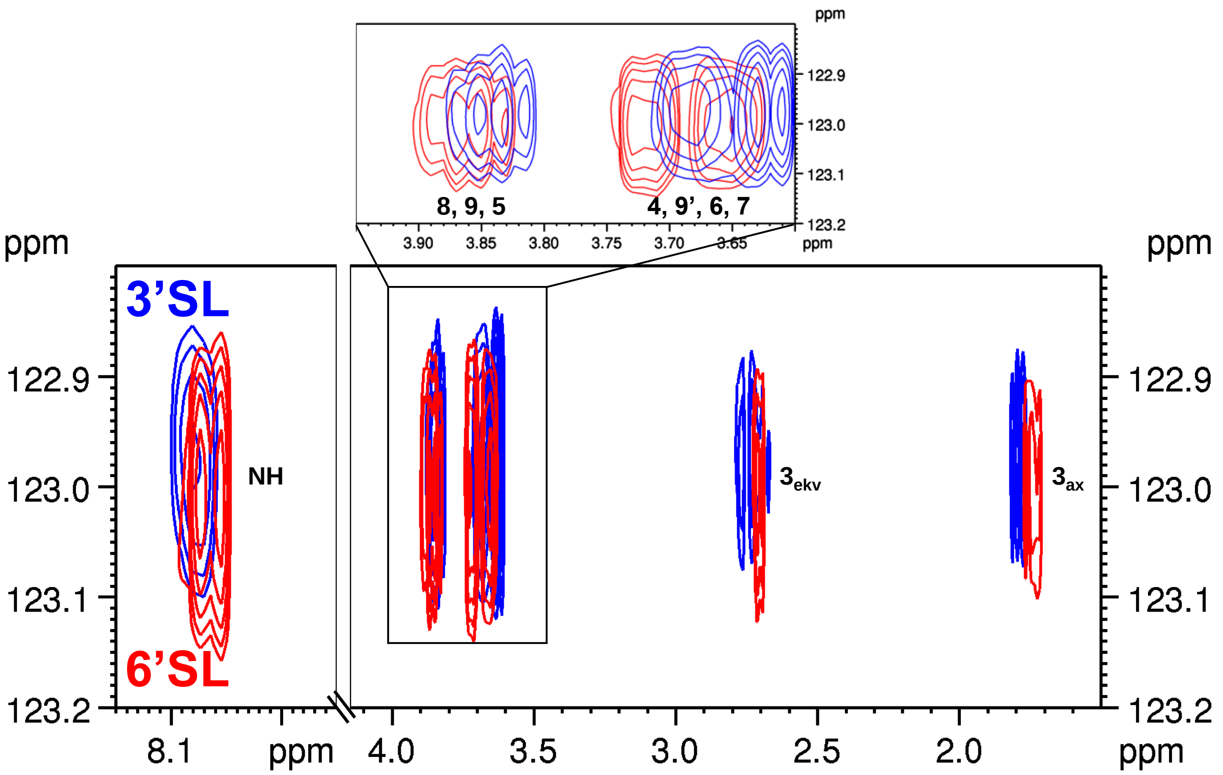

Figure S74. Overlaid ¹H-¹⁵N HSQC-TOCSY spectra of 3’SL and 6’SL with the ¹H NMR assignment of their Neu5Ac moiety

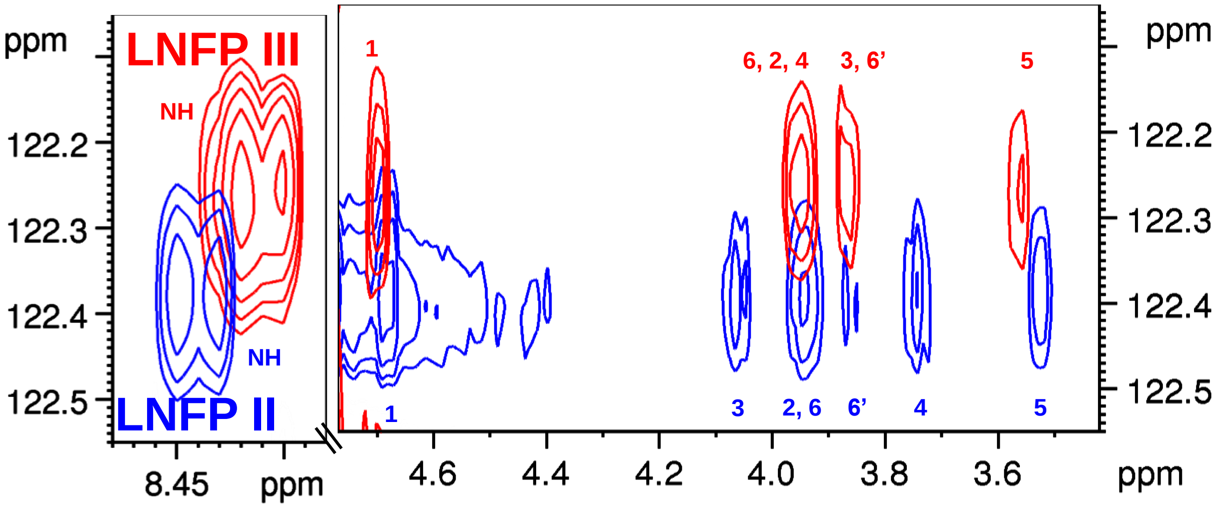

Figure S75. Overlaid ¹H-¹⁵N HSQC-TOCSY spectra of LNFP II and LNFP III with the ¹H NMR assignment of their GlcNAc moiety

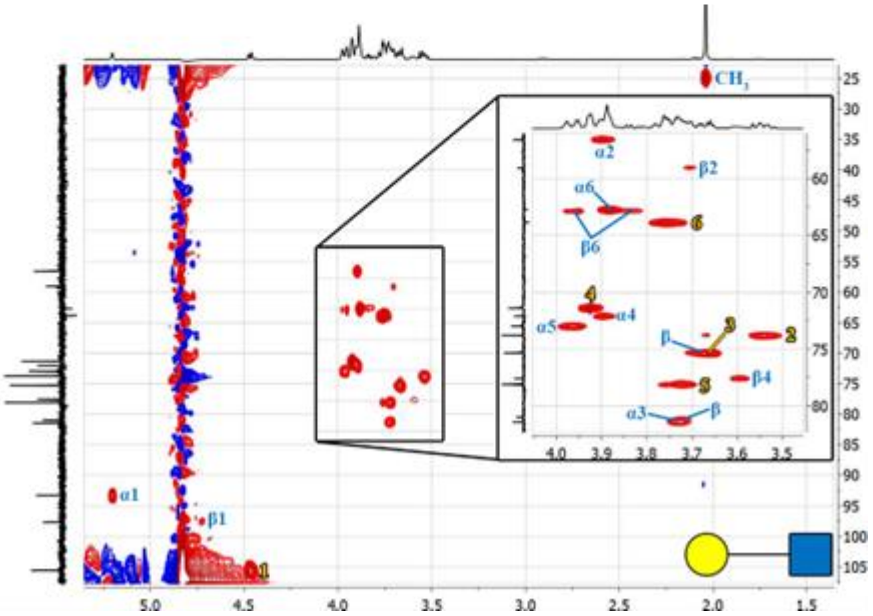

Figure S76. ^1H-^{13}C HSQC spectrum of LAcNAc

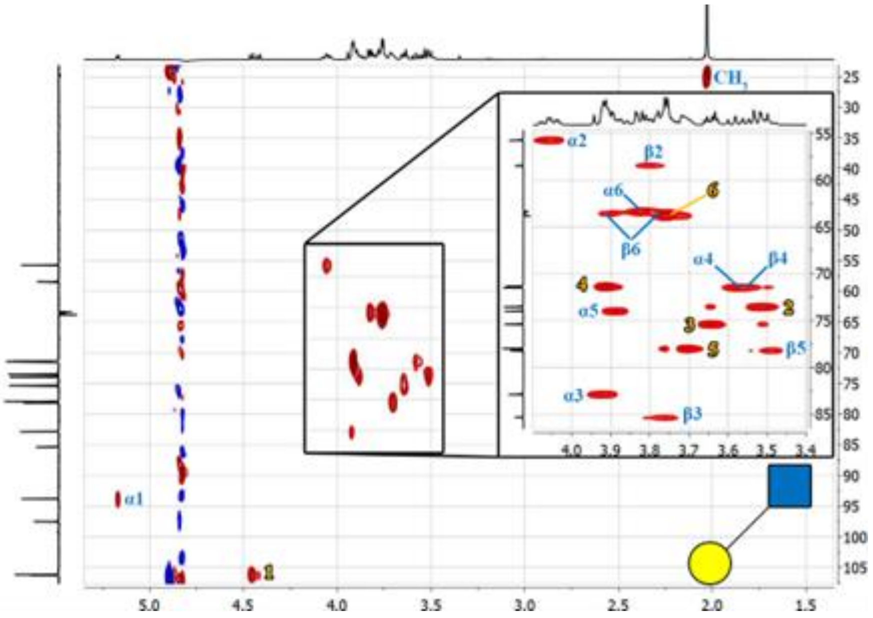

Figure S77. ^1H-^{13}C HSQC spectrum of LNB

Table S1. Complete resonance assignment of the disaccharides LacNAc and LNB

	**LacNAc**								**LNB**							
	**GlcNAc**						**Gal**		**GlcNAc**						**Gal**	
	**α**			**β**					**α**			**β**				
	**^{1}H**	**^{13}C**	**^{15}N**	**^{1}H**	**^{13}C**	**^{15}N**	**^{1}H**	**^{13}C**	**^{1}H**	**^{13}C**	**^{15}N**	**^{1}H**	**^{13}C**	**^{15}N**	**^{1}H**	**^{13}C**
**1**	5.20	93.3		4.73	97.7		4.46 / 4.47	105.6	5.17	93.8		o.l.	97.5		4.46/4.42	106.2/106.3
**2**	3.89	56.6		3.70	59.1		3.54	73.8	4.06	55.7		3.80	58.4		3.52	73.5/73.6
**3**	3.73	81.4		n.a.	n.a.		3.67	75.4	3.93	82.9		3.76	85.3		3.65	75.4
**4**	3.89	72.1		3.60	77.6		3.92	71.4	3.58	71.5		3.55	71.4		3.92	71.4
**5**	3.97	73.0		n.a.	n.a.		3.72	78.1	3.89	74.0		3.49	78.2		3.71	78.0
**6**	3.88	62.8		3.83<br>3.96	62.9		3.76	63.9	3.83	63.4		3.76<br>3.90	63.5		3.76	63.8
**NH**	**8.20**		**123.65**	**8.25**		**122.88**			**8.23**		**123.19**	**8.33**		**122.28**		

n.a.: not assigned, o.l.: overlapped

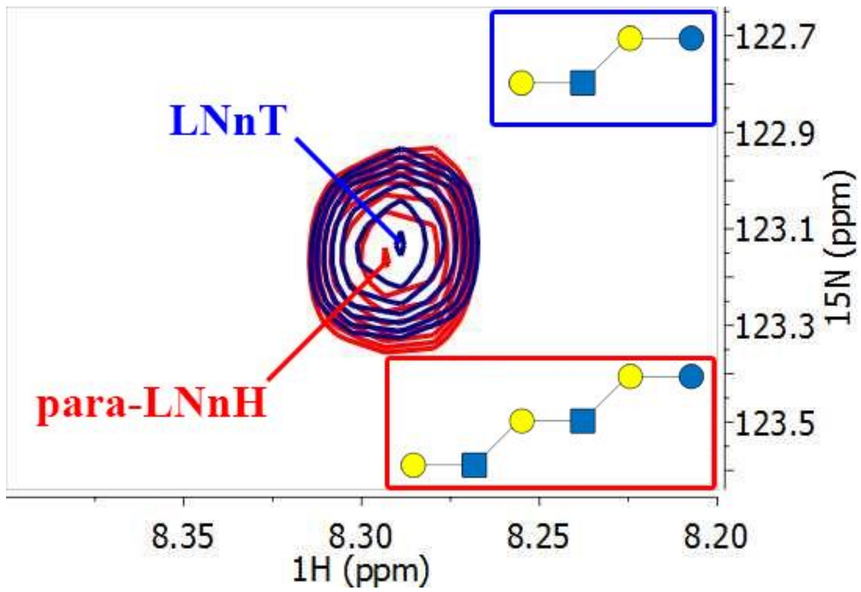

Figure S78. Overlaid ^{1}H-^{15}N HSQC spectra of LNnT and para-LNnH

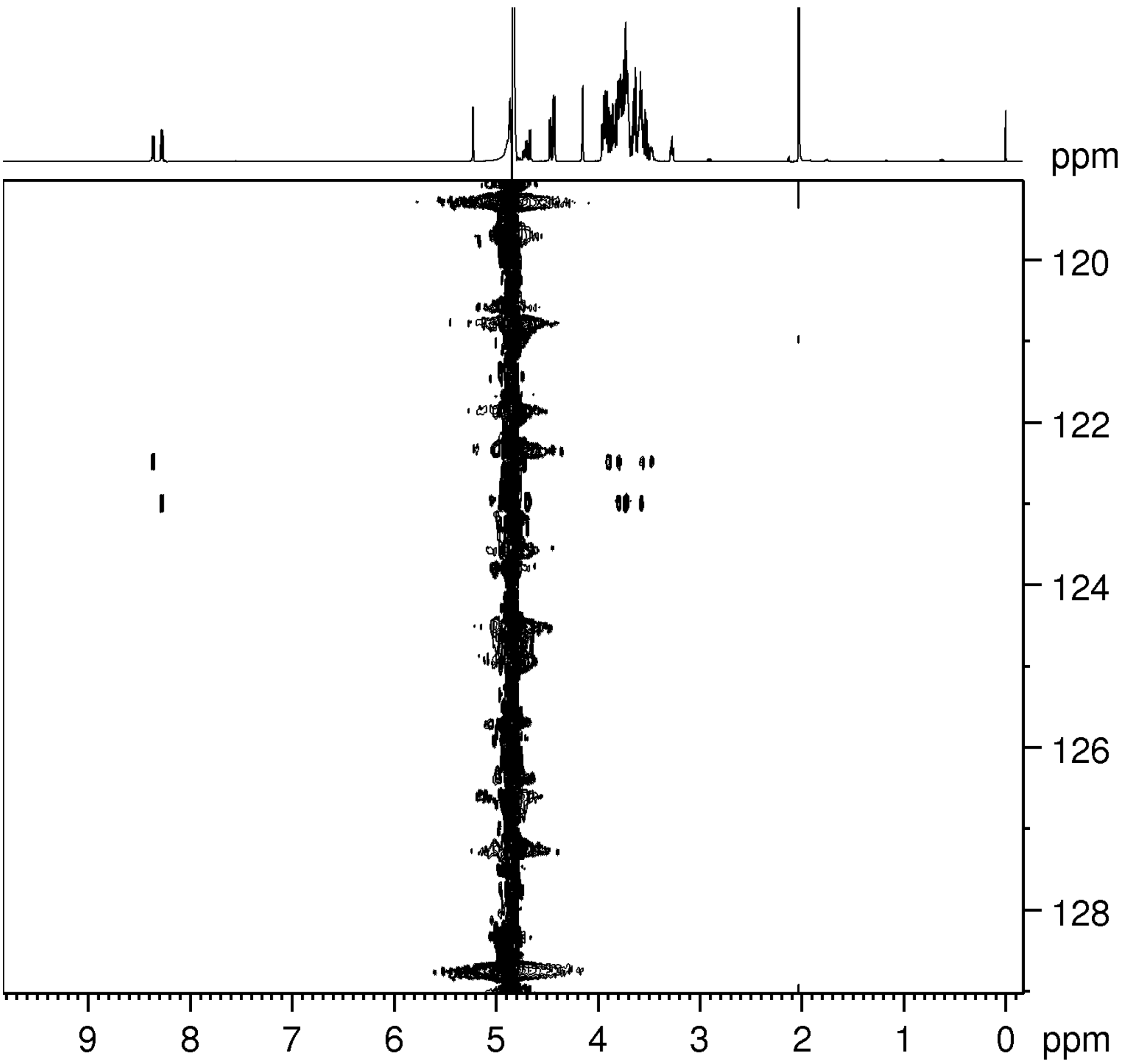

Figure S79. Full ^{1}H-^{15}N HSQC-TOCSY spectrum of LNT and LNnT mixture (1:1, H_2O:D_2O 9:1 v/v solvent at pH 3.0)

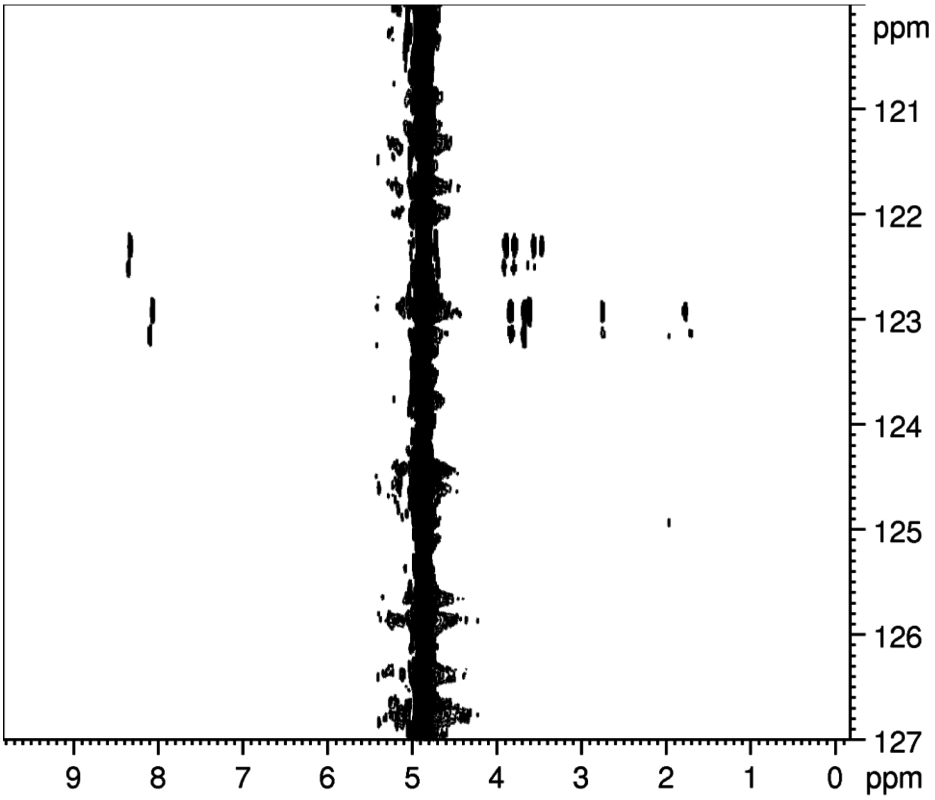

Figure S80. Overlaid full ^{1}H-^{15}N HSQC-TOCSY spectra of LSTa and LSTb.

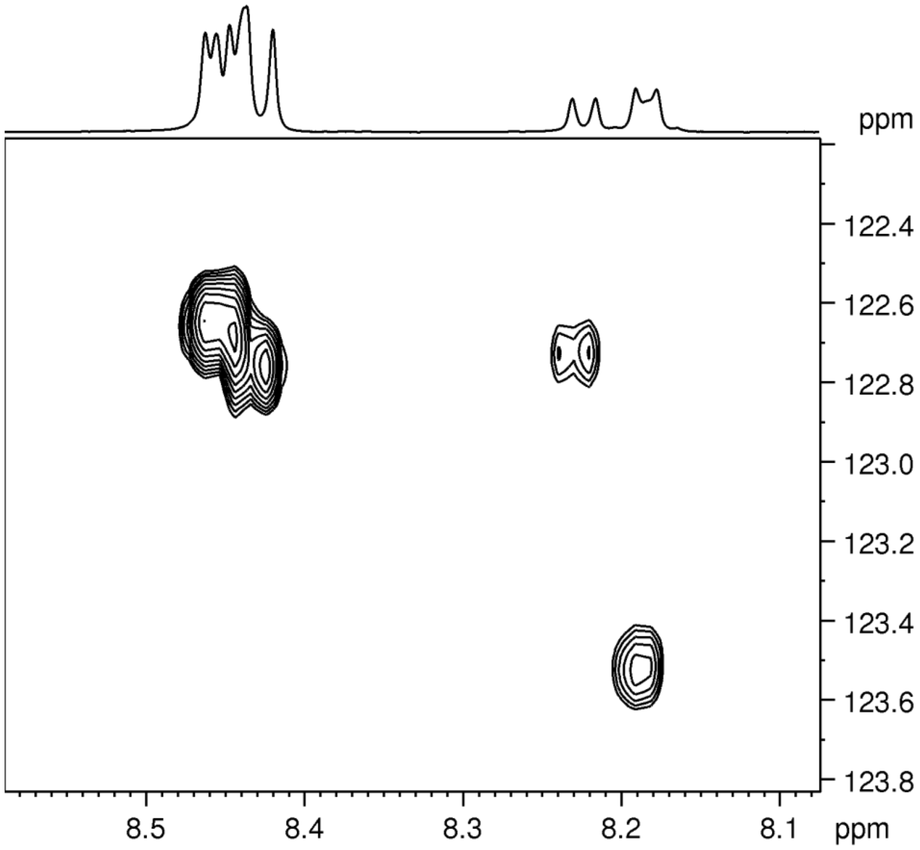

Figure S81. ^{1}H -^{15}N HSQC spectrum of *N,N',N'',N'''*-Tetraacetylchitotetraose (H_2O:D_2O 9:1 v/v solvent at pH 3.0)

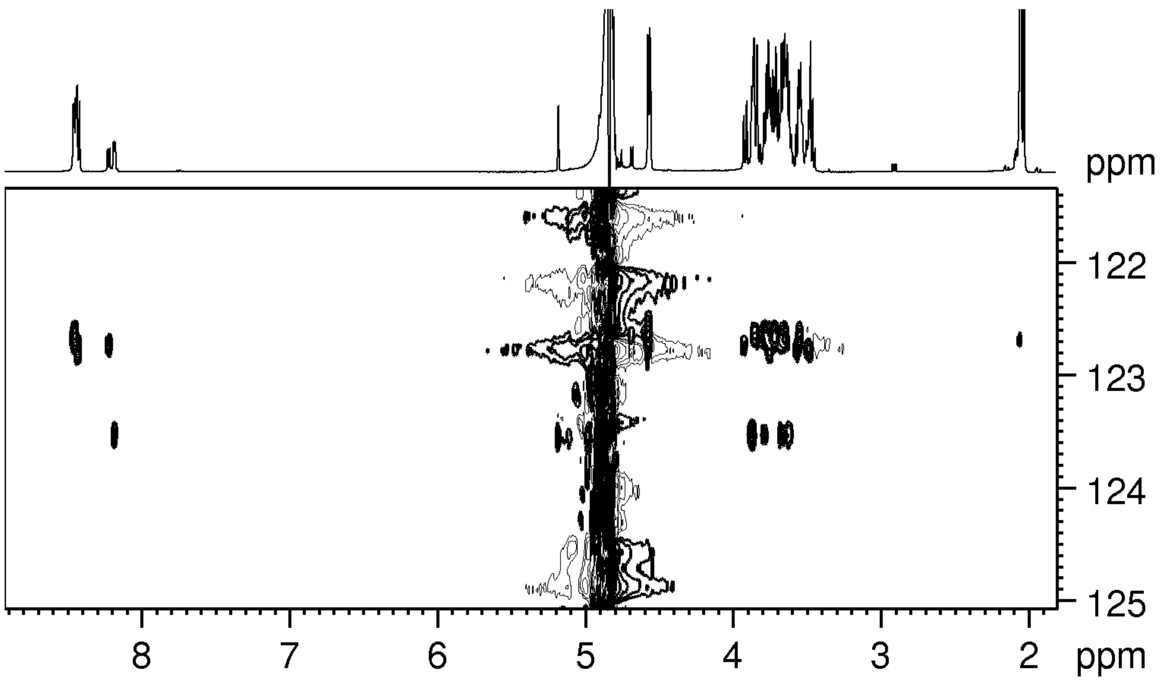

Figure S82. ^{1}H -^{15}N HSQC-TOCSY spectrum of *N*,*N*',*N*'',*N*'''-Tetraacetylchitotetraose (H_2O:D_2O 9:1 v/v solvent at pH 3.0)
